# Supplementary material for: Race and resource allocation: an online survey of US and UK adults’ attitudes toward COVID-19 ventilator and vaccine distribution
Source: BMJ Open. 2022 Nov 21;12(11):e062561. doi: 10.1136/bmjopen-2022-062561 (PMC9679868; doi:10.1136/bmjopen-2022-062561)
Supplement: Supplementary data [file bmjopen-2022-062561supp001.pdf]

Supplemental Materials: Public attitudes towards race and resource allocation in a pandemic

Andreas Kappes[1], Hazem Zohny[2], Julian Savulescu[2,3], Ilina Singh[4 5], Walter Sinnott-Armstrong[6], Dominic Wilkinson[2,3,7]

Affiliations:
[1]City, University of London
[2] Oxford Uehiro Centre for Practical Ethics, University of Oxford
[3] Murdoch Children's Research Institute, Melbourne, Australia.
[4] Wellcome Trust Centre for Ethics and the Humanities, University of Oxford
[5] Department of Psychiatry, University of Oxford
[6] Kenan Institute for Ethics and Philosophy Department, Duke University
[7] John Radcliffe Hospital, Oxford, UK

Corresponding author Email: hazem.zohny@philosophy.ox.ac.uk

Kappes A, *et al. BMJ Open* 2022; 12:e062561. doi: 10.1136/bmjopen-2022-062561

Supplemental Methods

Procedure

The survey was designed to assess participants' preferences for using medical factors as well as race, sex, obesity for allocating vaccines to people in need. Two surveys were conducted, one in the US and one in the UK. The surveys were adapted from a previous survey (4) on triage guidance involving ventilator allocations. Full survey materials can be found here: osf.io/w7bpq.

Vaccine Allocations

All participants were first introduced to the context of the allocation decisions. We wanted to make sure that all participants knew what would happen to the recipient that would not get vaccine, that all recipients wanted to get the vaccine, and that the vaccine was effective and safe. Participants read:

> *For the following scenarios, imagine you are working in a clinic. You are in charge of deciding which patients should get vaccinated first against COVID-19. You have one remaining dose of the vaccine left, and you must choose between two patients. The unvaccinated patient must wait several months before stocks are replenished. For the purpose of this survey, please think of the vaccine as if it had a 100% success rate in preventing COVID-19 and no side effects whatsoever. And each person under consideration wants to get vaccinated.*

Participant then learned that the estimate for each recipient's chance of getting severe COVID-19 was high and was based on their age and medical history. Furthermore, they learned that their task was to decide whom to give the vaccine.

Risk of developing severe COVID-19. To determine how much weight participants would give the risk of developing severe COVID-19 for vaccine allocations, participants answered five scenarios in which the potential recipients only differed in their chance of developing severe COVID-19. In one scenario, one patient had an 80% chance and the other one a 10% chance of developing severe COVID-19 (other scenarios: 40% versus 10%, 20% versus 10%, 15% versus 10% and 10% versus 10%). Thereafter, participants were randomly introduced to one of the three additional factors: race, sex, and obesity.

Racial minority scenarios. For a racial minority in the US, we chose Black recipients and, in the UK, BAME recipients. BAME is an umbrella term, common in the United Kingdom, used to describe non-white racial groups. While a contested term, it was most consistently used in media reports on the pandemic in the UK (e.g., 5). Before participants had to decide if they wanted to give the vaccine to the member of the racial minority, we ensured that all participants were aware of the inequality in COVID-related deaths in the UK and in the US. UK participants read: "*According to the Office for National Statistics, individuals belonging to Black, Asian, and minority ethnic (BAME) groups have in general been shown to die from COVID-19 at a higher rate than that of White individuals.*" US participant read: "*According to the Centre for Disease Control and Prevention, Black Americans have been shown to die from COVID-19 at a higher*

rate than of White Americans." Thereafter, participants worked on five scenarios. All of these scenarios involved one Black (BAME) and one White patient. In one scenario, both patients had the same risk of illness (10% versus 10%), in two scenarios the Black (BAME) patient had a higher risk of severe COVID-19 (5% and 10% higher), and in two scenarios the White patient a higher risk (5% and 10% higher). Participants indicated for each whether they wanted to give the vaccine to the racial minority patient, the white patient, or toss a coin.

Sex and obesity scenarios. Participants saw a similar set of scenarios for sex-based and obesity-based allocation decisions. Before working on the scenarios that involved men and women, participants read: "*According to scientific studies, men have been shown to die from COVID-19 at a higher rate than that of women."* The rationale was to inform participants that being male may be predictive of a worse outcome or survival chance if infected with COVID-19 (6). And before working on the scenarios involving obesity, participants read: "*According to scientific studies, people who are obese (severely overweight) have been shown in general to die from COVID-19 at a higher rate than that of people who have a healthy weight."* The rationale for presenting participants with this was to inform them about obesity being predictive of a worse outcome or survival chance if infected with COVID-19 (7). The five obesity-related and five sex-related scenarios followed the exact same structure as the race-related scenarios.

Group scenarios. To test if it would make a difference if participants considered only one patient, or a group of patients, we repeated the same set of scenarios for race, sex, and obesity for groups of patients. The reason for including group scenarios was twofold. First, there is a large body of evidence that suggest that people have different moral intuitions when they think about one persons versus a group of people in need (8). Second, one might argue that people might feel that the single recipient scenario for vaccine distributions is unrealistic since such decisions most often are made for large groups, rather than individuals. To ensure that such perceptions did not impact allocation preferences, we additionally included group scenarios. The scenarios followed the exact same structure as the individual ones but instead of deciding between two patients, participants had to decide between two groups of patients. As an introduction to this section of the survey, participants were told:

> *Imagine you are a policymaker for the government. At the moment, the number of vaccines available is limited and you do not have enough to protect everyone. Sometimes, policy makers face difficult decisions about which subsets of the population should be given a vaccine and which shouldn't. You will make decisions about whether or not to provide vaccines to one of two groups of people. If the people in this survey are not vaccinated, they are likely to get COVID-19 and suffer serious health consequences as a result. The group you decide not to vaccinate must risk waiting several months until stocks are replenished.*

Race, sex, and obesity scenarios. The structure of each of these scenario groups followed the structure for the respective vaccine scenario. Participants were informed about the effect race, sex, and obesity had on COVID-related deaths before working on the corresponding set of scenarios (see above). In the five race-related scenarios, participants both patients had the same chances of survival (40% versus 40%), in two scenarios the Black (BAME) patient had a higher chance of survival (10% and 20% higher), and in two scenarios the White patient a higher risk

Kappes A, *et al. BMJ Open* 2022; 12:e062561. doi: 10.1136/bmjopen-2022-062561

chance of survival (10% and 20% higher). Participants indicated for each whether they wanted to give the vaccine to the racial minority patient, the white patient, or toss a coin. The scenarios related to sex and obesity followed the exact structure as the race-related scenarios.

Prioritization Attitude Measure. For each of the sets of scenarios, we created an attitude score by counting the number of times participants decided to give the vaccine to the person with an additional risk factor on each of the five respective scenarios. For instance, a participant that would always give the vaccine to the recipient from the racial minority would have an attitude score of 5, a participant who would allocate solely by the risk of contracting severe COVID-19 would get a 0, and a person that would always give priority to the white recipient would get a score of -5. We did this for all the patient and all the group scenarios.

Perceptions of Injustice, Responsibility, Stereotypes, and Racism

After participants worked on the scenarios, we asked them a series of questions to capture their perceptions of racial minorities, men, and obese people.

Perceptions of injustice, responsibility. To measure as how unjust people perceived the higher COVID-related death rates, we asked participants to indicate their agreement with the following statement: "Injustice was the main reason [category] individuals died more often than individuals from other groups". To measure perceptions of responsibility of the group for the higher death rates, we asked participants to indicate their agreement with the following statement: "The main reason [category] die more often from COVID-19 is due to factors outside their control." For both questions, scales ranged from 1 (strongly disagree) to 5 (strongly agree).

Stereotypes. According to the stereotype content model, perceptions of groups can be described along two fundamental dimensions: warmth and competence (9, 10). In line with previous research, we measured warmth by how warmly participants felt towards black (US) or BAME (UK) people, men, and obese people. We measured competence by asking participants how competent they perceived black (US) or BAME (UK) people, men, and obese people to be. We used 100-point scales for participants to indicate their perceptions.

Covert Racism - Modern Racism Scale. The modern racism scale is intended to capture subtle or covert racial attitudes or the endorsement of the idea that racial minorities are getting undeserved attention (1). Although modern racist potentially do not perceive themselves as racist, attitudes guide their decisions in situations that provide nonracially motivated reasons. Hence, the modern racism scale provides seven statements that one could endorse or reject (e.g., "Discrimination against Blacks is no longer a problem in the United States" and "Over the past few years, Blacks have gotten more economically than they deserve"). Participants are asked to express their agreement of each statement via a 5-point scale, ranging from 1 (Strongly disagree) to 5 (Strongly agree). Extensive previous research has found that scores on the modern racism scale correlate with anti-Black affect, right-wing authoritarianism, social dominance orientation, and indices of old-fashioned racism (11).

Statistical Analyses (Fixed Linear Models)

We ran a series of 4 linear models to explore the role of various factors in predicting racial prioritization attitudes. For each model, we defined attitudes as dependent variable and subjects as the random factor. In the first two models, we entered age, gender, race, relative income, educational level, political ideology (general, social, and economical), and type of scenario (patient versus group-based) as fixed factors. In the second two models, we additionally added perceptions of injustice, responsibility, warmth, competence, and modern racism scores (see results above).

Supplemental Results

1. Group Preferences for Vaccine and Ventilator Preferences

Patterns of response were similar for scenarios involving groups as for individual patients (Figure 2 for individual preferences, Figure S1 for group preferences).

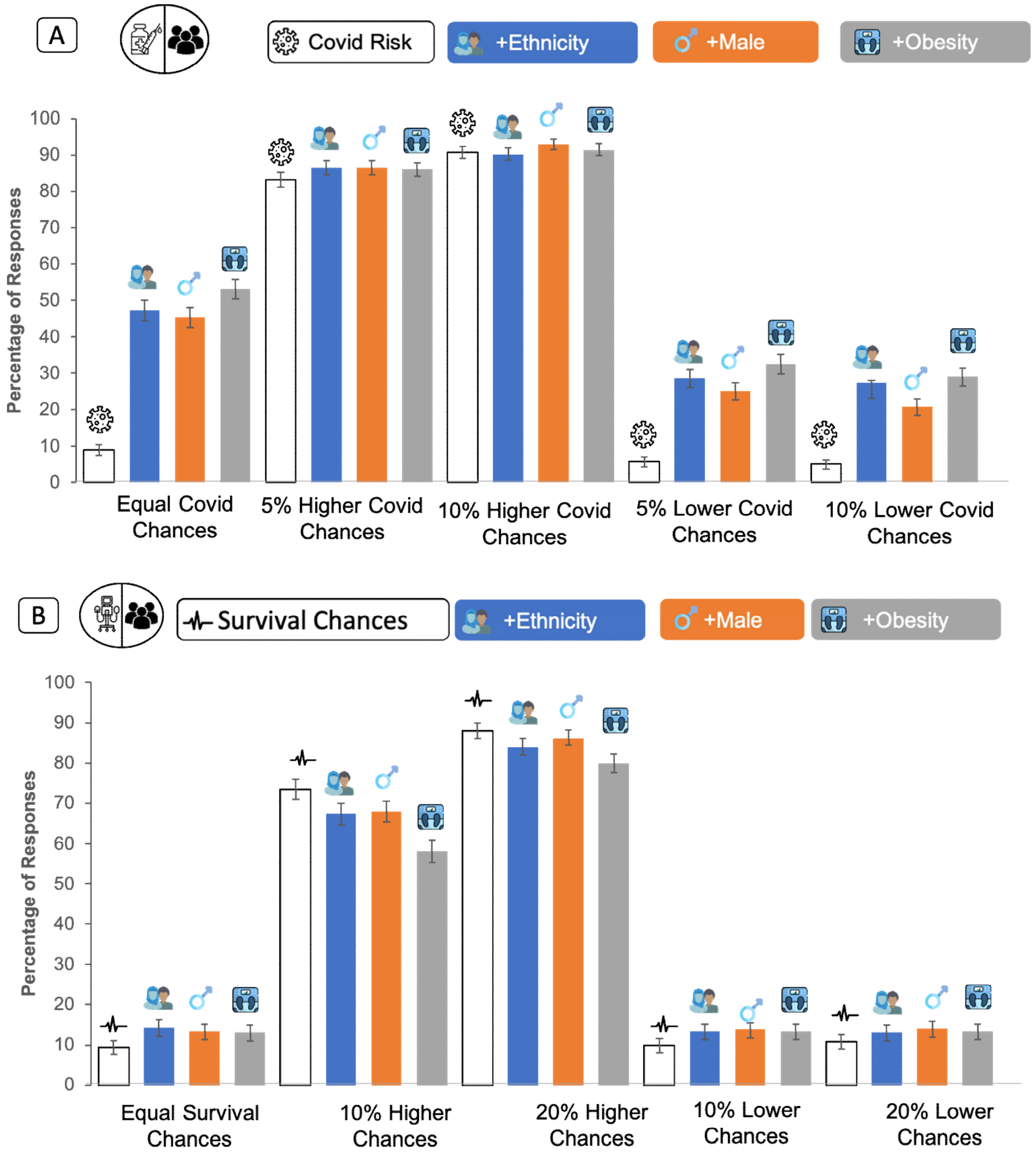

Supplementary Figure S1. A: Preferences for prioritization of vaccines. Response to scenarios where competing individual patients differed in medical risk alone or in medical risk plus an additional risk factor (race, sex, or obesity), where the patient with the additional risk factor had a 5% or 10% higher or lower chance of getting severe Covid based on medical risk alone. The bars indicate the proportion of respondents who would allocate the vaccine to a patient with a particular characteristic and risk profile. **B: Preferences for prioritization of ventilators.** A: Response to scenarios where competing individual patients differed in medical risk alone (Equal COVID chance), or in medical risk plus an additional risk factor (race, sex, or obesity), where the patient with the additional risk factor had a 10% or 20% higher or lower chance of dying based on medical risk alone. The bars indicate the proportion of respondents who would allocate the ventilator to a patient with a particular characteristic and risk profile.

3. Responses of Black American participants for all scenarios involving ventilators and race in true percent

	Black American Participants		
	Black Patient	Coin Toss	White Patient
Equal Survival Chances	29.5	52.5	18.0
10% Higher Chance	55.7	18.0	26.2
30% Higher Chance	62.3	1.6	31.1
10% Lower Chance	32.8	13.1	54.1
30% Lower Chance	34.4	3.3	62.3

4. Responses of Black American participants for all scenarios involving vaccines and race true precent

	Black American Participants		
	Black Patient	Coin Toss	White Patient
Equal Covid Chances	49.1	41.5	9.4
5% Higher Chance	86.8	1.9	11.3
10% Higher Chance	86.8	1.9	11.3
5% Lower Chance	35.8	3.8	60.4
10% Lower Chance	34	1.9	64.2

5. General Linear Mixed Models

To test which variables impacted participants' attitudes towards risk factors, we ran a series of four linear models to explore the role of various factors in predicting racial prioritization attitudes. For each model, we defined attitudes as dependent variable and subjects as the random factor. In the first two models, we entered age, gender, race, relative income, educational level, political ideology (general, social, and economical), and type of scenario (patient versus group-based) as fixed factors. In the second two models, we additionally added perceptions of injustice, responsibility, warmth, competence, and modern racism scores. The full results of each model can be seen in Table S1.

Table S1. Linear Mixed Models

**Information Criteria**	**US: Vaccination Simple Model**	**US: Vaccination Full Model**	**UK: Vaccination Simple Model**	**UK: Vaccination Full Model**
-2 Log Likelihood	5253.189	4906	4846.48	4382.353
Akaike's Information Criterion (AIC)	5305.189	4968	4892.48	4438.353

Hurvich and Tsai's Criterion (AICC)	5306.273	4970	4893.34	4439.724
Bozdogan's Criterion (CAIC)	5466.048	5158	5034.64	4609.2
Schwarz's Bayesian Criterion (BIC)	5440.048	5127	5011.64	4581.2

**Information Criteria**	**US: Ventilator Simple Model**	**US: Ventilator Full Model**	**UK: Ventilator Simple Model**	**UK: Ventilator Full Model**
-2 Log Likelihood	4793.874	4733.97	3845.598	3758.101
Akaike's Information Criterion (AIC)	4847.874	4797.97	3893.598	3816.101
Hurvich and Tsai's Criterion (AICC)	4849.092	4799.68	3894.575	3817.529
Bozdogan's Criterion (CAIC)	5013.837	4994.616	4040.817	3993.85
Schwarz's Bayesian Criterion (BIC)	4986.837	4962.616	4016.817	3964.85

**American Sample: Vaccination Allocation**				
	**Numerator df**	**Denominator df**	**F**	**Sig.**
Intercept	1	661	0.129	0.72
Scenario Type (Group or Patient)	1	661	63.748	< .0001
Age	1	661	0.408	0.523
Race (US)	5	661	1.706	0.131
Gender	3	661	7.238	< .0001
Highest Schooling	8	661	1.157	0.323
Social Status	1	661	0.089	0.765
Household Income	1	661	0.726	0.394
Political Orientation (General)	1	661	3.32	0.069
Political Orientation (Economical)	1	661	0.526	0.469
Political Orientation (Social)	1	661	11.382	0.001

**UK Sample: Vaccination Allocation**

Kappes A, *et al. BMJ Open* 2022; 12:e062561. doi: 10.1136/bmjopen-2022-062561

	Numerator df	Denominator df	F	Sig.
Intercept	1	657	1.544	0.214
Scenario Type (Group or Patient)	1	657	91.289	< .0001
Age	1	657	0.233	0.63
Race (UK)	5	657	1.212	0.302
Gender	1	657	0.272	0.602
Highest Schooling	7	657	0.762	0.62
Social Status	1	657	1.985	0.159
Household Income	1	657	0.013	0.909
Political Orientation (General)	1	657	0	0.988
Political Orientation (Economical)	1	657	0.138	0.71
Political Orientation (Social)	1	657	0.244	0.621

American Sample: Vaccination – Full Model

	Numerator df	Denominator df	F	Sig.
Intercept	1	621	0.371	0.543
Scenario Type (Group or Patient)	1	621	61.082	< .0001
Age	1	621	1.171	0.28
Race (US)	5	621	1.328	0.25
Gender	3	621	4.702	0.003
Highest Schooling	8	621	0.678	0.711
Social Status	1	621	0.16	0.689
Household Income	1	621	0.096	0.756
Political Orientation (General)	1	621	5.125	0.024
Political Orientation (Economical)	1	621	0.008	0.927
Political Orientation (Social)	1	621	6.729	0.01
Injustice Perception	1	621	0.641	0.424
Responsibility Perception	1	621	2.589	0.108
Warmth Perception	1	621	0.012	0.912
Competence Perception	1	621	1.488	0.223

Kappes A, *et al. BMJ Open* 2022; 12:e062561. doi: 10.1136/bmjopen-2022-062561

Modern Racism	1	621	23.16	< .0001

**UK Sample: Vaccination – Full Model**				
	**Numerator df**	**Denominator df**	**F**	**Sig.**
Intercept	1	607	0.809	0.369
Scenario Type (Group or Patient)	1	607	89.533	< .0001
Age	1	607	3.054	0.081
Race (UK)	1	607	0.023	0.879
Gender	7	607	1.176	0.315
Highest Schooling	1	607	2.438	0.119
Social Status	1	607	0.091	0.763
Household Income	1	607	0.304	0.582
Political Orientation (General)	1	607	0.171	0.68
Political Orientation (Economical)	1	607	1	0.318
Political Orientation (Social)	5	607	0.96	0.442
Injustice Perception	1	607	0.027	0.87
Responsibility Perception	1	607	14.238	< .0001
Warmth Perception	1	607	0.048	0.826
Competence Perception	1	607	7.638	0.006
Modern Racism	1	607	16.986	< .0001

**American Sample: Ventilator**				
	**Numerator df**	**Denominator df**	**F**	**Sig.**
Intercept	1	635	4.907	0.027
Scenario Type (Group or Patient)	1	635	2.418	0.12
Age	1	635	1.195	0.275
Race (US)	6	635	1.386	0.218
Gender	3	635	3.6	0.013
Highest Schooling	8	635	1.795	0.075
Social Status	1	635	4.437	0.036

Household Income	1	635	12.582	0
Political Orientation (General)	1	635	0.043	0.836
Political Orientation (Economical)	1	635	0.011	0.917
Political Orientation (Social)	1	635	0.976	0.324

UK Sample: Ventilator

	Numerator df	Denominator df	F	Sig.
Intercept	1	627	12.266	0
Scenario Type (Group or Patient)	1	627	1.713	0.191
Age	1	627	5.755	0.017
Race (UK)	4	627	7.181	0
Gender	3	627	0.342	0.795
Highest Schooling	7	627	0.521	0.819
Social Status	1	627	0.514	0.474
Household Income	1	627	0.816	0.367
Political Orientation (General)	1	627	0.793	0.373
Political Orientation (Economical)	1	627	0.002	0.962
Political Orientation (Social)	1	627	0.26	0.611

US Sample: Ventilator

	**Numerator df**	**Denominator df**	**F**	**Sig.**
Intercept	1	634	2.806	0.094
Scenario Type (Group or Patient)	1	634	2.418	0.12
Age	1	634	0.757	0.385
Race (UK)	6	634	0.897	0.497
Gender	3	634	3.411	0.017
Highest Schooling	8	634	1.624	0.115
Social Status	1	634	3.699	0.055

Kappes A, *et al. BMJ Open* 2022; 12:e062561. doi: 10.1136/bmjopen-2022-062561

Household Income	1	634	11.868	0.001
Political Orientation (General)	1	634	0.039	0.843
Political Orientation (Economical)	1	634	0.785	0.376
Political Orientation (Social)	1	634	0.047	0.829
Injustice Perception	1	634	0.778	0.378
Responsibility Perception	1	634	0.382	0.537
Warmth Perception	1	634	1.014	0.314
Competence Perception	1	634	9.688	0.002
Modern Racism	1	634	10.737	0.001

UK Sample: Ventilator

	**Numerator df**	**Denominator df**	**F**	**Sig.**
Intercept	1	624	1.419	0.234
Scenario Type (Group or Patient)	1	624	1.926	0.166
Age	1	624	1.316	0.252
Race (UK)	3	624	0.864	0.46
Gender	7	624	1.241	0.278
Highest Schooling	1	624	0.066	0.797
Social Status	1	624	0.913	0.34
Household Income	1	624	0.096	0.757
Political Orientation (General)	1	624	0.038	0.846
Political Orientation (Economical)	1	624	0.59	0.443
Political Orientation (Social)	4	624	4.889	0.001
Injustice Perception	1	624	2.732	0.099
Responsibility Perception	1	624	0.744	0.389
Warmth Perception	1	624	3.129	0.077
Competence Perception	1	624	4.65	0.031
Modern Racism	1	624	13.881	< .0001

6. Perceptions of Injustice, Responsibility, and Stereotypes

In the US and the UK, participants were more likely to believe that adverse COVID-19 related health outcomes were the result of injustice and outside the individual's control for race than for obesity/male sex [Figure S2]. In particular, injustice was perceived differently for the three additional risk

Kappes A, *et al. BMJ Open* 2022; 12:e062561. doi: 10.1136/bmjopen-2022-062561

factors (F(2, 2577)=217.388, P <0.0001, partial eta^2 =0.144). Bonferroni-corrected posthoc comparisons indicated that participants most strongly attributed the increased numbers of deaths of racial minorities due to injustice, stronger than the deaths of men (P < .0001, 95% CI: 0.372-0.469), and obese people (P < 0.000, 95% CI: 0.300-0.399). For the latter two, participants indicate more injustice to obese people death's than to men (P < .0001, 95% CI: 0.035-0.108). Similarly, there were differences in perceived personal control for adverse outcomes from COVID (F(2, 2577)=284.123, P <.0001, partial eta^2 = .181). Racial minorities were perceived to have the least control over the higher death rate compared to men (P =0.009, CI 95: 0.011-0.103) and obese people (P < .0001, 95% CI:0.529-0.655). Obese people were perceived to have most control over the higher death rates attributable to COVID, higher control than men (P < .0001, 95% CI: 0.595-0.476) and people from racial minorities (P =0.009, 95% CI: 0.011-0.103).

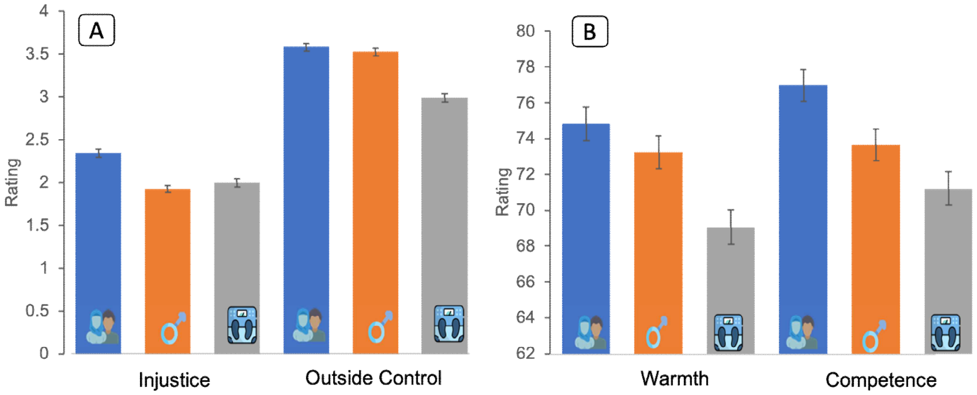

Supplementary Figure 2: Perceptions of Injustice, Control, Warmth, and Competence. A: Bonferroni-corrected comparisons indicated that participants most strongly attributed the increased numbers of deaths of racial minorities to injustice, stronger than the deaths of males (P < .0001, 95% CI: 0.372-0.469), and obese people (P < 0.000, 95% CI: 0.300-0.399). Participants attributed slightly more injustice to obese people's deaths than to males' deaths (P < .0001, 95% CI: 0.035-0.108). Similarly, racial minorities were perceived to have less control over the higher death rate compared to males (P =0.009, CI 95: 0.011-0.103) and obese people (P < .0001, 95% CI:0.529-0.655). Obese people were perceived to have the most control over higher death rates attributable to COVID-19, more than males (P < .0001, 95% CI: 0.595-0.476) and racial minorities (P =0.009, 95% CI: 0.011-0.103). B: Survey participants had the strongest stereotype content feelings of warmth towards ethical minorities, compared to attitudes towards males (p =0.005, Bonferroni corrected, small difference) and obese people (P < 0.0001, Bonferroni corrected). Participants also expressed higher feelings of competence toward individuals from a racial minority than males (P < 0.0001, Bonferroni corrected), or obese people (P < 0.0001, Bonferroni corrected). (Error bars = 95% Confidence Interval).

How did these perceptions of injustice and responsibility differ between the US and the UK? For injustice, we found no main effect of country (F(1,2576) = 2.534, P =.112, partial eta^2 =.001) and no interaction effect with categories of disadvantage (F(1,2576) = 1.622, P =0.203, partial eta^2 =.001). Participants in both countries perceived the relative injustice that affected each group similarly. When examining responsibility, we found no main effect of country (F(1,2576) = 0.366, P =0.545, partial eta^2 = 0.00001), but an interaction between country and category (F(1,2576) =31.651, P < .0001, partial eta^2 = 0.012). Participants in the US gave men less responsibility (t(2578) = 4.091, P < 0.0001), but obese people more responsibility (t(2578) = 4.176 .000, P < 0.0001) than participants in the UK.

Perceptions of injustice and responsibility might be related to the general attitudes towards a particular category. Attitudes can be characterized via two different dimensions, warmth and competence[56,57]**.** We found that again, respondents had different feelings of warmth towards different

categories (F(1, 2577) = 72.446, P <0.0001, partial eta^2 = .029) and competence (F(1, 2577) = 76.369, P < 0.0001, partial eta^2 =0.030). In particular, survey participants had strongest feelings of warmth towards ethical minorities, compared to feelings towards men (p =0.005, Bonferroni corrected, small difference) and obese people (P < 0.0001, Bonferroni corrected), and rated the warmth of obese people lower than the warmth of men (P < 0.0001, Bonferroni corrected). When examining competence, we found a similar effect of group membership (F(2,2575) = 77.00, P < 0.0001, partial eta^2 = .03). Participants expressed higher feelings of competence in individuals from a racial minority than men (P < 0.0001, Bonferroni corrected), or obese people (P < 0.0001, Bonferroni corrected), while men were perceived as more competent than obese people (P < 0.0001, Bonferroni corrected).

Again, one might wonder whether participants from the US and the UK had different stereotypes about racial minorities, men, and obese people. When examining the effect of country on warmth, we find a main effect of country, F(1,2497) = 19.408, P < 0.0001, partial eta^2 = .008, American participants gave higher warmth ratings overall, though this varied between categories, F(2,2497) = 15.083, P < .0001, partial eta^2 = 0.006. The biggest difference was in warmth perception of obese participants, with American participants rating obese people more warmly than British participants (t(2495) = 5.73, P < .001), and only very small differences for the other two categories between both countries. When examining the effect of country and competence ratings, a similar picture emerged. We found a main effect for country (F(2,2497) = 25.16, P <.0001 , partial eta^2 = .01, which was qualified by an interaction effect with disadvantage categories, F(2,2497) = 17.49, P <001 , partial eta^2 = .007). Again, the main difference was for perceptions of obese people, Americans perceived obese people as more competent than British people, t(2497) = 6.672, P < .0001).

Why do people perceive the effect of a specific additional risk factor such as race as unjust? Part of the reason might be because of how favorably one thinks about the additional risk factor in general. To test this idea, we regressed feelings of responsibility, warmth, and competence on perceptions of injustice for each of the three additional risk factors. For race, we found that perceptions of responsibility (beta = .130, t(2482) = 6.551, P <.0001) and feelings of warmth, (beta = .209, t(2482) = 6.89, P < .0001) predicted the perception of injustice, but not perceptions of competence (beta = -.039, t(2482) = 1.309, P =0.191). For men, neither responsibility (beta = .032, t(2482) = 1.589,P = 0.112), competence (beta-.054, t-1.930 P = 0.054), or warmth (beta = .035, t1.262, P = 0.207) predicted perceptions of injustice. In contrast, for obesity, responsibility (beta = .222, t(2482) = 11.047, P < 0.0001), warmth (beta = .103, t(2482) = 3.587, P < 0.0001), and competence (beta = -.074, t(2482) =2.624, P = 0.009) predicted perceptions of injustice.

4. Allocation attitudes for participants with low and high modern racism scores

In the US and the UK, responses on the Modern Racism Scale were associated with attitudes towards ventilator and vaccine allocation based on race (Ventilator: US: F(1,634) = 10.73, p = 0.001, UK: F(1,624) = 13.88, P < .0001; Vaccine: US: F(1,621 =23.00, P <0.0001, UK: F(1,607)=16.98, P <0.0001). The more participants endorsed modern racism statements, the less positive their composite attitudes towards ventilator or vaccine allocation to racial minority patients. A minority (27.3%) of participants had a mean score of higher than three on the modern racism scale, indicating an endorsement of modern racism. These participants did not give weight to race in ventilator or vaccine allocations [SM, Figure 3]. Participants with low modern racism scores also did not give weight to race in ventilator allocation but did for vaccines. Modern racism did not affect prioritisation based on sex/obesity [SM Figure 3]. Perceptions of injustice did not predict ventilator or vaccine allocation attitudes in the US (F(1,634) = 0.778, P = 0.378) or the UK (F(1,624) = 2.732, P = 0.099).

Kappes A, *et al. BMJ Open* 2022; 12:e062561. doi: 10.1136/bmjopen-2022-062561

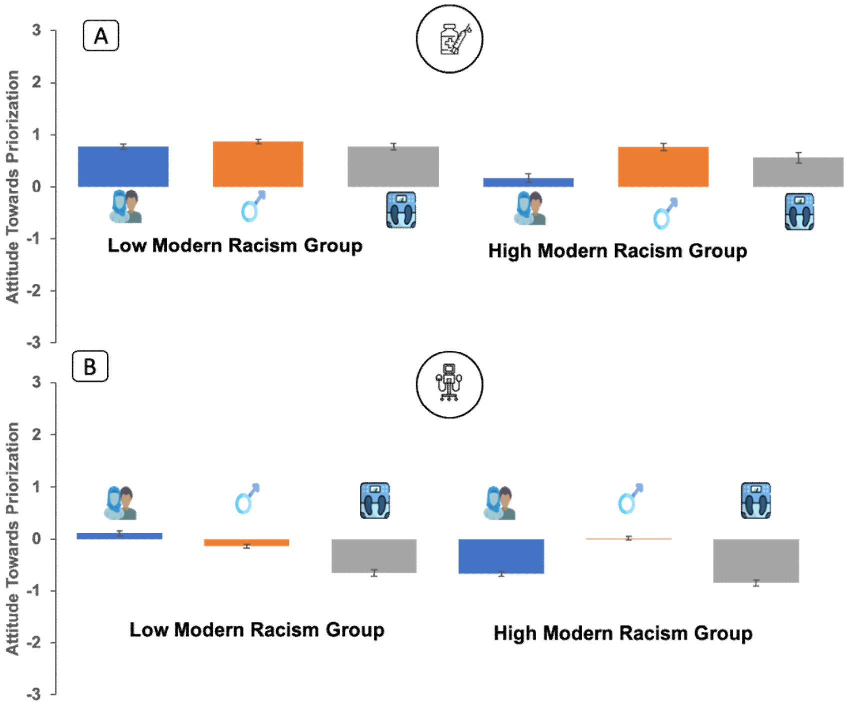

Supplementary Figure 3: Allocation attitudes for participants with low modern racism scores (Low Modern Racism Group) and high modern racism scores (High Modern Racism Group). A: For vaccines, participants in the Low Modern Racism Group (mean score lower than scale midpoint, N = 1011) had a more positive attitude towards race-based vaccine allocation compared to participants in the High Modern Racism Group (mean score higher than scale midpoint, N = 307). Allocation attitudes for sex and obesity were similar. B: For ventilators, we find that participants in the Low Modern Racism Group (N = 919) had a neutral attitude towards race-based vaccine allocation, while participants in the High Modern Racism Group (N = 343) had a negative one. Allocation attitudes for sex and obesity were similar.

References (Supplemental Materials)

1. J. B. McConahay, “Modern racism, ambivalence, and the Modern Racism Scale” in *Prejudice, Discrimination, and Racism*, J. F. Dovidio, S. L. Gaertner, Eds. (Academic Press, 1986), pp. 91–125.

2. T. G. Morrison, M. Kiss, Modern racism scale. *Encyclopedia of personality and individual differences*, 1–3 (2017).

3. , U.S. Census Bureau QuickFacts: United States (July 22, 2021).

4. D. Wilkinson, H. Zohny, A. Kappes, W. Sinnott-Armstrong, J. Savulescu, Which factors should be included in triage? An online survey of the attitudes of the UK general public to pandemic triage dilemmas. *BMJ Open* **10**, e045593 (2020).

5. , Why are more people from BAME backgrounds dying from coronavirus? *BBC News* (2020) (June 28, 2021).

6. T. Takahashi, *et al.*, Sex differences in immune responses that underlie COVID-19 disease outcomes. *Nature* **588**, 315–320 (2020).

7. J. Yang, J. Hu, C. Zhu, Obesity aggravates COVID-19: a systematic review and meta-analysis. *Journal of medical virology* **93**, 257–261 (2021).

8. P. Slovic, "If i look at the mass i will never act: Psychic numbingpsychic numbing and genocidegenocide" in *Emotions and Risky Technologies*, (Springer, 2010), pp. 37–59.

9. S. T. Fiske, A. J. Cuddy, P. Glick, J. Xu, "A model of (often mixed) stereotype content: Competence and warmth respectively follow from perceived status and competition (2002)" in *Social Cognition*, (Routledge, 2018), pp. 171–222.

10. S. T. Fiske, A. J. Cuddy, P. Glick, Universal dimensions of social cognition: Warmth and competence. *Trends in cognitive sciences* **11**, 77–83 (2007).

11. C. W. Blatz, M. Ross, Principled ideology or racism: Why do modern racists oppose race-based social justice programs? *Journal of Experimental Social Psychology* **45**, 258–261 (2009).
