# Supplementary material for: Race and resource allocation: an online survey of US and UK adults’ attitudes toward COVID-19 ventilator and vaccine distribution
Source: BMJ Open. 2022 Nov 21;12(11):e062561. doi: 10.1136/bmjopen-2022-062561 (PMC9679868; doi:10.1136/bmjopen-2022-062561)
Supplement: Author's manuscript [file bmjopen-2022-062561.draft_revisions.pdf]

BMJ Open

BMJ Open is committed to open peer review. As part of this commitment we make the peer review history of every article we publish publicly available.

When an article is published we post the peer reviewers' comments and the authors' responses online. We also post the versions of the paper that were used during peer review. These are the versions that the peer review comments apply to.

The versions of the paper that follow are the versions that were submitted during the peer review process. They are not the versions of record or the final published versions. They should not be cited or distributed as the published version of this manuscript.

BMJ Open is an open access journal and the full, final, typeset and author-corrected version of record of the manuscript is available on our site with no access controls, subscription charges or pay-per-view fees (http://bmjopen.bmj.com).

If you have any questions on BMJ Open's open peer review process please email info.bmjopen@bmj.com

BMJ Open

Public attitudes towards race and resource allocation in a pandemic

Journal:	*BMJ Open*
Manuscript ID	bmjopen-2022-062561
Article Type:	Original research
Date Submitted by the Author:	11-Mar-2022

Complete List of Authors:	Kappes, Andreas; City University of London<br>Zohny, Hazem; University of Oxford,<br>Savulescu, Julian; University of Oxford, MEDICAL ASSESSMENT UNIT<br>Singh, Ilina; University of Oxford, Psychiatry<br>Sinnott-Armstrong, Walter; Duke University<br>Wilkinson, Dominic; University of Oxford, Oxford Uehiro Centre for Practical Ethics; John Radcliffe Hospital,

Keywords:	COVID-19, Health policy < HEALTH SERVICES ADMINISTRATION & MANAGEMENT, MEDICAL ETHICS

SCHOLARONE™
Manuscripts

I, the Submitting Author has the right to grant and does grant on behalf of all authors of the Work (as defined in the below author licence), an exclusive licence and/or a non-exclusive licence for contributions from authors who are: i) UK Crown employees; ii) where BMJ has agreed a CC-BY licence shall apply, and/or iii) in accordance with the terms applicable for US Federal Government officers or employees acting as part of their official duties; on a worldwide, perpetual, irrevocable, royalty-free basis to BMJ Publishing Group Ltd ("BMJ") its licensees and where the relevant Journal is co-owned by BMJ to the co-owners of the Journal, to publish the Work in this journal and any other BMJ products and to exploit all rights, as set out in our licence.

The Submitting Author accepts and understands that any supply made under these terms is made by BMJ to the Submitting Author unless you are acting as an employee on behalf of your employer or a postgraduate student of an affiliated institution which is paying any applicable article publishing charge ("APC") for Open Access articles. Where the Submitting Author wishes to make the Work available on an Open Access basis (and intends to pay the relevant APC), the terms of reuse of such Open Access shall be governed by a Creative Commons licence – details of these licences and which Creative Commons licence will apply to this Work are set out in our licence referred to above.

Other than as permitted in any relevant BMJ Author's Self Archiving Policies, I confirm this Work has not been accepted for publication elsewhere, is not being considered for publication elsewhere and does not duplicate material already published. I confirm all authors consent to publication of this Work and authorise the granting of this licence.

For peer review only

Public attitudes towards race and resource allocation in a pandemic

Andreas Kappes PhD[1], Hazem Zohny PhD[2], Julian Savulescu PhD[2,3], Ilina Singh PhD[4,5], Walter Sinnott-Armstrong PhD[6], Dominic Wilkinson DPhil[2,3,7]

Affiliations:
[1]City, University of London
[2] Oxford Uehiro Centre for Practical Ethics, University of Oxford
[3] Murdoch Children's Research Institute, Melbourne, Australia.
[4] Wellcome Trust Centre for Ethics and the Humanities, University of Oxford
[5] Department of Psychiatry, University of Oxford
[6] Kenan Institute for Ethics and Philosophy Department, Duke University
[7] John Radcliffe Hospital, Oxford, UK

Correspondence: Dr Hazem Zohny, Oxford Uehiro Centre for Practical Ethics, Suite 8, Littlegate House, St Ebbes St, Oxford, OX1 1PT, UK. Tel: +44 1865 286888, Email: hazem.zohny@philosophy.ox.ac.uk

For peer review only

For peer review only - http://bmjopen.bmj.com/site/about/guidelines.xhtml

Abstract

Objective: This study aimed to assess US and UK adults' attitudes towards COVID-19 ventilator and vaccine allocation.

Design: Online survey including US and UK adults, sampled to be representative for sex, age, race, household income, and employment. A total of 2580 participated [female = 1289, Age Range = 18 to 85].

Interventions: Participants were asked to allocate ventilators or vaccines in scenarios involving individuals or groups with different medical risk and additional risk factors.

Results: When asked to allocate ventilators (vaccines) to patients with identical predicted chances of survival (risk of severe COVID-19) but who were either from a racial minority or were white, 14.8% (43.6%) of participants allocated to racial minority patients, 68.9% (49.2%) chose to toss a coin, and 16.2% (7.2%) chose the white patient. When the racial minority patient had a 10% lower chance of survival (or 10% lower chance of severe COVID-19), 12.4% (23.7%) participants allocated the ventilator (vaccine) to the racial minority patient. Similar results were seen for obesity or male sex as additional risk factors. Participant race did not impact vaccine or ventilator allocation decisions in the US, but did impact ventilator allocation attitudes in the UK ($F(4,602) = 6.95$, $P < 0.001$). In both countries, responses on the Modern Racism Scale were strongly associated with attitudes towards race-based ventilator and vaccine allocations ($P < 0.0001$).

Conclusions: Although living in countries with high racial inequality during a pandemic, most US and UK adults allocated ventilators and vaccines preferentially to those with the highest chance of survival or highest chance of severe illness. Few participants gave additional weight to race for ventilator allocation, but race of recipient led to vaccine prioritisation in cases where risk of illness was similar.

For peer review only

Introduction

The coronavirus pandemic has raised contentious ethical questions about the distribution of scarce healthcare resources during a crisis. For example, early concern about the supply of intensive care beds and mechanical ventilators led healthcare systems to develop triage criteria in the event of a shortage.[1,2] Later, states had to decide whom to prioritise for access to the limited supply of vaccines.[3,4] Although different countries have followed different strategies, a prevailing response to these questions has focused on saving the most lives.[3,5–8] For ventilator triage, this entailed giving priority to those with the highest chance of survival.[9] For vaccines, those with the highest risk of severe illness were prioritised.[10]

However, this has been criticised[11] for ignoring evidence that individuals from racial minorities have been disproportionately affected by the pandemic.[12–14] In the US, Black, Hispanic and Indigenous people were more likely to be infected, hospitalised and die of COVID-19 than White individuals.[15] Similarly, high morbidity and mortality rates were seen in Black, Asian and Minority Ethnic groups (BAME) in the UK.[16] This raises an important ethical question: should individuals from disproportionately affected racial minorities be prioritised when allocating scarce resources such as ventilators and vaccines? If so, how should this be weighed against other ethical values in resource allocation, including the desire to save the most lives and the need to treat patients equally?

Some legal scholars, policy advocates, and ethicists have suggested that race should be factored into ventilator and vaccine allocation.[17–19] For example, some have proposed positive discrimination in the form of equity weights.[12] However, such an approach might raise political[20] or legal concerns.[21] Alternatively, it could lead to ethical questions. Treating members of racial groups differently would conflict with principles of equality. Furthermore, in some circumstances, preferential allocation to members of a disadvantaged group could increase overall mortality from COVID-19[22,23]. On the other hand, not considering it could widen the race-based difference in COVID-19 deaths.

Policy discussion during the pandemic has largely lacked information on public opinion: it is not known what people in countries like the US and UK, with high racial inequality in COVID-19 deaths, think about these questions. There is some evidence that the public support including race in allocation of vaccines,[24] but none about the *relative* importance of medical versus racial factors.

We sought to examine the relative weight given by the public to race in allocation and to compare this with participants' views on obesity and male sex. Race, obesity, and sex are independent risk factors for COVID-19 (in one UK study, hazard ratios for COVID-19 death were 1.48, 1.40, and 1.59, respectively[25]), but they differ ethically (for example, in their association with historical injustice). Finally, we sought to explore whether prioritisation preferences differed between racial groups, socio-economic status, or political orientations or were related to stereotypes and covert racial attitudes. This would illuminate what drives prioritisation attitudes but also whether a broader consensus exists.

For peer review only - http://bmjopen.bmj.com/site/about/guidelines.xhtml

Methods

The study was approved by the University of Oxford Central University Research Ethics Committee [R73841/RE002]. It follows the American Association for Public Opinion Research (AAPOR) reporting guideline.

Participants were recruited from large market research panels and paid $10.8/hour. Speed and attention checks were used to identify and exclude respondents not paying sufficient attention to question details.

Two surveys were conducted in December 2020/January 2021, (US n=1296, UK n=1284). (Table 1). Each sub-sample size gave the ability to estimate true preferences with a 4% margin of error (95% confidence interval) for the US and the UK population. The survey achieved a completion rate (completed surveys divided by number of respondents who entered the survey) of 86.7%. As the number of people that had the chance to participate in the opt-in panel is not known, we cannot report the AAPOR RR1[9].

Table 1: Demographic Details of Participants. Participants were sampled to be representatives of the US and UK general population for sex, age, race, household income, and employment. (Different income categories were used to reflect known information on population characteristics). Fourteen percent of the UK sample, and 19% of the US sample described their racial background as non-White. Note that, compared to the last census[26], the US sample had a lower proportion of Hispanic and Black participants than the general US population.

	**US / UK**	**US Sample**	**UK Sample**
		n (%)	n (%)
**Gender**	Male	634 (48.9%)	630 (49.1%)
	Female	656 (50.6%)	650 (50.6%
	Other	3 (0.2%)	3 (0.2%)
	Prefer not to say	3 (0.2%)	1 (0.1%)
**Race**	White	1034 (79.8%)	1103 (85.9%)
	Hispanic or Latino	70 (5.4%)	-
	Black or African American / Black or Black British	114 (8.8%)	41 (3.2%)
	Native American or American Indian	19 (1.5%)	-
	Mixed	-	29 (2.3%)
	Asian or Pacific Islander / Asian or Asian British	23 (1.8%)	98 (7.6%)
	Other	21 (1.6%)	11 (0.9%)
	Prefer not to say	11 (0.8%)	2 (0.2%)
**Age**	18 - 24	159 (15.5%)	145 (11.3%))
	25 - 34	248 (18.9%)	303 *23.6%)
	35 - 44	237 (18.9%)	291 (*22.7%)
	45 - 54	252 (18.3%)	238 (18.5%)
	55 - 64	224 (14.2%)	198 (15.4%)
	65 - 74	129 (5.9%)	89 (6.9%)
	75 - 84	38 (1.6%)	20 *1.6%)
	85 or older	4 (0.6%)	-
**Highest level of schooling**	Less than high school degree / Primary school	24 (1.9%)	3 (0.2%)
	High school graduate or equivalent / Secondary school up to 16 years	237 (18.3%)	236 (18.4%)
	Some college but no degree / Higher or secondary or further education	250 (19.3%)	441 (34.3%))
	Associate degree in college (2-year)	122 (9.4%)	-
	Bachelor's degree	160 (12.3%)	381 29.7%)
	Master's degree	290 (22.4%)	138 (10.7%)

For peer review only

For peer review only - http://bmjopen.bmj.com/site/about/guidelines.xhtml

	Doctoral degree	29 (2.2%)	29 (2.3%)
	Professional degree (JD, MD)	38 (2.9%)	47 (3.7%)
	Prefer not to say	6 (0.5%)	9 (0.7%)
	Other	140 (10.8%)	-
**Employment status**	Employed part time	136 (10.5%)	735 (57.2%)
	Employed full time	617 (47.6%)	232 (18.1%)
	Unemployed looking for work	96 (7.4%)	70 (5.5%)
	Unemployed not looking for work	113 (8.7%)	96 (7.5%)
	Retired	274 (21.1%)	114 (8.9%)
	Student	48 (3.7%)	37 (2.9%)
	Disabled	12 (0.9%)	-
**Estimated Household Income**	$0-$25k / £0-£20k	230 (17%)	263 (20.5%)
	$25k-$50k / £20k-£30k	274 (21.1%)	276 (21.5%)
	£30k-£40k	-	198 (15.4%)
	£40k-£50k	-	145 (11.3%)
	$50k-$75k / £50k-£60k	224 (17.3%)	106 (8.3%)
	$75k-$100k / £60k-£100k	168 (13%)	191 (14.9%)
	$100k-$150k / £100k+	199 (15.4%)	105 (8.2%)
	$150k+	103 (7.9%)	-
	Prefer not to say	11 (0.8%)	-

The surveys were adapted from a previous study.[27] Participants imagined that they needed to make decisions in the setting of a severe shortage of ventilators or vaccines. To examine how much weight participants would give to predicted chance (based on age and comorbidity) of survival for allocating ventilators, or of developing severe COVID-19 for vaccines, participants read scenarios where potential recipients differed in those variables to different degrees. For example, in one ventilator scenario, one patient had a 60% chance of survival, the other a 40% chance. In a corresponding vaccine scenario, participants had a 20% vs 10% chance of developing severe COVID-19. Participants decided whom to give the ventilator or vaccine to or to toss a coin (Figure 1). Thereafter, participants were randomly introduced to one of the three additional factors: race, sex, and obesity.

For a racial minority in the US, we chose ‘Black recipients’ and, in the UK, ‘BAME recipients’. BAME is a common term in the United Kingdom, used to describe non-white racial groups.[28] Before completing scenarios, we reminded participants of the racial inequality in COVID-19-related deaths in the UK and in the US. Thereafter, participants worked on five scenarios involving one Black (BAME) and one White patient. In one scenario, both patients had the same chance of surviving/severe COVID-19; in two scenarios the Black (BAME) patient had a higher chance of survival/severe COVID-19; in two scenarios the White patient had a higher chance. Participants indicated for each whether they wanted to give the ventilator/vaccine to the patient from a racial minority, the white patient, or toss a coin.

Participants saw a similar set of scenarios for sex and obesity as additional risk factors [SM]. To test whether it would make a difference, we repeated the scenarios for groups of patients.

We asked participants questions to capture their perceptions of racial minorities, men, and obese people, including their views about the degree to which worse outcomes from COVID-19 were a result of social injustice or under the control of the individual. We also administered the modern racism scale, intended to capture subtle or covert discriminatory attitudes.[29]

For peer review only - http://bmjopen.bmj.com/site/about/guidelines.xhtml

Statistical analysis

In parallel scenarios, results are presented as ventilators (vaccines). To examine the differences between risk factors, and the variables that impacted participants' attitudes, we created a composite attitude score for each set of scenarios by counting the number of times participants decided to give the ventilator or vaccine to the person or group with an additional risk factor in successive scenarios. To test whether differences existed between risk factors, we used repeated measure analysis of variance with Bonferroni corrected post-hoc comparisons. To test which variables impacted participants' attitudes towards risk factors, we ran a series of four linear models to explore the role of various factors in predicting racial prioritisation attitudes. For each model, we defined attitudes as dependent variable and subjects as the random factor. In the first two models, we entered age, gender, race, relative income, educational level, political ideology (general, social, and economical), and type of scenario (patient versus group-based) as fixed factors. In the second two models, we additionally added perceptions of injustice, responsibility, warmth, competence, and modern racism scores. The full results of each model can be seen in the Supplemental Results (Supplementary Table 1). We used SPSS 28 for all analyses.

Patient and public involvement

No patient involvement.

Results

Prioritisation of medical risk

We first assessed the impact of medical risk factors on allocation. Asked to choose between patients 50% vs 40% chance of surviving if treated, 71.9% (95% CI: 69.3%-74.3%,) of participants gave the ventilator to the patient with higher chances. For vaccines (15% vs 10% chance of severe COVID-19), 70.8% (95% CI: 68.3%-73.2%) gave the vaccine to the higher risk recipient. This increased when the difference between patients increased (Figure 2). When there was no difference, 82.7 (86%) (95% CI: 80.5-84.8,84.6-88.4) tossed a coin. Patterns of response were very similar for scenarios involving groups as for individual patients (Figure 2).

Risk factors and resource allocation

In scenarios involving patients with identical chances of survival (severe illness) but different race, 14.8 (43.6%) of participants allocated the ventilator (vaccine) to Black or BAME patients, 68.9 (49.2%) chose to toss a coin, and 16.2 (7.2%) chose the White patient. When the racial minority patient had a lower chance of survival (severe illness), <15% of respondents allocated the ventilator or vaccine to them (Figure 3).

When chances of survival were equal, but one patient was male and the other female, 11.9% (95% CI: 10.2%,13.8%) of participants gave the ventilator to the male, (22.7% female patient; 65.4% tossed a coin). When obesity was an additional risk factor, 11.9% (95% CI: 11.6% - 15.5%) chose the obese patient, (41.1% healthy weight, 45.4% tossed a coin).

For peer review only - http://bmjopen.bmj.com/site/about/guidelines.xhtml

For vaccines, in scenarios of equal medical risk, where one patient was male and the other female, 44.2% of participants gave the vaccine to the male patient. When obesity was an additional risk factor, 59.2% allocated to an obese patient (Figure 3).

Using the composite attitude score, race and sex did not affect allocation decisions for ventilators, but obesity did so negatively: ($P < .001$, 95% CI -.9485, -.7124). Participants had lower prioritisation preferences for obese patients, (compared with racial minority, $P < 0.001$, and male patients, $P = .030$). For vaccines, participants had significant prioritisation preference for race, sex, and obesity (attitude greater than zero: $p < .00001$; post-hoc analyses: obese patients > racial minorities, $P < 0.001$, and male patients, $P < 0.001$.) There was a very small main effect for scenario type (group preference – Supplementary Figure 1). Country of participants (US vs UK) did not alter allocation attitudes ($F(1,1260) = 0.619$, $P = 0.431$).

Determinants of prioritisation

Using mixed linear models [Supplementary Table 1, full results], we find that race of participant impacted ventilator allocation attitudes in the UK ($F(4,602) = 6.95$, $P < 0.001$) but not the US ($F(6, 635) = 1.38$, $P = 0.21$). In the UK, Asian and Black participants had more positive attitudes towards race in allocation of ventilators than white participants [SM]. Among BAME participants, 22.1 % of participants gave the ventilator to a BAME patient with a 10% lower chance of survival, while only 6.9% of the White participants did. Age, gender, socio-economic status, education, and political ideology did not impact ventilator allocation attitudes. Those participants that strongly endorsed ventilator allocations based on chances of survival in the scenarios without any additional risk factors were not more or less likely to reject ventilator allocation based on race ($r = -0.047$).

Race of participants did not affect vaccine allocation. In the US, (but not the UK) gender (F3,661) = 7.238, $P < .0001$) and social political orientation F(1,661) =11.388, $P = 0.001$) impacted vaccine allocation based on race. Male participants had a less positive attitude ($P = 0.003$) and more socially conservative participants gave less weight to race in vaccine allocation (parameter estimate = - 0.305, 95% CI = -.480, -.130).

Influence of attitudes towards race on prioritisation

In both countries, participants were more likely to believe that adverse COVID-19 related health outcomes were the result of injustice and outside the individual's control for race than for obesity/male sex [Supplementary Figure 2]. Obesity was associated with the strongest sense of individuals' health outcomes being within their control. US and UK respondents had similar attitudes towards the respective racial minorities [SM].

In the US and the UK, responses on the Modern Racism Scale were associated with attitudes towards ventilator and vaccine allocation based on race (Ventilator: US: $F(1,634) = 10.73$, $p = 0.001$, UK: $F(1,624) = 13.88$, $P < .0001$; Vaccine: US: F(1,621 =23.00, $P < 0.0001$, UK: $F(1,607)=16.98$, $P < 0.0001$). The more participants endorsed modern racism statements, the less positive their composite attitudes towards ventilator or vaccine allocation to racial minority patients. A minority (27.3%) of participants had a mean score of higher than three on the modern racism scale, indicating an endorsement of modern racism. These participants did not give weight to race in ventilator or vaccine allocations [Supplementary Figure 3].

For peer review only - http://bmjopen.bmj.com/site/about/guidelines.xhtml

Participants with low modern racism scores also did not give weight to race in ventilator allocation but did for vaccines. Modern racism did not affect prioritisation based on sex/obesity [Supplementary Figure 3]. Perceptions of injustice did not predict ventilator or vaccine allocation attitudes in the US ($F(1,634) = 0.778$, $P = 0.378$) or the UK ($F(1,624) = 2.732$, $P = 0.099$).

Discussion

Previous research has identified some of the public's prioritisation preferences in relation to race and vaccines.[24] To our knowledge, this is the first study to examine the views of the public on race in ventilator allocation and in comparison with other factors. This international study was conducted during the second wave of the COVID-19 pandemic in two countries with documented and publicised adverse outcomes for racial minorities. Survey participants largely allocated ventilators and vaccines on the basis of medical factors and independent of the race of recipients. Few respondents in our survey, even those from racial minorities themselves, endorsed priority for ventilators for those from racial minorities. However, where other factors were equal, 40-60% of respondents allocated vaccines to patients/groups with additional risk based on race, sex, or obesity. In the survey, willingness to prioritise based on additional risk did not correlate with participants' perceptions of whether risk factors were caused by social injustice. However, covert racial attitudes (endorsed by approximately a quarter of respondents) negatively impacted attitudes to allocation in both countries.

The different weight given to race in the allocation of vaccines versus ventilators in our study may reflect people's desire to prevent the most deaths.[7,30] Previous research has shown strong public endorsement for prioritising chances of survival in ventilator allocation and probability of severe illness for vaccine allocation.[24,27,31–33] However, whereas vaccines are provided to prevent illness, ventilators are provided to those already critically ill. Participants might have felt that allocating ventilators preferentially to those with additional risk factors (race, sex, obesity) could lead to more deaths from COVID-19 if such patients (even with otherwise equal medical risk) have higher mortality rates.

However, in our study participants who expressed a strong attitude for ventilator allocation based on chances of survival were not more or less likely to reject preference in ventilator allocation based on race. Other ethical principles or considerations may be guiding allocation decisions, for example concern for equality and avoiding discrimination.[34,35] Respondents did not believe that racial minorities should be at a disadvantage for access to ventilators – reinforcing the importance of ensuring that medical criteria used for allocation are not racially biased.[36,37]

One reason to potentially give priority in allocation of scarce treatment to racial minorities is because of concerns over past or present injustice.[12] In our survey, UK and US respondents perceived higher death rates among minorities to be more unjust and less under individual control than the mortality rates of male and obese recipients. However, these perceptions did not affect allocation decisions. Similarly, political orientation had little impact on allocation decisions in our survey. More conservative respondents were less inclined to indicate that injustice is responsible for higher death rates. However, this perception did not affect allocation decisions.

For peer review only - http://bmjopen.bmj.com/site/about/guidelines.xhtml

Allocation decisions in our survey were associated with responses on the modern racism scale. This instrument is intended to capture covert racial attitudes.[29] While people who score high on the modern racism scale potentially do not perceive themselves as racist, discriminatory attitudes can nevertheless guide their decisions. Modern racism might measure negative attitude towards affirmative action policies in general. However, in our survey, modern racism did not predict attitudes to sex or obesity in allocation. Furthermore, in our models, political ideology was not associated with allocation based on race. Taken together, these findings suggest that some opposition to giving weight to race in prioritisation arises from covert racial attitudes. However, even those respondents with low scores on the modern racism scale (three quarters of those surveyed) did not on average give weight to race in allocation of ventilators. Covert racial attitudes do not appear to explain our overall finding of the lack of weight given to race in public attitudes to ventilator allocation.

Importantly our results help contextualise previous findings that looked at race in allocation in isolation. For example, a US Gallup/COVID Collaborative survey found that 74-85% respondents supported giving priority for vaccine access to Black, Hispanic and Native Americans.[24] In our study, slightly less than half of respondents endorsed this, but only in cases where other risk factors were equal. Our findings mirror recent ones about affirmative action in higher education, where Americans have positive attitudes towards affirmative action in general, but also feel strongly that race should not trump other admission criteria such as grades.[39] This highlights the importance of clarifying the weight given to different ethical values when surveying the public's views on resource allocation, but also points to the importance of alternative approaches to lessening the impact of Covid-19 on racial minorities.

Conclusion

Obviously, the results of this survey do not settle the ethical question of whether governments should prioritise based on race or other forms of social disadvantage. Ethical argument and analysis may lead to prioritisation policies that differ from those endorsed by the majority. However, understanding the general approach of the public towards prioritisation in settings of severe scarcity may be important in debate and deliberation: it may challenge or confirm assumptions about what the public would or would not support, as well as highlight areas of ethical consensus or point to important areas of disagreement.[40] Our survey identified a broad consensus, in both the US and UK, for how scarce resources should be allocated during a pandemic. One key limitation here is that in our US sample, Hispanic and Black participants were underrepresented. Thus, our study may underestimate how different racial groups feel about allocation of scarce resources. However, we did find support from a large minority for inclusion of race as a tie-break consideration in vaccine allocation. We also found that the vast majority of participants (including those from minority groups) think that in situations of extreme scarcity, life-saving treatment like ventilators should be allocated in a race-neutral way, and preferentially to those with the highest chances of survival. Although we may hope that it is never needed, that finding may be relevant to planning for the next pandemic.

Contribution statement: AK, HZ, and DW designed the survey, JS, IS, and WS-A provided crucial feedback on methodology and design. HZ organised the collection of the data. AK analyzed the data. AK, HZ, and DW wrote the manuscript, JS, IS, and WS-A provided crucial feedback.

For peer review only - http://bmjopen.bmj.com/site/about/guidelines.xhtml

Competing interests: The authors declare that they have no conflicts of interest.

Ethics approval: The study was approved by the University of Oxford Central University Research Ethics Committee [R73841/RE002].

Funding:
This study (survey costs) was commissioned and paid for by the World Health Organization (WHO). Copyright in the original work on which this article is based belongs to WHO. The authors have been given permission to publish this article.

Researchers were funded in part, by the Wellcome Trust [203132/Z/16/Z] (DW, HZ) and by the Arts and Humanities Research Council (AHRC) (DW) as part of the UK Research and Innovation rapid response to COVID-19 [AH/V013947/1], as well as the NIHR Oxford Health Biomedical Research Centre (IS-BRC-1215-20005) (IS). The funders had no role in the preparation of this manuscript or the decision to submit for publication. For the purpose of open access, the authors have applied a CC BY public copyright license to any Author Accepted Manuscript version arising from this submission.

Data sharing statement: All data, code, and materials used in the analysis can be found at: osf.io/mhyqz

For peer review only

For peer review only - http://bmjopen.bmj.com/site/about/guidelines.xhtml

References

1. Wilkinson D. ICU triage in an impending crisis: uncertainty, pre-emption and preparation. *J Med Ethics*. 2020;46(5):287-288. doi:10.1136/medethics-2020-106226

2. Verelst F, Kuylen E, Beutels P. Indications for healthcare surge capacity in European countries facing an exponential increase in coronavirus disease (COVID-19) cases, March 2020. *Eurosurveillance*. 2020;25(13):2000323. doi:10.2807/1560-7917.ES.2020.25.13.2000323

3. Cylus J, Panteli D, van Ginneken E. Who should be vaccinated first? Comparing vaccine prioritization strategies in Israel and European countries using the Covid-19 Health System Response Monitor. *Isr J Health Policy Res*. 2021;10(1):16. doi:10.1186/s13584-021-00453-1

4. Emanuel EJ, Persad G, Kern A, et al. An ethical framework for global vaccine allocation. *Science*. 2020;369(6509):1309-1312. doi:10.1126/science.abe2803

5. WHO SAGE Roadmap For Prioritizing Uses Of COVID-19 Vaccines In The Context Of Limited Supply. Accessed July 15, 2021. https://www.who.int/publications-detail-redirect/who-sage-roadmap-for-prioritizing-uses-of-covid-19-vaccines-in-the-context-of-limited-supply

6. Bubar KM, Reinholt K, Kissler SM, et al. Model-informed COVID-19 vaccine prioritization strategies by age and serostatus. *Science*. 2021;371(6532):916-921. doi:10.1126/science.abe6959

7. Ehni HJ, Wiesing U, Ranisch R. Saving the most lives—A comparison of European triage guidelines in the context of the COVID-19 pandemic. *Bioethics*. 2021;35(2):125-134. doi:10.1111/bioe.12836

8. Savulescu J, Vergano M, Craxì L, Wilkinson D. An ethical algorithm for rationing life-sustaining treatment during the COVID-19 pandemic. *BJA Br J Anaesth*. 2020;125(3):253-258. doi:10.1016/j.bja.2020.05.028

9. Emanuel EJ, Persad G, Upshur R, et al. Fair Allocation of Scarce Medical Resources in the Time of Covid-19. *N Engl J Med*. 2020;382(21):2049-2055. doi:10.1056/NEJMsb2005114

10. Giubilini A, Savulescu J, Wilkinson D. Queue questions: Ethics of COVID-19 vaccine prioritization. *Bioethics*. 2021;35(4):348-355. doi:10.1111/bioe.12858

11. Bibbins-Domingo K, Petersen M, Havlir D. Taking Vaccine to Where the Virus Is—Equity and Effectiveness in Coronavirus Vaccinations. *JAMA Health Forum*. 2021;2(2):e210213-e210213. doi:10.1001/jamahealthforum.2021.0213

12. Schmidt H, Roberts DE, Eneanya ND. Rationing, racism and justice: advancing the debate around 'colourblind' COVID-19 ventilator allocation. *J Med Ethics*. Published online January 6, 2021:medethics-2020-106856. doi:10.1136/medethics-2020-106856

For peer review only - http://bmjopen.bmj.com/site/about/guidelines.xhtml

13. Mathur R, Bear L, Khunti K, Eggo RM. Urgent actions and policies needed to address COVID-19 among UK ethnic minorities. *The Lancet*. 2020;396(10266):1866-1868. doi:10.1016/S0140-6736(20)32465-X

14. White DB, Lo B. Mitigating Inequities and Saving Lives with ICU Triage during the COVID-19 Pandemic. *Am J Respir Crit Care Med*. 2021;203(3):287-295. doi:10.1164/rccm.202010-3809CP

15. Tai DBG, Shah A, Doubeni CA, Sia IG, Wieland ML. The Disproportionate Impact of COVID-19 on Racial and Ethnic Minorities in the United States. *Clin Infect Dis*. 2021;72(4):703-706. doi:10.1093/cid/ciaa815

16. Morales DR, Ali SN. COVID-19 and disparities affecting ethnic minorities. *The Lancet*. 2021;397(10286):1684-1685. doi:10.1016/S0140-6736(21)00949-1

17. Who Gets a Vaccine First? U.S. Considers Race in Coronavirus Plans - The New York Times. Accessed June 24, 2021. https://www.nytimes.com/2020/07/09/us/coronavirus-vaccine.html

18. Jagannathan M. Should Black and Latino people get priority access to a COVID-19 vaccine? MarketWatch. Accessed June 24, 2021. https://www.marketwatch.com/story/should-black-and-latino-people-get-priority-access-to-a-covid-19-vaccine-2020-07-16

19. Sederstrom N. The "Give Back": Is There Room For It? Bioethics.net. Published February 23, 2021. http://www.bioethics.net/2020/07/the-give-back-is-there-room-for-it/

20. Kaposy C, Bandrauk N. Prioritizing Vaccine Access for Vulnerable but Stigmatized Groups. *Public Health Ethics*. 2012;5(3):283-295. doi:10.1093/phe/phs010

21. Schmidt H, Gostin LO, Williams MA. Is It Lawful and Ethical to Prioritize Racial Minorities for COVID-19 Vaccines? *JAMA*. Published online October 14, 2020. doi:10.1001/jama.2020.20571

22. Opinion | Four Ways to Fix the Vaccine Rollout. *The New York Times*. https://www.nytimes.com/2021/01/07/opinion/coronavirus-vaccine-distribution.html. Published January 7, 2021. Accessed July 15, 2021.

23. Essay: Distributive justice during a pandemic – WHYY. Accessed June 24, 2021. https://whyy.org/articles/a-disturbing-medical-consensus-is-growing-heres-what-it-could-mean-for-black-patients-with-coronavirus/

24. Persad G, Emanuel EJ, Sangenito S, Glickman A, Phillips S, Largent EA. Public Perspectives on COVID-19 Vaccine Prioritization. *JAMA Netw Open*. 2021;4(4):e217943. doi:10.1001/jamanetworkopen.2021.7943

25. Williamson EJ, Walker AJ, Bhaskaran K, et al. Factors associated with COVID-19-related death using OpenSAFELY. *Nature*. 2020;584(7821):430-436. doi:10.1038/s41586-020-2521-4

For peer review only - http://bmjopen.bmj.com/site/about/guidelines.xhtml

26. U.S. Census Bureau QuickFacts: United States. Accessed July 22, 2021. https://www.census.gov/quickfacts/fact/table/US/PST045219

27. Wilkinson D, Zohny H, Kappes A, Sinnott-Armstrong W, Savulescu J. Which factors should be included in triage? An online survey of the attitudes of the UK general public to pandemic triage dilemmas. *BMJ Open*. 2020;10(12):e045593. doi:10.1136/bmjopen-2020-045593

28. Butcher B, Massey J. Why are more people from BAME backgrounds dying from coronavirus? *BBC News*. https://www.bbc.com/news/uk-52219070. Published June 19, 2020. Accessed January 31, 2022.

29. McConahay JB. Modern racism, ambivalence, and the Modern Racism Scale. In: J. F. Dovidio, S. L. Gaertner, eds. *Prejudice, Discrimination, and Racism*. Academic Press; 1986:91-125.

30. Savulescu J, Persson I, Wilkinson D. Utilitarianism and the pandemic. *Bioethics*. 2020;34(6):620-632. doi:10.1111/bioe.12771

31. Buckwalter W, Peterson A. Public attitudes toward allocating scarce resources in the COVID-19 pandemic. *PLOS ONE*. 2020;15(11):e0240651. doi:10.1371/journal.pone.0240651

32. Grover S, McClelland A, Furnham A. Preferences for scarce medical resource allocation: Differences between experts and the general public and implications for the COVID-19 pandemic. *Br J Health Psychol*. Published online June 20, 2020:e12439. doi:10.1111/bjhp.12439

33. Duch R, Roope LSJ, Violato M, et al. Citizens from 13 countries share similar preferences for COVID-19 vaccine allocation priorities. *Proc Natl Acad Sci*. 2021;118(38). doi:10.1073/pnas.2026382118

34. Crisp by R. Equality, Priority, and Compassion. *Ethics*. 2003;113(4):745-763. doi:10.1086/373954

35. Kamm FM. Equal Treatment and Equal Chances. *Philos Public Aff*. 1985;14(2):177-194.

36. Ashana DC, Anesi GL, Liu VX, et al. Equitably Allocating Resources during Crises: Racial Differences in Mortality Prediction Models. *Am J Respir Crit Care Med*. 2021;204(2):178-186. doi:10.1164/rccm.202012-4383OC

37. Sjoding MW, Dickson RP, Iwashyna TJ, Gay SE, Valley TS. Racial Bias in Pulse Oximetry Measurement. *N Engl J Med*. 2020;383(25):2477-2478. doi:10.1056/NEJMc2029240

38. Blatz CW, Ross M. Principled ideology or racism: Why do modern racists oppose race-based social justice programs? *J Exp Soc Psychol*. 2009;45(1):258-261. doi:10.1016/j.jesp.2008.08.008

39. Inc G. The Harvard Affirmative Action Case and Public Opinion. Gallup.com. Published October 22, 2018. Accessed January 31, 2022.

For peer review only

For peer review only - http://bmjopen.bmj.com/site/about/guidelines.xhtml

https://news.gallup.com/opinion/polling-matters/243965/harvard-affirmative-action-case-public-opinion.aspx

40. Savulescu J, Gyngell C, Kahane G. Collective Reflective Equilibrium in Practice (CREP) and controversial novel technologies. *Bioethics*. Published online 2021. doi:10.1111/bioe.12869

Figure legends

Figure 1. Survey Design. Participants from the US (N = 1296) or the UK (N = 1284) answered questions about their preferences for allocating scarce medical resources (online sample, aiming to be nationally representative for sex, age, race, household income, and employment – see Supplemental Methods). Fourteen percent of the UK sample, and 19% of the US sample described their racial background as non-White. A: Half the participants (N = 1262) saw scenarios relating to ventilators. They had to decide which of two potential recipients should get the ventilator. Potential recipients had different chances of surviving COVID-19 with ventilation (40%, 50%, or 70%) and one of them had an additional risk factor. Participants could give it to either one of the potential recipients or choose to toss a coin to decide. B: The other half of participants (N = 1318) saw scenarios relating to vaccines. Here, they had to decide which of two potential recipients should get the vaccine. Potential recipients had different chances of developing severe COVID-19 (10%, 15%, or 20%) and one of them had an additional risk factor. Again, participants could give it to either one of the two potential recipients or toss a coin. C: Potential recipients were either individual recipients or groups of recipients. D) Additional risk factors used in our study were racial minority status (Black in the US, BAME in the UK), being male, or being obese. The opposite additional risk factors were white, female, and healthy weight, respectively.

Figure 2. The role of chances of developing severe COVID-19 for vaccine allocation and chances of survival for ventilator allocations in the absence of additional risk factors. Participants saw either scenarios that involved individual patients (A,C) or groups of patients (B,D). For both individual patients and groups of patients, the greater the difference in chances of survival, the more participants were willing to allocate the ventilator to the potential recipient more likely to survive (A,B). Similarly, the larger the difference in chances of developing severe COVID-19 if infected, the more participants were willing to allocate the vaccine to the potential recipient at higher risk (C,D). (Error bars = 95% Confidence Interval) Note that, in these scenarios (involving only medical risk factors), we gave participants a wider range of outcome variation between recipients than in later scenarios involving additional risk factors. (For symbols, see Fig 1)

Figure 3. (A-C) Preferences for and attitudes towards prioritization of ventilators. A: Percentages of respondents willing to give the ventilator to the racial minority/male/obese patient when both patients had either a similar, or a 10% or 20% higher or lower survival chance. For comparison, we added the responses for scenarios without additional risk factors (white bars, labelled "survival chances"). B: For illustrative purposes, we subtracted the percentage of answers on the scenarios without additional risk factors from those with them. Race and sex did not elicit robust preferences, while obesity elicited negative prioritization preferences. C: Our composite attitude score mirrored the outlined findings. (**D-F) Preferences for and attitudes towards prioritization of vaccines.** D: Percentages of respondents willing to give the vaccine to the recipient with an additional risk factor when their chances of developing severe COVID-19 were either the same or were 5% or 10% higher or lower than the recipient without an additional risk factor. For comparison, we added the answers for scenarios without additional risk factors (White bars: labelled "Covid risk"). E: For illustrative purposes, we subtracted the percentage of answers on the scenarios without additional risk factors from those that contain them. A clear positive preference for each factor emerged, with obesity eliciting the strongest positive responses. F: Our composite attitude score mirrored the outlined finding. For vaccines, each factor elicited a positive preference, obesity being strongest. (Error bars = 95% Confidence Interval)

For peer review only - http://bmjopen.bmj.com/site/about/guidelines.xhtml

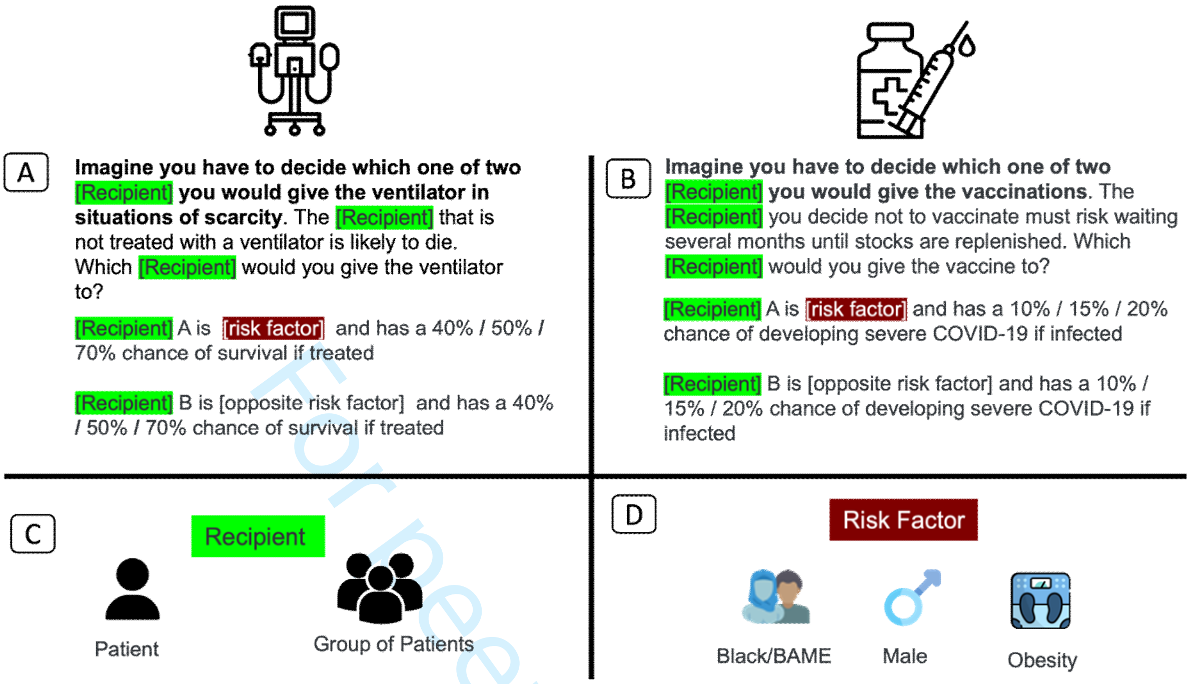

For peer review only

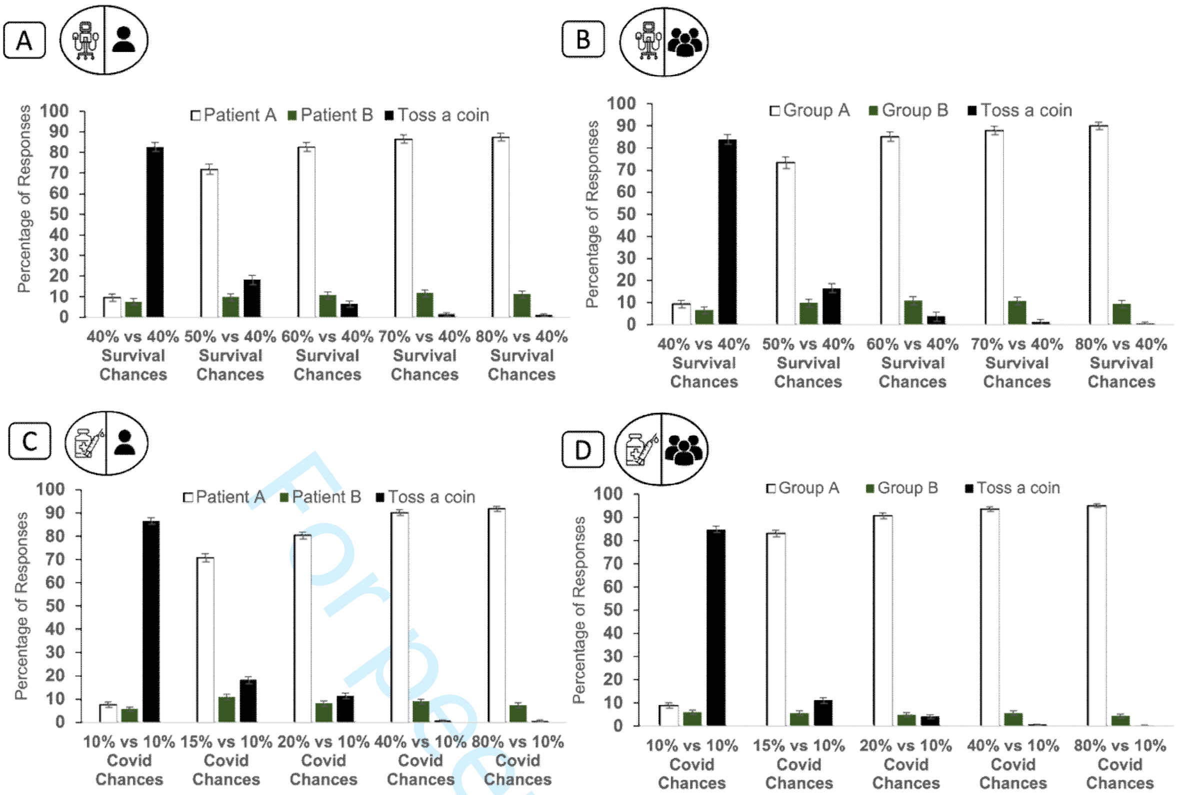

For peer review only

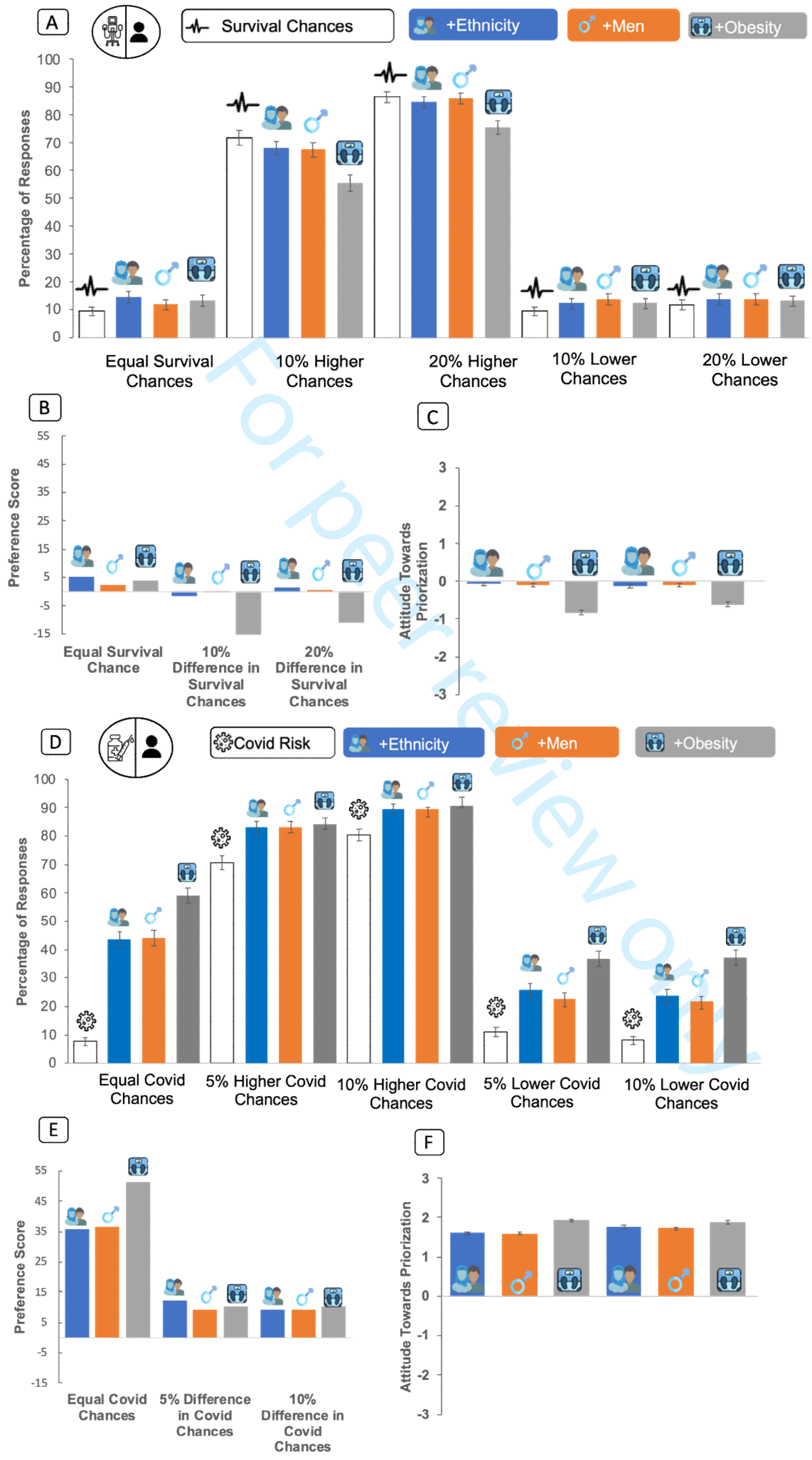
A
Survival Chances
+Ethnicity
+Men
+Obesity
Percentage of Responses
Equal Survival Chances
10% Higher Chances
20% Higher Chances
10% Lower Chances
20% Lower Chances
B
Preference Score
Equal Survival Chance
10% Difference in Survival Chances
20% Difference in Survival Chances
C
Attitude Towards Priorization
D
Covid Risk
+Ethnicity
+Men
+Obesity
Percentage of Responses
Equal Covid Chances
5% Higher Covid Chances
10% Higher Covid Chances
5% Lower Covid Chances
10% Lower Covid Chances
E
Preference Score
Equal Covid Chances
5% Difference in Covid Chances
10% Difference in Covid Chances
F
Attitude Towards Priorization

For peer review only

Supplemental Materials: Public attitudes towards race and resource allocation in a pandemic

Andreas Kappes[1], Hazem Zohny[2], Julian Savulescu[2,3], Ilina Singh[4 5], Walter Sinnott-Armstrong[6], Dominic Wilkinson[2,3,7]

Affiliations:
[1]City, University of London
[2] Oxford Uehiro Centre for Practical Ethics, University of Oxford
[3] Murdoch Children's Research Institute, Melbourne, Australia.
[4] Wellcome Trust Centre for Ethics and the Humanities, University of Oxford
[5] Department of Psychiatry, University of Oxford
[6] Kenan Institute for Ethics and Philosophy Department, Duke University
[7] John Radcliffe Hospital, Oxford, UK

Corresponding author Email: hazem.zohny@philosophy.ox.ac.uk

For peer review only

Supplemental Methods

Procedure

The survey was designed to assess participants' preferences for using medical factors as well as race, sex, obesity for allocating vaccines to people in need. Two surveys were conducted, one in the US and one in the UK. The surveys were adapted from a previous survey (4) on triage guidance involving ventilator allocations. Full survey materials can be found here: osf.io/w7bpq.

Vaccine Allocations

All participants were first introduced to the context of the allocation decisions. We wanted to make sure that all participants knew what would happen to the recipient that would not get vaccine, that all recipients wanted to get the vaccine, and that the vaccine was effective and safe. Participants read:

For the following scenarios, imagine you are working in a clinic. You are in charge of deciding which patients should get vaccinated first against COVID-19. You have one remaining dose of the vaccine left, and you must choose between two patients. The unvaccinated patient must wait several months before stocks are replenished. For the purpose of this survey, please think of the vaccine as if it had a 100% success rate in preventing COVID-19 and no side effects whatsoever. And each person under consideration wants to get vaccinated.

Participant then learned that the estimate for each recipient's chance of getting severe COVID-19 was high and was based on their age and medical history. Furthermore, they learned that their task was to decide whom to give the vaccine.

Risk of developing severe COVID-19. To determine how much weight participants would give the risk of developing severe COVID-19 for vaccine allocations, participants answered five scenarios in which the potential recipients only differed in their chance of developing severe COVID-19. In one scenario, one patient had an 80% chance and the other one a 10% chance of developing severe COVID-19 (other scenarios: 40% versus 10%, 20% versus 10%, 15% versus 10% and 10% versus 10%). Thereafter, participants were randomly introduced to one of the three additional factors: race, sex, and obesity.

Racial minority scenarios. For a racial minority in the US, we chose Black recipients and, in the UK, BAME recipients. BAME is an umbrella term, common in the United Kingdom, used to describe non-white racial groups. While a contested term, it was most consistently used in media reports on the pandemic in the UK (e.g., 5). Before participants had to decide if they wanted to give the vaccine to the member of the racial minority, we ensured that all participants were aware of the inequality in COVID-related deaths in the UK and in the US. UK participants read: "*According to the Office for National Statistics, individuals belonging to Black, Asian, and minority ethnic (BAME) groups have in general been shown to die from COVID-19 at a higher rate than that of White individuals."* US participant read: "*According to the Centre for Disease Control and Prevention, Black Americans have been shown to die from COVID-19 at a higher*

rate than of White Americans." Thereafter, participants worked on five scenarios. All of these scenarios involved one Black (BAME) and one White patient. In one scenario, both patients had the same risk of illness (10% versus 10%), in two scenarios the Black (BAME) patient had a higher risk of severe COVID-19 (5% and 10% higher), and in two scenarios the White patient a higher risk (5% and 10% higher). Participants indicated for each whether they wanted to give the vaccine to the racial minority patient, the white patient, or toss a coin.

Sex and obesity scenarios. Participants saw a similar set of scenarios for sex-based and obesity-based allocation decisions. Before working on the scenarios that involved men and women, participants read: "*According to scientific studies, men have been shown to die from COVID-19 at a higher rate than that of women."* The rationale was to inform participants that being male may be predictive of a worse outcome or survival chance if infected with COVID-19 (6). And before working on the scenarios involving obesity, participants read: "*According to scientific studies, people who are obese (severely overweight) have been shown in general to die from COVID-19 at a higher rate than that of people who have a healthy weight."* The rationale for presenting participants with this was to inform them about obesity being predictive of a worse outcome or survival chance if infected with COVID-19 (7). The five obesity-related and five sex-related scenarios followed the exact same structure as the race-related scenarios.

Group scenarios. To test if it would make a difference if participants considered only one patient, or a group of patients, we repeated the same set of scenarios for race, sex, and obesity for groups of patients. The reason for including group scenarios was twofold. First, there is a large body of evidence that suggest that people have different moral intuitions when they think about one persons versus a group of people in need (8). Second, one might argue that people might feel that the single recipient scenario for vaccine distributions is unrealistic since such decisions most often are made for large groups, rather than individuals. To ensure that such perceptions did not impact allocation preferences, we additionally included group scenarios. The scenarios followed the exact same structure as the individual ones but instead of deciding between two patients, participants had to decide between two groups of patients. As an introduction to this section of the survey, participants were told:

> *Imagine you are a policymaker for the government. At the moment, the number of vaccines available is limited and you do not have enough to protect everyone. Sometimes, policy makers face difficult decisions about which subsets of the population should be given a vaccine and which shouldn't. You will make decisions about whether or not to provide vaccines to one of two groups of people. If the people in this survey are not vaccinated, they are likely to get COVID-19 and suffer serious health consequences as a result. The group you decide not to vaccinate must risk waiting several months until stocks are replenished.*

Race, sex, and obesity scenarios. The structure of each of these scenario groups followed the structure for the respective vaccine scenario. Participants were informed about the effect race, sex, and obesity had on COVID-related deaths before working on the corresponding set of scenarios (see above). In the five race-related scenarios, participants both patients had the same chances of survival (40% versus 40%), in two scenarios the Black (BAME) patient had a higher chance of survival (10% and 20% higher), and in two scenarios the White patient a higher risk

For peer review only - http://bmjopen.bmj.com/site/about/guidelines.xhtml

chance of survival (10% and 20% higher). Participants indicated for each whether they wanted to give the vaccine to the racial minority patient, the white patient, or toss a coin. The scenarios related to sex and obesity followed the exact structure as the race-related scenarios.

Prioritization Attitude Measure. For each of the sets of scenarios, we created an attitude score by counting the number of times participants decided to give the vaccine to the person with an additional risk factor on each of the five respective scenarios. For instance, a participant that would always give the vaccine to the recipient from the racial minority would have an attitude score of 5, a participant who would allocate solely by the risk of contracting severe COVID-19 would get a 0, and a person that would always give priority to the white recipient would get a score of -5. We did this for all the patient and all the group scenarios.

Perceptions of Injustice, Responsibility, Stereotypes, and Racism

After participants worked on the scenarios, we asked them a series of questions to capture their perceptions of racial minorities, men, and obese people.

Perceptions of injustice, responsibility. To measure as how unjust people perceived the higher COVID-related death rates, we asked participants to indicate their agreement with the following statement: “Injustice was the main reason [category] individuals died more often than individuals from other groups”. To measure perceptions of responsibility of the group for the higher death rates, we asked participants to indicate their agreement with the following statement: “The main reason [category] die more often from COVID-19 is due to factors outside their control.” For both questions, scales ranged from 1 (strongly disagree) to 5 (strongly agree).

Stereotypes. According to the stereotype content model, perceptions of groups can be described along two fundamental dimensions: warmth and competence (9, 10). In line with previous research, we measured warmth by how warmly participants felt towards black (US) or BAME (UK) people, men, and obese people. We measured competence by asking participants how competent they perceived black (US) or BAME (UK) people, men, and obese people to be. We used 100-point scales for participants to indicate their perceptions.

Covert Racism - Modern Racism Scale. The modern racism scale is intended to capture subtle or covert racial attitudes or the endorsement of the idea that racial minorities are getting undeserved attention (1). Although modern racist potentially do not perceive themselves as racist, attitudes guide their decisions in situations that provide nonracially motivated reasons. Hence, the modern racism scale provides seven statements that one could endorse or reject (e.g., “Discrimination against Blacks is no longer a problem in the United States” and “Over the past few years, Blacks have gotten more economically than they deserve”). Participants are asked to express their agreement of each statement via a 5-point scale, ranging from 1 (Strongly disagree) to 5 (Strongly agree). Extensive previous research has found that scores on the modern racism scale correlate with anti-Black affect, right-wing authoritarianism, social dominance orientation, and indices of old-fashioned racism (11).

Statistical Analyses (Fixed Linear Models)

For peer review only - http://bmjopen.bmj.com/site/about/guidelines.xhtml

We ran a series of 4 linear models to explore the role of various factors in predicting racial prioritization attitudes. For each model, we defined attitudes as dependent variable and subjects as the random factor. In the first two models, we entered age, gender, race, relative income, educational level, political ideology (general, social, and economical), and type of scenario (patient versus group-based) as fixed factors. In the second two models, we additionally added perceptions of injustice, responsibility, warmth, competence, and modern racism scores (see results above).

Supplemental Results

1. Group Preferences for Vaccine and Ventilator Preferences

Patterns of response were similar for scenarios involving groups as for individual patients (Supplementary Figure 2 for individual preferences, Supplementary Figure 1 for group preferences).

For peer review only

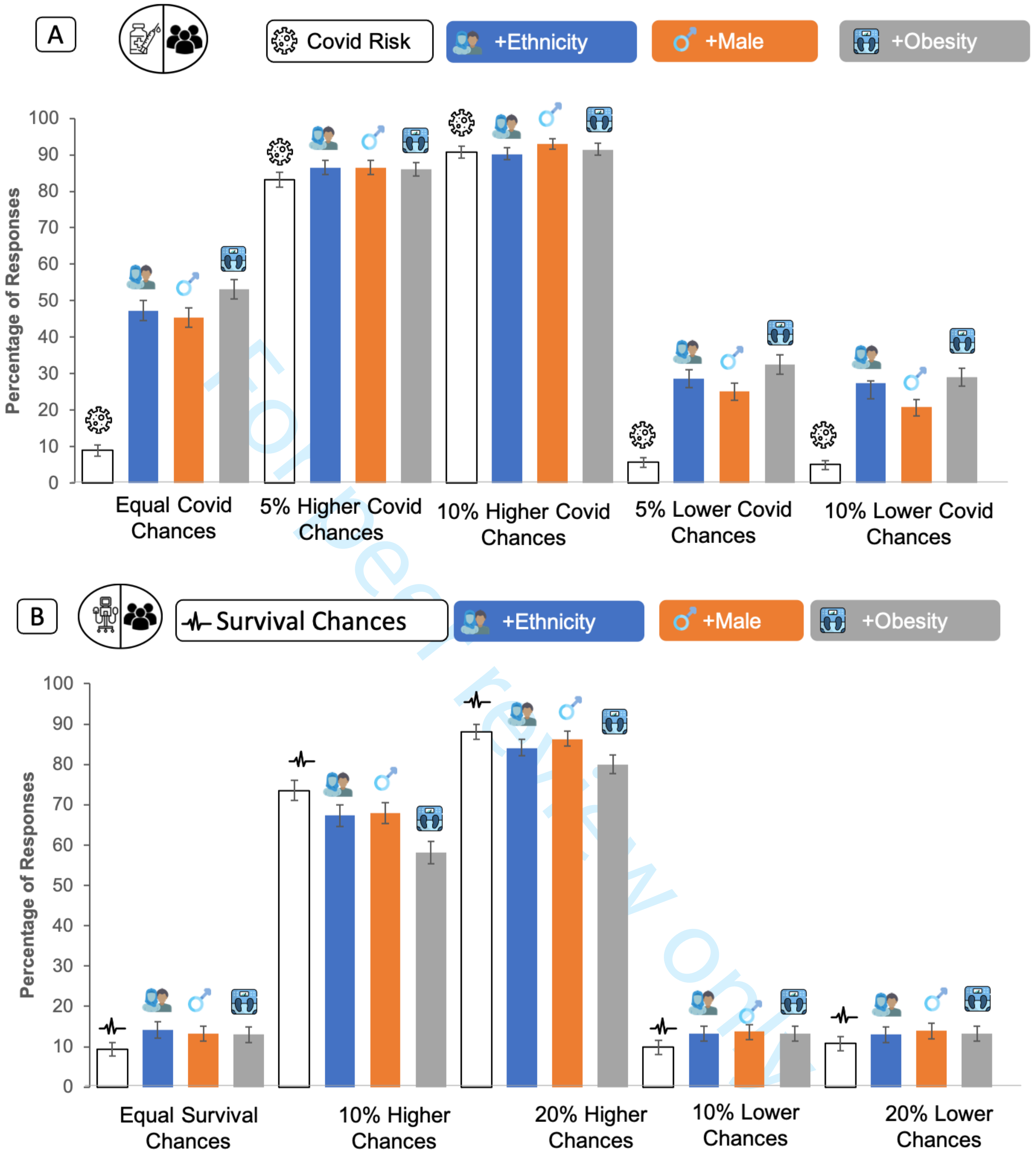

Supplementary Figure 1. A: Preferences for prioritization of vaccines. Response to scenarios where competing individual patients differed in medical risk alone or in medical risk plus an additional risk factor (race, sex, or obesity), where the patient with the additional risk factor had a 5% or 10% higher or lower chance of getting severe Covid based on medical risk alone. The bars indicate the proportion of respondents who would allocate the vaccine to a patient with a particular characteristic and risk profile. **B: Preferences for prioritization of ventilators.** A: Response to scenarios where competing individual patients differed in medical risk alone (Equal COVID chance), or in medical risk plus an additional risk factor (race, sex, or obesity), where the patient with the additional risk factor had a 10% or 20% higher or lower chance of dying based on medical risk alone. The bars indicate the proportion of respondents who would allocate the ventilator to a patient with a particular characteristic and risk profile.

For peer review only - http://bmjopen.bmj.com/site/about/guidelines.xhtml

2. General Linear Mixed Models

To test which variables impacted participants' attitudes towards risk factors, we ran a series of four linear models to explore the role of various factors in predicting racial prioritization attitudes. For each model, we defined attitudes as dependent variable and subjects as the random factor. In the first two models, we entered age, gender, race, relative income, educational level, political ideology (general, social, and economical), and type of scenario (patient versus group-based) as fixed factors. In the second two models, we additionally added perceptions of injustice, responsibility, warmth, competence, and modern racism scores. The full results of each model can be seen in Supplementary Table 1.

Supplementary Table 1. Linear Mixed Models

**Information Criteria**	**US: Vaccination Simple Model**	**US: Vaccination Full Model**	**UK: Vaccination Simple Model**	**UK: Vaccination Full Model**
-2 Log Likelihood	5253.189	4906	4846.48	4382.353
Akaike's Information Criterion (AIC)	5305.189	4968	4892.48	4438.353
Hurvich and Tsai's Criterion (AICC)	5306.273	4970	4893.34	4439.724
Bozdogan's Criterion (CAIC)	5466.048	5158	5034.64	4609.2
Schwarz's Bayesian Criterion (BIC)	5440.048	5127	5011.64	4581.2

**Information Criteria**	**US: Ventilator Simple Model**	**US: Ventilator Full Model**	**UK: Ventilator Simple Model**	**UK: Ventilator Full Model**
-2 Log Likelihood	4793.874	4733.97	3845.598	3758.101
Akaike's Information Criterion (AIC)	4847.874	4797.97	3893.598	3816.101
Hurvich and Tsai's Criterion (AICC)	4849.092	4799.68	3894.575	3817.529
Bozdogan's Criterion (CAIC)	5013.837	4994.616	4040.817	3993.85
Schwarz's Bayesian Criterion (BIC)	4986.837	4962.616	4016.817	3964.85

**American Sample: Vaccination Allocation**

For peer review only - http://bmjopen.bmj.com/site/about/guidelines.xhtml
For peer review only

	Numerator df	Denominator df	F	Sig.
Intercept	1	661	0.129	0.72
Scenario Type (Group or Patient)	1	661	63.748	< .0001
Age	1	661	0.408	0.523
Race (US)	5	661	1.706	0.131
Gender	3	661	7.238	< .0001
Highest Schooling	8	661	1.157	0.323
Social Status	1	661	0.089	0.765
Household Income	1	661	0.726	0.394
Political Orientation (General)	1	661	3.32	0.069
Political Orientation (Economical)	1	661	0.526	0.469
Political Orientation (Social)	1	661	11.382	0.001

UK Sample: Vaccination Allocation

	Numerator df	Denominator df	F	Sig.
Intercept	1	657	1.544	0.214
Scenario Type (Group or Patient)	1	657	91.289	< .0001
Age	1	657	0.233	0.63
Race (UK)	5	657	1.212	0.302
Gender	1	657	0.272	0.602
Highest Schooling	7	657	0.762	0.62
Social Status	1	657	1.985	0.159
Household Income	1	657	0.013	0.909
Political Orientation (General)	1	657	0	0.988
Political Orientation (Economical)	1	657	0.138	0.71
Political Orientation (Social)	1	657	0.244	0.621

American Sample: Vaccination – Full Model

For peer review only - http://bmjopen.bmj.com/site/about/guidelines.xhtml

	Numerator df	Denominator df	F	Sig.
Intercept	1	621	0.371	0.543
Scenario Type (Group or Patient)	1	621	61.082	< .0001
Age	1	621	1.171	0.28
Race (US)	5	621	1.328	0.25
Gender	3	621	4.702	0.003
Highest Schooling	8	621	0.678	0.711
Social Status	1	621	0.16	0.689
Household Income	1	621	0.096	0.756
Political Orientation (General)	1	621	5.125	0.024
Political Orientation (Economical)	1	621	0.008	0.927
Political Orientation (Social)	1	621	6.729	0.01
Injustice Perception	1	621	0.641	0.424
Responsibility Perception	1	621	2.589	0.108
Warmth Perception	1	621	0.012	0.912
Competence Perception	1	621	1.488	0.223
Modern Racism	1	621	23.16	< .0001

UK Sample: Vaccination – Full Model

	Numerator df	Denominator df	F	Sig.
Intercept	1	607	0.809	0.369
Scenario Type (Group or Patient)	1	607	89.533	< .0001
Age	1	607	3.054	0.081
Race (UK)	1	607	0.023	0.879
Gender	7	607	1.176	0.315
Highest Schooling	1	607	2.438	0.119
Social Status	1	607	0.091	0.763
Household Income	1	607	0.304	0.582
Political Orientation (General)	1	607	0.171	0.68
Political Orientation (Economical)	1	607	1	0.318

For peer review only - http://bmjopen.bmj.com/site/about/guidelines.xhtml

Political Orientation (Social)	5	607	0.96	0.442
Injustice Perception	1	607	0.027	0.87
Responsibility Perception	1	607	14.238	< .0001
Warmth Perception	1	607	0.048	0.826
Competence Perception	1	607	7.638	0.006
Modern Racism	1	607	16.986	< .0001

American Sample: Ventilator

	**Numerator df**	**Denominator df**	**F**	**Sig.**
Intercept	1	635	4.907	0.027
Scenario Type (Group or Patient)	1	635	2.418	0.12
Age	1	635	1.195	0.275
Race (US)	6	635	1.386	0.218
Gender	3	635	3.6	0.013
Highest Schooling	8	635	1.795	0.075
Social Status	1	635	4.437	0.036
Household Income	1	635	12.582	0
Political Orientation (General)	1	635	0.043	0.836
Political Orientation (Economical)	1	635	0.011	0.917
Political Orientation (Social)	1	635	0.976	0.324

UK Sample: Ventilator

	Numerator df	Denominator df	F	Sig.
Intercept	1	627	12.266	0
Scenario Type (Group or Patient)	1	627	1.713	0.191
Age	1	627	5.755	0.017
Race (UK)	4	627	7.181	0
Gender	3	627	0.342	0.795
Highest Schooling	7	627	0.521	0.819

For peer review only - http://bmjopen.bmj.com/site/about/guidelines.xhtml

Social Status	1	627	0.514	0.474
Household Income	1	627	0.816	0.367
Political Orientation (General)	1	627	0.793	0.373
Political Orientation (Economical)	1	627	0.002	0.962
Political Orientation (Social)	1	627	0.26	0.611

US Sample: Ventilator

	Numerator df	Denominator df	F	Sig.
Intercept	1	634	2.806	0.094
Scenario Type (Group or Patient)	1	634	2.418	0.12
Age	1	634	0.757	0.385
Race (UK)	6	634	0.897	0.497
Gender	3	634	3.411	0.017
Highest Schooling	8	634	1.624	0.115
Social Status	1	634	3.699	0.055
Household Income	1	634	11.868	0.001
Political Orientation (General)	1	634	0.039	0.843
Political Orientation (Economical)	1	634	0.785	0.376
Political Orientation (Social)	1	634	0.047	0.829
Injustice Perception	1	634	0.778	0.378
Responsibility Perception	1	634	0.382	0.537
Warmth Perception	1	634	1.014	0.314
Competence Perception	1	634	9.688	0.002
Modern Racism	1	634	10.737	0.001

UK Sample: Ventilator

	Numerator df	Denominator df	F	Sig.
Intercept	1	624	1.419	0.234
Scenario Type (Group or Patient)	1	624	1.926	0.166

For peer review only - http://bmjopen.bmj.com/site/about/guidelines.xhtml

Age	1	624	1.316	0.252
Race (UK)	3	624	0.864	0.46
Gender	7	624	1.241	0.278
Highest Schooling	1	624	0.066	0.797
Social Status	1	624	0.913	0.34
Household Income	1	624	0.096	0.757
Political Orientation (General)	1	624	0.038	0.846
Political Orientation (Economical)	1	624	0.59	0.443
Political Orientation (Social)	4	624	4.889	0.001
Injustice Perception	1	624	2.732	0.099
Responsibility Perception	1	624	0.744	0.389
Warmth Perception	1	624	3.129	0.077
Competence Perception	1	624	4.65	0.031
Modern Racism	1	624	13.881	< .0001

3. Perceptions of Injustice, Responsibility, and Stereotypes

In the US and the UK, participants were more likely to believe that adverse COVID-19 related health outcomes were the result of injustice and outside the individual's control for race than for obesity/male sex [Supplementary Figure 2]. In particular, injustice was perceived differently for the three additional risk factors ($F(2, 2577)=217.388$, $P <0.0001$, partial $eta^2 =0.144$). Bonferroni-corrected posthoc comparisons indicated that participants most strongly attributed the increased numbers of deaths of racial minorities due to injustice, stronger than the deaths of men ($P < .0001$, 95% CI: 0.372-0.469), and obese people ($P < 0.000$, 95% CI: 0.300-0.399). For the latter two, participants indicate more injustice to obese people death's than to men ($P < .0001$, 95% CI: 0.035-0.108). Similarly, there were differences in perceived personal control for adverse outcomes from COVID ($F(2, 2577)=284.123$, $P <.0001$, partial $eta^2 = .181$). Racial minorities were perceived to have the least control over the higher death rate compared to men ($P =0.009$, CI 95: 0.011-0.103) and obese people ($P < .0001$, 95% CI:0.529-0.655). Obese people were perceived to have most control over the higher death rates attributable to COVID, higher control than men ($P < .0001$, 95% CI: 0.595-0.476) and people from racial minorities ($P =0.009$, 95% CI: 0.011-0.103).

For peer review only - http://bmjopen.bmj.com/site/about/guidelines.xhtml

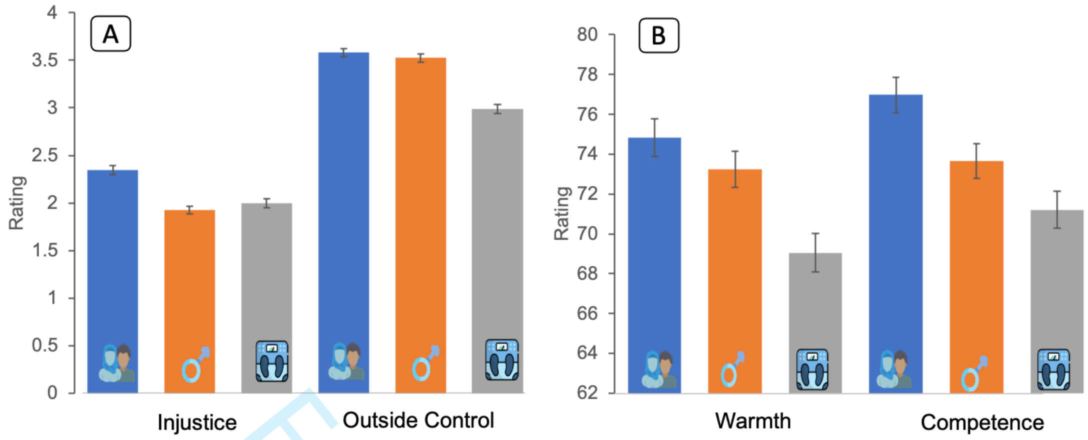

Supplementary Figure 2: Perceptions of Injustice, Control, Warmth, and Competence. A: Bonferroni-corrected comparisons indicated that participants most strongly attributed the increased numbers of deaths of racial minorities to injustice, stronger than the deaths of males (P < .0001, 95% CI: 0.372-0.469), and obese people (P < 0.000, 95% CI: 0.300-0.399). Participants attributed slightly more injustice to obese people's deaths than to males' deaths (P < .0001, 95% CI: 0.035-0.108). Similarly, racial minorities were perceived to have less control over the higher death rate compared to males (P =0.009, CI 95: 0.011-0.103) and obese people (P < .0001, 95% CI:0.529-0.655). Obese people were perceived to have the most control over higher death rates attributable to COVID-19, more than males (P < .0001, 95% CI: 0.595-0.476) and racial minorities (P =0.009, 95% CI: 0.011-0.103). B: Survey participants had the strongest stereotype content feelings of warmth towards ethical minorities, compared to attitudes towards males (p =0.005, Bonferroni corrected, small difference) and obese people (P < 0.0001, Bonferroni corrected). Participants also expressed higher feelings of competence toward individuals from a racial minority than males (P < 0.0001, Bonferroni corrected), or obese people (P < 0.0001, Bonferroni corrected). (Error bars = 95% Confidence Interval).

How did these perceptions of injustice and responsibility differ between the US and the UK? For injustice, we found no main effect of country ($F(1,2576) = 2.534$, $P = .112$, partial $eta^2 = .001$) and no interaction effect with categories of disadvantage ($F(1,2576) = 1.622$, $P = 0.203$, partial $eta^2 = .001$). Participants in both countries perceived the relative injustice that affected each group similarly. When examining responsibility, we found no main effect of country ($F(1,2576) = 0.366$, $P = 0.545$, partial $eta^2 = 0.00001$), but an interaction between country and category ($F(1,2576) = 31.651$, $P < .0001$, partial $eta^2 = 0.012$). Participants in the US gave men less responsibility ($t(2578) = 4.091$, $P < 0.0001$), but obese people more responsibility ($t(2578) = 4.176$ .000, $P < 0.0001$) than participants in the UK.

Perceptions of injustice and responsibility might be related to the general attitudes towards a particular category. Attitudes can be characterized via two different dimensions, warmth and competence[56,57]**.** We found that again, respondents had different feelings of warmth towards different categories ($F(1, 2577) = 72.446$, $P < 0.0001$, partial $eta^2 = .029$) and competence ($F(1, 2577) = 76.369$, $P < 0.0001$, partial $eta^2 = 0.030$). In particular, survey participants had strongest feelings of warmth towards ethical minorities, compared to feelings towards men (p =0.005, Bonferroni corrected, small difference) and obese people (P < 0.0001, Bonferroni corrected), and rated the warmth of obese people lower than the warmth of men (P < 0.0001, Bonferroni corrected). When examining competence, we found a similar effect of group membership ($F(2,2575) = 77.00$, $P < 0.0001$, partial $eta^2 = .03$). Participants expressed higher feelings of competence in individuals from a racial minority than men (P < 0.0001, Bonferroni corrected), or obese people (P < 0.0001, Bonferroni corrected), while men were perceived as more competent than obese people (P < 0.0001, Bonferroni corrected).

For peer review only - http://bmjopen.bmj.com/site/about/guidelines.xhtml

Again, one might wonder whether participants from the US and the UK had different stereotypes about racial minorities, men, and obese people. When examining the effect of country on warmth, we find a main effect of country, $F(1,2497) = 19.408$, $P < 0.0001$, partial $eta^2 = .008$, American participants gave higher warmth ratings overall, though this varied between categories, $F(2,2497) = 15.083$, $P < .0001$, partial $eta^2 = 0.006$. The biggest difference was in warmth perception of obese participants, with American participants rating obese people more warmly than British participants ($t(2495) = 5.73$, $P < .001$), and only very small differences for the other two categories between both countries. When examining the effect of country and competence ratings, a similar picture emerged. We found a main effect for country ($F(2,2497) = 25.16$, $P <.0001$, partial $eta^2 = .01$, which was qualified by an interaction effect with disadvantage categories, $F(2,2497) = 17.49$, $P <001$, partial $eta^2 = .007$). Again, the main difference was for perceptions of obese people, Americans perceived obese people as more competent than British people, $t(2497) = 6.672$, $P < .0001$).

Why do people perceive the effect of a specific additional risk factor such as race as unjust? Part of the reason might be because of how favorably one thinks about the additional risk factor in general. To test this idea, we regressed feelings of responsibility, warmth, and competence on perceptions of injustice for each of the three additional risk factors. For race, we found that perceptions of responsibility (beta = .130, $t(2482) = 6.551$, $P <.0001$) and feelings of warmth, (beta = .209, $t(2482) = 6.89$, $P < .0001$) predicted the perception of injustice, but not perceptions of competence (beta = -.039, $t(2482) = 1.309$, $P =0.191$). For men, neither responsibility (beta = .032, $t(2482) = 1.589$,$P = 0.112$), competence (beta-.054, t-1.930 $P = 0.054$), or warmth (beta = .035, t1.262, $P = 0.207$) predicted perceptions of injustice. In contrast, for obesity, responsibility (beta = .222, $t(2482) = 11.047$, $P < 0.0001$), warmth (beta = .103, $t(2482) = 3.587$, $P < 0.0001$), and competence (beta = -.074, $t(2482) =2.624$, $P = 0.009$) predicted perceptions of injustice.

4. Allocation attitudes for participants with low and high modern racism scores

In the US and the UK, responses on the Modern Racism Scale were associated with attitudes towards ventilator and vaccine allocation based on race (Ventilator: US: $F(1,634) = 10.73$, $p = 0.001$, UK: $F(1,624) = 13.88$, $P < .0001$; Vaccine: US: F(1,621 =23.00, $P <0.0001$, UK: $F(1,607)=16.98$, $P <0.0001$). The more participants endorsed modern racism statements, the less positive their composite attitudes towards ventilator or vaccine allocation to racial minority patients. A minority (27.3%) of participants had a mean score of higher than three on the modern racism scale, indicating an endorsement of modern racism. These participants did not give weight to race in ventilator or vaccine allocations [Supplementary Figure 3]. Participants with low modern racism scores also did not give weight to race in ventilator allocation but did for vaccines. Modern racism did not affect prioritisation based on sex/obesity [Supplementary Figure 3]. Perceptions of injustice did not predict ventilator or vaccine allocation attitudes in the US ($F(1,634) = 0.778$, $P = 0.378$) or the UK ($F(1,624) = 2.732$, $P = 0.099$).

For peer review only - http://bmjopen.bmj.com/site/about/guidelines.xhtml

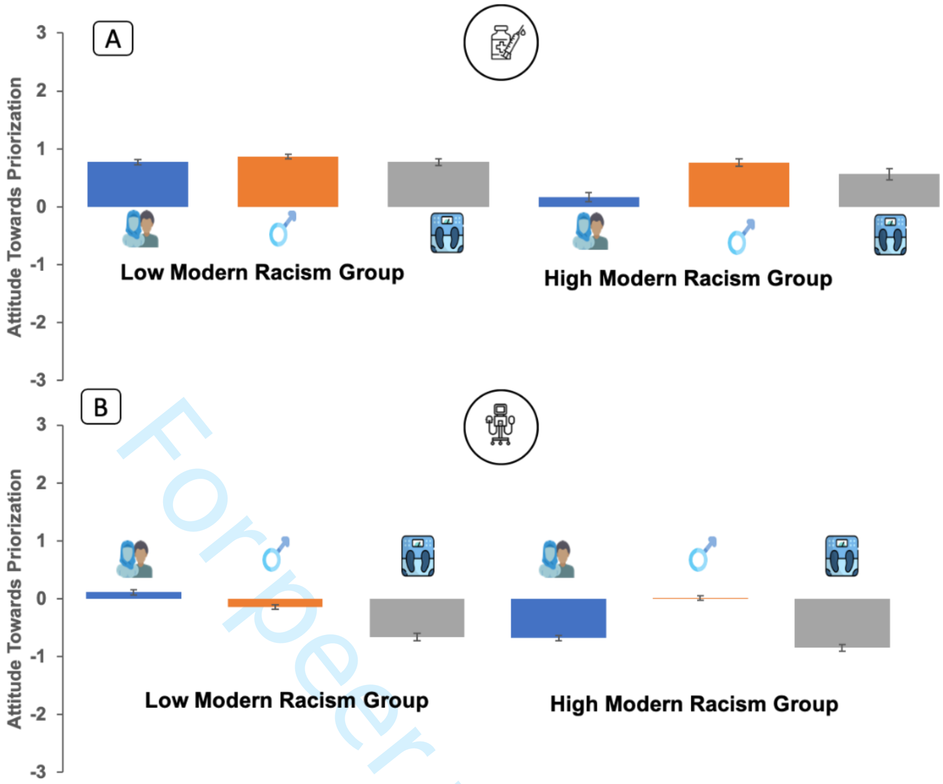

Supplementary Figure 3: Allocation attitudes for participants with low modern racism scores (Low Modern Racism Group) and high modern racism scores (High Modern Racism Group). A: For vaccines, participants in the Low Modern Racism Group (mean score lower than scale midpoint, N = 1011) had a more positive attitude towards race-based vaccine allocation compared to participants in the High Modern Racism Group (mean score higher than scale midpoint, N = 307). Allocation attitudes for sex and obesity were similar. B: For ventilators, we find that participants in the Low Modern Racism Group (N = 919) had a neutral attitude towards race-based vaccine allocation, while participants in the High Modern Racism Group (N = 343) had a negative one. Allocation attitudes for sex and obesity were similar.

References (Supplemental Materials)

1. J. B. McConahay, “Modern racism, ambivalence, and the Modern Racism Scale” in *Prejudice, Discrimination, and Racism*, J. F. Dovidio, S. L. Gaertner, Eds. (Academic Press, 1986), pp. 91–125.

2. T. G. Morrison, M. Kiss, Modern racism scale. *Encyclopedia of personality and individual differences*, 1–3 (2017).

3. , U.S. Census Bureau QuickFacts: United States (July 22, 2021).

4. D. Wilkinson, H. Zohny, A. Kappes, W. Sinnott-Armstrong, J. Savulescu, Which factors should be included in triage? An online survey of the attitudes of the UK general public to pandemic triage dilemmas. *BMJ Open* **10**, e045593 (2020).

For peer review only

5. , Why are more people from BAME backgrounds dying from coronavirus? *BBC News* (2020) (June 28, 2021).

6. T. Takahashi, *et al.*, Sex differences in immune responses that underlie COVID-19 disease outcomes. *Nature* **588**, 315–320 (2020).

7. J. Yang, J. Hu, C. Zhu, Obesity aggravates COVID-19: a systematic review and meta-analysis. *Journal of medical virology* **93**, 257–261 (2021).

8. P. Slovic, "If i look at the mass i will never act: Psychic numbingpsychic numbing and genocidegenocide" in *Emotions and Risky Technologies*, (Springer, 2010), pp. 37–59.

9. S. T. Fiske, A. J. Cuddy, P. Glick, J. Xu, "A model of (often mixed) stereotype content: Competence and warmth respectively follow from perceived status and competition (2002)" in *Social Cognition*, (Routledge, 2018), pp. 171–222.

10. S. T. Fiske, A. J. Cuddy, P. Glick, Universal dimensions of social cognition: Warmth and competence. *Trends in cognitive sciences* **11**, 77–83 (2007).

11. C. W. Blatz, M. Ross, Principled ideology or racism: Why do modern racists oppose race-based social justice programs? *Journal of Experimental Social Psychology* **45**, 258–261 (2009).

For peer review only

For peer review only - http://bmjopen.bmj.com/site/about/guidelines.xhtml

BMJ Open

Race and resource allocation: An online survey of US and UK adults' attitudes towards COVID-19 ventilator and vaccine distribution

Journal:	*BMJ Open*
Manuscript ID	bmjopen-2022-062561.R1
Article Type:	Original research
Date Submitted by the Author:	08-Jun-2022
Complete List of Authors:	Kappes, Andreas; City University of London<br>Zohny, Hazem; University of Oxford,<br>Savulescu, Julian; University of Oxford, MEDICAL ASSESSMENT UNIT<br>Singh, Ilina; University of Oxford, Psychiatry<br>Sinnott-Armstrong, Walter; Duke University<br>Wilkinson, Dominic; University of Oxford, Oxford Uehiro Centre for Practical Ethics; John Radcliffe Hospital,
**Primary Subject Heading**:	Ethics
Secondary Subject Heading:	Public health
Keywords:	Health policy < HEALTH SERVICES ADMINISTRATION & MANAGEMENT, MEDICAL ETHICS, COVID-19

SCHOLARONE™
Manuscripts

I, the Submitting Author has the right to grant and does grant on behalf of all authors of the Work (as defined in the below author licence), an exclusive licence and/or a non-exclusive licence for contributions from authors who are: i) UK Crown employees; ii) where BMJ has agreed a CC-BY licence shall apply, and/or iii) in accordance with the terms applicable for US Federal Government officers or employees acting as part of their official duties; on a worldwide, perpetual, irrevocable, royalty-free basis to BMJ Publishing Group Ltd ("BMJ") its licensees and where the relevant Journal is co-owned by BMJ to the co-owners of the Journal, to publish the Work in this journal and any other BMJ products and to exploit all rights, as set out in our licence.

The Submitting Author accepts and understands that any supply made under these terms is made by BMJ to the Submitting Author unless you are acting as an employee on behalf of your employer or a postgraduate student of an affiliated institution which is paying any applicable article publishing charge ("APC") for Open Access articles. Where the Submitting Author wishes to make the Work available on an Open Access basis (and intends to pay the relevant APC), the terms of reuse of such Open Access shall be governed by a Creative Commons licence – details of these licences and which Creative Commons licence will apply to this Work are set out in our licence referred to above.

Other than as permitted in any relevant BMJ Author's Self Archiving Policies, I confirm this Work has not been accepted for publication elsewhere, is not being considered for publication elsewhere and does not duplicate material already published. I confirm all authors consent to publication of this Work and authorise the granting of this licence.

For peer review only

Race and resource allocation: An online survey of US and UK adults' attitudes towards COVID-19 ventilator and vaccine distribution

Andreas Kappes PhD[1], Hazem Zohny PhD[2], Julian Savulescu PhD[2,3], Ilina Singh PhD[4,5], Walter Sinnott-Armstrong PhD[6], Dominic Wilkinson DPhil[2,3,7]

Affiliations:
[1]City, University of London
[2] Oxford Uehiro Centre for Practical Ethics, University of Oxford
[3] Murdoch Children's Research Institute, Melbourne, Australia.
[4] Wellcome Trust Centre for Ethics and the Humanities, University of Oxford
[5] Department of Psychiatry, University of Oxford
[6] Kenan Institute for Ethics and Philosophy Department, Duke University
[7] John Radcliffe Hospital, Oxford, UK

Correspondence: Dr Hazem Zohny, Oxford Uehiro Centre for Practical Ethics, Suite 8, Littlegate House, St Ebbes St, Oxford, OX1 1PT, UK. Tel: +44 1865 286888, Email: hazem.zohny@philosophy.ox.ac.uk

For peer review only

Abstract

Objective: This study aimed to assess US and UK adults' attitudes towards COVID-19 ventilator and vaccine allocation.

Design: Online survey including US and UK adults, sampled to be representative for sex, age, race, household income, and employment. A total of 2580 participated [female = 1289, Age Range = 18 to 85].

Interventions: Participants were asked to allocate ventilators or vaccines in scenarios involving individuals or groups with different medical risk and additional risk factors.

Results: When asked to allocate ventilators to a racial minority or white patient with identical chances of survival, 14.8% allocated to the minority patient, 16.2% chose the white patient, and 68.9% chose to toss a coin. When the racial minority patient had a 10% lower chance of survival, 12.4% participants allocated the ventilator to the minority patient. When asked to allocate vaccines to a racial minority or white patient with identical risk of severe COVID-19, 43.6% allocated to the minority patient, 7.2% chose the white patient, and 49.2% chose a coin toss. When the racial minority patient had a 10% lower risk of severe COVID-19, 23.7% participants allocated the vaccine to the minority patient. Similar results were seen for obesity or male sex as additional risk factors. Participant race did not impact vaccine or ventilator allocation decisions in the US, but did impact ventilator allocation attitudes in the UK ($F(4,602) = 6.95$, $P < 0.001$). In both countries, responses on the Modern Racism Scale were strongly associated with attitudes towards race-based ventilator and vaccine allocations ($P < 0.0001$).

Conclusions: Although living in countries with high racial inequality during a pandemic, most US and UK adults in our survey allocated ventilators and vaccines preferentially to those with the highest chance of survival or highest chance of severe illness. Few participants gave additional weight to race for ventilator allocation, but race of recipient led to vaccine prioritisation in cases where risk of illness was similar.

Strengths and limitations of this study:

- Our scenarios enable comparison of approaches to prioritisation of ventilators and vaccines in the face of scarcity and high demand
- The survey provides insights into the relative weights given to equity and race compared to medical factors in two different countries.
- To control variables, hypothetical scenarios were not realistic and may not align with lived experience.
- Survey responses permit quantification of attitudes to prioritisation, but not the reasons behind answers.
- Although we sought large nationally representative samples, Hispanic and Black participants were under-represented among US respondents.

Introduction

For peer review only - http://bmjopen.bmj.com/site/about/guidelines.xhtml

The coronavirus pandemic has raised contentious ethical questions about the distribution of scarce healthcare resources during a crisis. For example, early concern about the supply of intensive care beds and mechanical ventilators led healthcare systems to develop triage criteria in the event of a shortage.[1,2] Later, states had to decide whom to prioritise for access to the limited supply of vaccines.[3,4] Although different countries have followed different strategies, a prevailing response to these questions has focused on saving the most lives.[3,5–8] For ventilator triage, this entailed giving priority to those with the highest chance of survival.[9] For vaccines, those with the highest risk of severe illness were prioritised.[10]

However, this has been criticised[11] for ignoring evidence that individuals from racial minorities have been disproportionately affected by the pandemic.[12–14] In the US, Black, Hispanic and Indigenous people were more likely to be infected, hospitalised and die of COVID-19 than White individuals.[15] Similarly, high morbidity and mortality rates were seen in ethnic minority groups in the UK.[16] This raises an important ethical question: should individuals from disproportionately affected racial minorities be prioritised when allocating scarce resources such as ventilators and vaccines? If so, how should this be weighed against other ethical values in resource allocation, including the desire to save the most lives and the need to treat patients equally?

Some legal scholars, policy advocates, and ethicists have suggested that race should be factored into ventilator and vaccine allocation.[17–19] For example, some have proposed positive discrimination in the form of equity weights.[12] However, such an approach might raise political[20] or legal concerns.[21] Alternatively, it could lead to ethical questions. Treating members of racial groups differently would conflict with principles of equality. Furthermore, in some circumstances, preferential allocation to members of a disadvantaged group could increase overall mortality from COVID-19[22,23]. On the other hand, not considering it could widen the race-based difference in COVID-19 deaths.

Policy discussion during the pandemic has largely lacked information on public opinion: it is not known what people in countries like the US and UK, with high racial inequality in COVID-19 deaths, think about these questions. There is some evidence that the public support including race in allocation of vaccines,[24] but none about the *relative* importance of medical versus racial factors.

We sought to examine the relative weight given by the public to race in allocation and to compare this with participants' views on obesity and male sex. Race, obesity, and sex are independent risk factors for COVID-19 (in one UK study, hazard ratios for COVID-19 death were 1.48, 1.40, and 1.59, respectively[25]), but they differ ethically (for example, in their association with historical injustice). Finally, we sought to explore whether prioritisation preferences differed between racial groups, socio-economic status, or political orientations or were related to stereotypes and covert racial attitudes. This would illuminate what drives prioritisation attitudes but also whether a broader consensus exists.

For peer review only - http://bmjopen.bmj.com/site/about/guidelines.xhtml

Methods

The study was approved by the University of Oxford Central University Research Ethics Committee [R73841/RE002]. It follows the American Association for Public Opinion Research (AAPOR) reporting guideline. All data, code, and materials used in the analysis is are available in a public, open access repository.[26]

Participants were recruited from large market research panels and paid $10.8/hour. Speed and attention checks were used to identify and exclude respondents not paying sufficient attention to question details.

Two surveys were conducted in December 2020/January 2021, (US n=1296, UK n=1284). (Table 1). Each sub-sample size gave the ability to estimate true preferences with a 4% margin of error (95% confidence interval) for the US and the UK population. The survey achieved a completion rate (completed surveys divided by number of respondents who entered the survey) of 86.7%. As the number of people that had the chance to participate in the opt-in panel is not known, we cannot report the AAPOR RR1[9].

Table 1: Demographic Details of Participants. Participants were sampled to be representatives of the US and UK general population for sex, age, race, household income, and employment. (Different income categories were used to reflect known information on population characteristics). Fourteen percent of the UK sample, and 19% of the US sample described their racial background as non-White. Note that, compared to the last census[27], the US sample had a lower proportion of Hispanic and Black participants than the general US population.

	US / UK	US Sample	UK Sample
		n (%)	n (%)
Gender	Male	634 (48.9%)	630 (49.1%)
	Female	656 (50.6%)	650 (50.6%
	Other	3 (0.2%)	3 (0.2%)
	Prefer not to say	3 (0.2%)	1 (0.1%)
Race	White	1034 (79.8%)	1103 (85.9%)
	Hispanic or Latino	70 (5.4%)	-
	Black or African American / Black or Black British	114 (8.8%)	41 (3.2%)
	Native American or American Indian	19 (1.5%)	-
	Mixed	-	29 (2.3%)
	Asian or Pacific Islander / Asian or Asian British	23 (1.8%)	98 (7.6%)
	Other	21 (1.6%)	11 (0.9%)
	Prefer not to say	11 (0.8%)	2 (0.2%)
Age	18 - 24	159 (15.5%)	145 (11.3%))
	25 - 34	248 (18.9%)	303 *23.6%)
	35 - 44	237 (18.9%)	291 (*22.7%)
	45 - 54	252 (18.3%)	238 (18.5%)
	55 - 64	224 (14.2%)	198 (15.4%)
	65 - 74	129 (5.9%)	89 (6.9%)
	75 - 84	38 (1.6%)	20 *1.6%)
	85 or older	4 (0.6%)	-
Highest level of schooling	Less than high school degree / Primary school	24 (1.9%)	3 (0.2%)
	High school graduate or equivalent / Secondary school up to 16 years	237 (18.3%)	236 (18.4%)
	Some college but no degree / Higher or secondary or further education	250 (19.3%)	441 (34.3%))
	Associate degree in college (2-year)	122 (9.4%)	-

For peer review only - http://bmjopen.bmj.com/site/about/guidelines.xhtml

	Bachelor's degree	160 (12.3%)	381 29.7%)
	Master's degree	290 (22.4%)	138 (10.7%)
	Doctoral degree	29 (2.2%)	29 (2.3%)
	Professional degree (JD, MD)	38 (2.9%)	47 (3.7%)
	Prefer not to say	6 (0.5%)	9 (0.7%)
	Other	140 (10.8%)	-
**Employment status**	Employed part time	136 (10.5%)	735 (57.2%)
	Employed full time	617 (47.6%)	232 (18.1%)
	Unemployed looking for work	96 (7.4%)	70 (5.5%)
	Unemployed not looking for work	113 (8.7%)	96 (7.5%)
	Retired	274 (21.1%)	114 (8.9%)
	Student	48 (3.7%)	37 (2.9%)
	Disabled	12 (0.9%)	-
**Estimated Household Income**	\$0-\$25k / £0-£20k	230 (17%)	263 (20.5%)
	\$25k-\$50k / £20k-£30k	274 (21.1%)	276 (21.5%)
	£30k-£40k	-	198 (15.4%)
	£40k-£50k	-	145 (11.3%)
	\$50k-\$75k / £50k-£60k	224 (17.3%)	106 (8.3%)
	\$75k-\$100k / £60k-£100k	168 (13%)	191 (14.9%)
	\$100k-\$150k / £100k+	199 (15.4%)	105 (8.2%)
	\$150k+	103 (7.9%)	-
	Prefer not to say	11 (0.8%)	-

The surveys were adapted from a previous study.[28] Participants imagined that they needed to make decisions in the setting of a severe shortage of ventilators or vaccines. To examine how much weight participants would give to predicted chance (based on age and comorbidity) of survival for allocating ventilators, or of developing severe COVID-19 for vaccines, participants read scenarios where potential recipients differed in those variables to different degrees. For example, in one ventilator scenario, one patient had a 50% chance of survival, the other a 40% chance. In a corresponding vaccine scenario, participants had a 20% vs 10% chance of developing severe COVID-19. Participants decided whom to give the ventilator or vaccine to or to toss a coin (Figure 1). Thereafter, participants were randomly introduced to one of the three additional factors: race, sex, and obesity.

For a racial minority in the US, we chose 'Black recipients' and, in the UK, 'BAME recipients'. BAME is a common term in the United Kingdom, used to describe non-white racial groups.[29] Before completing scenarios, we reminded participants of the racial inequality in COVID-19-related deaths in the UK and in the US. Thereafter, participants worked on five scenarios involving one Black (BAME) and one White patient. In one scenario, both patients had the same chance of surviving/severe COVID-19; in two scenarios the Black (BAME) patient had a higher chance of survival/severe COVID-19; in two scenarios the White patient had a higher chance. Participants indicated for each whether they wanted to give the ventilator/vaccine to the patient from a racial minority, the white patient, or toss a coin.

Participants saw a similar set of scenarios for sex and obesity as additional risk factors [SM]. To test whether it would make a difference, we repeated the scenarios for groups of patients.

We asked participants questions to capture their perceptions of racial minorities, men, and obese people, including their views about the degree to which worse outcomes from COVID-19 were a result of social injustice or under the control of the individual. We also administered the modern racism scale, intended to capture subtle or covert discriminatory

For peer review only - http://bmjopen.bmj.com/site/about/guidelines.xhtml

attitudes.[30] The modern racism scale is one of the most commonly used and best validated instruments to examine prejudice against black people in the US.[31]

Statistical analysis

To examine the differences between risk factors, and the variables that impacted participants' attitudes, we created a composite attitude score for each set of scenarios by counting the number of times participants decided to give the ventilator or vaccine to the person or group with an additional risk factor in successive scenarios. To test whether differences existed between risk factors, we used repeated measure analysis of variance with Bonferroni corrected post-hoc comparisons. To test which variables impacted participants' attitudes towards risk factors, we ran a series of four linear models to explore the role of various factors in predicting racial prioritisation attitudes. For each model, we defined attitudes as dependent variable and subjects as the random factor. In the first two models, we entered age, gender, race, relative income, educational level, political ideology (general, social, and economical), and type of scenario (patient versus group-based) as fixed factors. In the second two models, we additionally added perceptions of injustice, responsibility, warmth, competence, and modern racism scores. The full results of each model can be seen in the Supplemental Results (Supplementary Table 1). We used SPSS 28 for all analyses.

Patient and public involvement

No patient involvement.

Results

Prioritisation of medical risk

We first assessed the impact of medical risk factors on allocation. Asked to choose between patients 50% vs 40% chance of surviving if treated, 71.9% (95% CI: 69.3%-74.3%,) of participants gave the ventilator to the patient with higher chances. For vaccines (15% vs 10% chance of severe COVID-19), 70.8% (95% CI: 68.3%-73.2%) gave the vaccine to the higher risk recipient. This increased when the difference between patients increased (Figure 2). When there was no difference, 82.7 (86%) (95% CI: 80.5-84.8,84.6-88.4) tossed a coin. Patterns of response were very similar for scenarios involving groups as for individual patients (Figure 2).

Risk factors and resource allocation

In scenarios involving patients with identical chances of survival but different race, 14.8 of participants allocated the ventilator to Black or BAME patients, 68.9 chose to toss a coin, and 16.2 chose the White patient. For vaccine allocation to patients with identical chances of severe illness, 43.6% of participants allocated the vaccine to Black or BAME patients, 49.2% chose to toss a coin, and 7.2% chose the White patient. When the racial minority patient had a lower chance of survival or severe illness, <15% of respondents allocated the ventilator or vaccine to them (Figure 3).

For peer review only - http://bmjopen.bmj.com/site/about/guidelines.xhtml

When chances of survival were equal, but one patient was male and the other female, 11.9% (95% CI: 10.2%,13.8%) of participants gave the ventilator to the male, (22.7% female patient; 65.4% tossed a coin). When obesity was an additional risk factor, 11.9% (95% CI: 11.6% - 15.5%) chose the obese patient, (41.1% healthy weight, 45.4% tossed a coin). For vaccines, in scenarios of equal medical risk, where one patient was male and the other female, 44.2% of participants gave the vaccine to the male patient. When obesity was an additional risk factor, 59.2% allocated to an obese patient (Figure 3).

Using the composite attitude score, race and sex did not affect allocation decisions for ventilators, but obesity did so negatively: ($P < .001$, 95% CI -.9485, -.7124). Participants had lower prioritisation preferences for obese patients, (compared with racial minority, $P < 0.001$, and male patients, P =.030). For vaccines, participants had significant prioritisation preference for race, sex, and obesity (attitude greater than zero: $p < .00001$; post-hoc analyses: obese patients > racial minorities, $P < 0.001$, and male patients, $P < 0.001$.) There was a very small main effect for scenario type (group preference – Supplementary Figure 1). Country of participants (US vs UK) did not alter allocation attitudes ($F(1,1260) = 0.619$, P =0.431).

Determinants of prioritisation

Using mixed linear models [Supplementary Table 1, full results], we find that race of participant impacted ventilator allocation attitudes in the UK ($F(4,602) = 6.95$, $P < 0.001$) but not the US ($F(6, 635) = 1.38$, $P = 0.21$). In the UK, Asian and Black participants had more positive attitudes towards race in allocation of ventilators than white participants [SM]. Among BAME participants, 22.1 % of participants gave the ventilator to a BAME patient with a 10% lower chance of survival, while only 6.9% of the White participants did (see Table 2).

Table 2: Differences in responses between UK White and ethnic minority participants

	Ethnic Minority Participants			White Participants		
	BAME Patient	Coin Toss	White Patient	BAME Patient	Coin Toss	White Patient
Equal Survival Chances	24.4	72.1	3.5	13	73.2	13.8
10% Higher Chance	77.9	11.6	10.5	73.2	15.3	11.5
30% Higher Chance	87.2	10.5	2.3	90.5	2	7.4
10% Lower Chance	22.1	15.1	62.8	6.9	15.5	77.7
30% Lower Chance	4.7	22.1	73.3	1.5	8	90.5

Age, gender, socio-economic status, education, and political ideology did not impact ventilator allocation attitudes. Those participants that strongly endorsed ventilator allocations based on chances of survival in the scenarios without any additional risk factors were not more or less likely to reject ventilator allocation based on race ($r = -0.047$).

Race of participants did not affect vaccine allocation. In the US, (but not the UK) gender ($F3,661) = 7.238$, $P < .0001$) and social political orientation $F(1,661) = 11.388$, $P = 0.001$) impacted vaccine allocation based on race. Male participants had a less positive attitude (P =

For peer review only - http://bmjopen.bmj.com/site/about/guidelines.xhtml

0.003) and more socially conservative participants gave less weight to race in vaccine allocation (parameter estimate = - 0.305, 95% CI = -.480, -.130).

Influence of attitudes towards race on prioritisation

In both countries, participants were more likely to believe that adverse COVID-19 related health outcomes were the result of injustice and outside the individual's control for race than for obesity/male sex [Supplementary Figure 2]. Obesity was associated with the strongest sense of individuals' health outcomes being within their control. US and UK respondents had similar attitudes towards the respective racial minorities [SM].

In the US and the UK, responses on the Modern Racism Scale were associated with attitudes towards ventilator and vaccine allocation based on race (Ventilator: US: F(1,634) = 10.73, p = 0.001, UK: F(1,624) = 13.88, P < .0001; Vaccine: US: F(1,621 =23.00, P <0.0001, UK: F(1,607)=16.98, P <0.0001). The more participants endorsed modern racism statements, the less positive their composite attitudes towards ventilator or vaccine allocation to racial minority patients. A minority (27.3%) of participants had a mean score of higher than three on the modern racism scale, indicating an endorsement of modern racism. These participants did not give weight to race in ventilator or vaccine allocations [Supplementary Figure 3]. Participants with low modern racism scores also did not give weight to race in ventilator allocation but did for vaccines. Modern racism did not affect prioritisation based on sex/obesity [Supplementary Figure 3]. Perceptions of injustice did not predict ventilator or vaccine allocation attitudes in the US (F(1,634) = 0.778, P = 0.378) or the UK (F(1,624) = 2.732, P = 0.099).

Discussion

Previous research has identified some of the public's prioritisation preferences in relation to race and vaccines.[24] To our knowledge, this is the first study to examine the views of the public on race in ventilator allocation and in comparison with other factors. This international study was conducted during the second wave of the COVID-19 pandemic in two countries with documented and publicised adverse outcomes for racial minorities. Survey participants largely allocated ventilators and vaccines on the basis of medical factors and independent of the race of recipients. They were highly sensitive to small differences in predicted outcome. For example, for a 10% difference in predicted survival, 71.9% of participants gave the ventilator to the patient with higher chances while 82.7% chose to toss a coin toss where there was no difference in predicted chance. For vaccine allocation we found similar results for even smaller differences in risk of severe Covid (5% difference). Few respondents in our survey, even those from racial minorities themselves, endorsed priority for ventilators for those from racial minorities. However, where other factors were equal, 40-60% of respondents allocated vaccines to patients/groups with additional risk based on race, sex, or obesity. In the survey, willingness to prioritise based on additional risk did not correlate with participants' perceptions of whether risk factors were caused by social injustice. However, covert racial attitudes (endorsed by approximately a quarter of respondents) negatively impacted attitudes to allocation in both countries.

The different weight given to race in the allocation of vaccines versus ventilators in our study may reflect people's desire to prevent the most deaths.[7,32] Previous research has shown strong

For peer review only - http://bmjopen.bmj.com/site/about/guidelines.xhtml

public endorsement for prioritising chances of survival in ventilator allocation and probability of severe illness for vaccine allocation.[24,28,33–35] However, whereas vaccines are provided to prevent illness, ventilators are provided to those already critically ill. Participants might have felt that allocating ventilators preferentially to those with additional risk factors (race, sex, obesity) could lead to more deaths from COVID-19 if such patients (even with otherwise equal medical risk) have higher mortality rates. Another possible explanation, at least for US participants, is that some were aware of more recent evidence of similar hospitalised critical care outcomes for Black patients.[36] This may have led some to conclude that since hospital admissions were in proportion to the occurrence of severe disease in the community, allocation of critical care resources preferentially would not be justified.[37] (Since the survey was conducted prior to much of this evidence emerging, this was potentially less likely to influence responses). Respondents might have regarded preferential allocation of a life-saving intervention like ventilators as more legally or ethically questionable than prioritisation of vaccines. Finally, participants might have reasoned that ventilators do not have wider community benefits, but vaccines do. Hence, preferential allocation of vaccines represents a more substantial (and possibly less controversial) means to reduce inequality.

However, in our study participants who expressed a strong attitude for ventilator allocation based on chances of survival were not more or less likely to reject preference in ventilator allocation based on race. Other ethical principles or considerations may be guiding allocation decisions, for example concern for equality and avoiding discrimination.[38,39] Respondents did not believe that racial minorities should be at a disadvantage for access to ventilators – reinforcing the importance of ensuring that medical criteria used for allocation are not racially biased.[40,41]

One reason to potentially give priority in allocation of scarce treatment to racial minorities is because of concerns over past or present injustice.[12] In our survey, UK and US respondents perceived higher death rates among minorities to be more unjust and less under individual control than the mortality rates of male and obese recipients. However, these perceptions did not affect allocation decisions. Similarly, political orientation had little impact on allocation decisions in our survey. More conservative respondents were less inclined to indicate that injustice is responsible for higher death rates. However, this perception did not affect allocation decisions.

Allocation decisions in our survey were associated with responses on the modern racism scale. This instrument is intended to capture covert racial attitudes.[30] While people who score high on the modern racism scale potentially do not perceive themselves as racist, discriminatory attitudes can nevertheless guide their decisions. Modern racism might measure negative attitude towards affirmative action policies in general. However, in our survey, modern racism did not predict attitudes to sex or obesity in allocation. Furthermore, in our models, political ideology was not associated with allocation based on race. Taken together, these findings suggest that some opposition to giving weight to race in prioritisation arises from covert racial attitudes. However, even those respondents with low scores on the modern racism scale (three quarters of those surveyed) did not on average give weight to race in allocation of ventilators. Covert racial attitudes do not appear to explain our overall finding of the lack of weight given to race in public attitudes to ventilator allocation.

Importantly our results help contextualise previous findings that looked at race in allocation in isolation. For example, a US Gallup/COVID Collaborative survey found that 74-85% respondents supported giving priority for vaccine access to Black, Hispanic and Native

For peer review only - http://bmjopen.bmj.com/site/about/guidelines.xhtml

Americans.[24] In our study, slightly less than half of respondents endorsed this, but only in cases where other risk factors were equal. Our findings mirror recent ones about affirmative action in higher education, where Americans have positive attitudes towards affirmative action in general, but also feel strongly that race should not trump other admission criteria such as grades.[42] This highlights the importance of clarifying the weight given to different ethical values when surveying the public's views on resource allocation, but also points to the importance of alternative approaches to lessening the impact of Covid-19 on racial minorities.

Strengths and limitations

The hypothetical scenarios used in our study enabled us to assess and compare the attitude of the public towards allocation of two key resources in a pandemic. The use of parallel examples enabled appraisal of the relative ethical weight given to some important risk factors compared with the risk of serious illness or death. Scenarios were highly specific, somewhat artificial, and clinically unrealistic (particularly in relation to small predicted differences between patients or groups). This could have differed from participants' lived experience and may have contributed to unease in prioritisation decisions.

Survey responses allowed us to quantify some elements of the public's values, but we were not able to assess the reasons why particular answers were given. Future research should explore this.

The survey was conducted during the COVID-19 pandemic at a fraught and emotional time. This would have increased the salience of the survey for participants, and could provide a relevant snapshot of community values at that point in time. However, responses might have been different if the survey had been conducted at a different time. For example, respondents might have been sensitive to the controversial nature of the topic. They may have been more likely to advocate for correcting historical injustices if the survey were conducted outside a pandemic.

One key limitation is that despite our attempt to obtain a nationally representative sample, in our US sample, Hispanic and Black participants were underrepresented. Thus, our study might underestimate how different racial groups feel about allocation of scarce resources. Furthermore, although we aimed to create and word questions in a neutral and inclusive way, the involvement of Black and BAME community members would have been beneficial in the survey design.

Conclusion

Obviously, the results of this survey do not settle the ethical question of whether governments should prioritise based on race or other forms of social disadvantage. Ethical argument and analysis may lead to prioritisation policies that differ from those endorsed by the majority. However, understanding the general approach of the public towards prioritisation in settings of severe scarcity may be important in debate and deliberation: it may challenge or confirm assumptions about what the public would or would not support, as well as highlight areas of ethical consensus or point to important areas of disagreement.[43] Our survey identified that in

For peer review only - http://bmjopen.bmj.com/site/about/guidelines.xhtml

specific allocation scenarios involving high demand and severe resource shortage, a majority of US and UK respondents chose to allocate ventilators to patients with predicted higher chance of survival and vaccines to those at higher risk of severe illness, regardless of patient race. A very small proportion of respondents (<15%) prioritised ventilator allocation to patients from a racial minority, and this was higher among UK respondents from ethnic minority backgrounds. We also found support from a large minority for inclusion of race as a tie-break consideration in vaccine allocation. Although we may hope that it is never needed, these findings may be relevant to planning for the next pandemic.

Contribution statement: AK, HZ, and DW designed the survey, JS, IS, and WS-A provided crucial feedback on methodology and design. HZ organised the collection of the data. AK analyzed the data. AK, HZ, and DW wrote the manuscript, JS, IS, and WS-A provided crucial feedback.

Competing interests: The authors declare that they have no conflicts of interest.

Ethics approval: The study was approved by the University of Oxford Central University Research Ethics Committee [R73841/RE002]. All participants involved provided informed consent.

Funding:
This study (survey costs) was commissioned and paid for by the World Health Organization (WHO). Copyright in the original work on which this article is based belongs to WHO. The authors have been given permission to publish this article.

Researchers were funded in part, by the Wellcome Trust [203132/Z/16/Z] (DW, HZ) and by the Arts and Humanities Research Council (AHRC) (DW) as part of the UK Research and Innovation rapid response to COVID-19 [AH/V013947/1], as well as the NIHR Oxford Health Biomedical Research Centre (IS-BRC-1215-20005) (IS). The funders had no role in the preparation of this manuscript or the decision to submit for publication. For the purpose of open access, the authors have applied a CC BY public copyright license to any Author Accepted Manuscript version arising from this submission.

For peer review only

For peer review only - http://bmjopen.bmj.com/site/about/guidelines.xhtml

References

1. Wilkinson D. ICU triage in an impending crisis: uncertainty, pre-emption and preparation. *J Med Ethics*. 2020;46(5):287-288. doi:10.1136/medethics-2020-106226

2. Verelst F, Kuylen E, Beutels P. Indications for healthcare surge capacity in European countries facing an exponential increase in coronavirus disease (COVID-19) cases, March 2020. *Eurosurveillance*. 2020;25(13):2000323. doi:10.2807/1560-7917.ES.2020.25.13.2000323

3. Cylus J, Panteli D, van Ginneken E. Who should be vaccinated first? Comparing vaccine prioritization strategies in Israel and European countries using the Covid-19 Health System Response Monitor. *Isr J Health Policy Res*. 2021;10(1):16. doi:10.1186/s13584-021-00453-1

4. Emanuel EJ, Persad G, Kern A, et al. An ethical framework for global vaccine allocation. *Science*. 2020;369(6509):1309-1312. doi:10.1126/science.abe2803

5. WHO SAGE Roadmap For Prioritizing Uses Of COVID-19 Vaccines In The Context Of Limited Supply. Accessed July 15, 2021. https://www.who.int/publications-detail-redirect/who-sage-roadmap-for-prioritizing-uses-of-covid-19-vaccines-in-the-context-of-limited-supply

6. Bubar KM, Reinholt K, Kissler SM, et al. Model-informed COVID-19 vaccine prioritization strategies by age and serostatus. *Science*. 2021;371(6532):916-921. doi:10.1126/science.abe6959

7. Ehni HJ, Wiesing U, Ranisch R. Saving the most lives—A comparison of European triage guidelines in the context of the COVID-19 pandemic. *Bioethics*. 2021;35(2):125-134. doi:10.1111/bioe.12836

8. Savulescu J, Vergano M, Craxì L, Wilkinson D. An ethical algorithm for rationing life-sustaining treatment during the COVID-19 pandemic. *BJA Br J Anaesth*. 2020;125(3):253-258. doi:10.1016/j.bja.2020.05.028

9. Emanuel EJ, Persad G, Upshur R, et al. Fair Allocation of Scarce Medical Resources in the Time of Covid-19. *N Engl J Med*. 2020;382(21):2049-2055. doi:10.1056/NEJMsb2005114

10. Giubilini A, Savulescu J, Wilkinson D. Queue questions: Ethics of COVID-19 vaccine prioritization. *Bioethics*. 2021;35(4):348-355. doi:10.1111/bioe.12858

11. Bibbins-Domingo K, Petersen M, Havlir D. Taking Vaccine to Where the Virus Is—Equity and Effectiveness in Coronavirus Vaccinations. *JAMA Health Forum*. 2021;2(2):e210213-e210213. doi:10.1001/jamahealthforum.2021.0213

12. Schmidt H, Roberts DE, Eneanya ND. Rationing, racism and justice: advancing the debate around ‘colourblind’ COVID-19 ventilator allocation. *J Med Ethics*. Published online January 6, 2021:medethics-2020-106856. doi:10.1136/medethics-2020-106856

For peer review only - http://bmjopen.bmj.com/site/about/guidelines.xhtml

13. Mathur R, Bear L, Khunti K, Eggo RM. Urgent actions and policies needed to address COVID-19 among UK ethnic minorities. *The Lancet*. 2020;396(10266):1866-1868. doi:10.1016/S0140-6736(20)32465-X

14. White DB, Lo B. Mitigating Inequities and Saving Lives with ICU Triage during the COVID-19 Pandemic. *Am J Respir Crit Care Med*. 2021;203(3):287-295. doi:10.1164/rccm.202010-3809CP

15. Tai DBG, Shah A, Doubeni CA, Sia IG, Wieland ML. The Disproportionate Impact of COVID-19 on Racial and Ethnic Minorities in the United States. *Clin Infect Dis*. 2021;72(4):703-706. doi:10.1093/cid/ciaa815

16. Morales DR, Ali SN. COVID-19 and disparities affecting ethnic minorities. *The Lancet*. 2021;397(10286):1684-1685. doi:10.1016/S0140-6736(21)00949-1

17. Who Gets a Vaccine First? U.S. Considers Race in Coronavirus Plans - The New York Times. Accessed June 24, 2021. https://www.nytimes.com/2020/07/09/us/coronavirus-vaccine.html

18. Jagannathan M. Should Black and Latino people get priority access to a COVID-19 vaccine? MarketWatch. Accessed June 24, 2021. https://www.marketwatch.com/story/should-black-and-latino-people-get-priority-access-to-a-covid-19-vaccine-2020-07-16

19. Sederstrom N. The "Give Back": Is There Room For It? Bioethics.net. Published February 23, 2021. http://www.bioethics.net/2020/07/the-give-back-is-there-room-for-it/

20. Kaposy C, Bandrauk N. Prioritizing Vaccine Access for Vulnerable but Stigmatized Groups. *Public Health Ethics*. 2012;5(3):283-295. doi:10.1093/phe/phs010

21. Schmidt H, Gostin LO, Williams MA. Is It Lawful and Ethical to Prioritize Racial Minorities for COVID-19 Vaccines? *JAMA*. Published online October 14, 2020. doi:10.1001/jama.2020.20571

22. Opinion | Four Ways to Fix the Vaccine Rollout. *The New York Times*. https://www.nytimes.com/2021/01/07/opinion/coronavirus-vaccine-distribution.html. Published January 7, 2021. Accessed July 15, 2021.

23. Essay: Distributive justice during a pandemic – WHYY. Accessed June 24, 2021. https://whyy.org/articles/a-disturbing-medical-consensus-is-growing-heres-what-it-could-mean-for-black-patients-with-coronavirus/

24. Persad G, Emanuel EJ, Sangenito S, Glickman A, Phillips S, Largent EA. Public Perspectives on COVID-19 Vaccine Prioritization. *JAMA Netw Open*. 2021;4(4):e217943. doi:10.1001/jamanetworkopen.2021.7943

25. Williamson EJ, Walker AJ, Bhaskaran K, et al. Factors associated with COVID-19-related death using OpenSAFELY. *Nature*. 2020;584(7821):430-436. doi:10.1038/s41586-020-2521-4

For peer review only

26. [Dataset] Kappes A, Zohny H, Savulescu J, Singh I, Sinnot-Armstrong W, Wilkinson D. Data from: Race and resource allocation: An online survey of US and UK adults' attitudes towards COVID-19. Published online June 1, 2022. osf.io/mhyqz

27. U.S. Census Bureau QuickFacts: United States. Accessed July 22, 2021. https://www.census.gov/quickfacts/fact/table/US/PST045219

28. Wilkinson D, Zohny H, Kappes A, Sinnott-Armstrong W, Savulescu J. Which factors should be included in triage? An online survey of the attitudes of the UK general public to pandemic triage dilemmas. *BMJ Open*. 2020;10(12):e045593. doi:10.1136/bmjopen-2020-045593

29. Butcher B, Massey J. Why are more people from BAME backgrounds dying from coronavirus? *BBC News*. https://www.bbc.com/news/uk-52219070. Published June 19, 2020. Accessed January 31, 2022.

30. McConahay JB. Modern racism, ambivalence, and the Modern Racism Scale. In: J. F. Dovidio, S. L. Gaertner, eds. *Prejudice, Discrimination, and Racism*. Academic Press; 1986:91-125.

31. Morrison T, Kiss M. Modern Racism Scale. In: Zeigler-Hill V, Shackelford E, eds. *Encyclopedia of Personality and Individual Differences*. Springer International Publishing; 2017.

32. Savulescu J, Persson I, Wilkinson D. Utilitarianism and the pandemic. *Bioethics*. 2020;34(6):620-632. doi:10.1111/bioe.12771

33. Buckwalter W, Peterson A. Public attitudes toward allocating scarce resources in the COVID-19 pandemic. *PLOS ONE*. 2020;15(11):e0240651. doi:10.1371/journal.pone.0240651

34. Grover S, McClelland A, Furnham A. Preferences for scarce medical resource allocation: Differences between experts and the general public and implications for the COVID-19 pandemic. *Br J Health Psychol*. Published online June 20, 2020:e12439. doi:10.1111/bjhp.12439

35. Duch R, Roope LSJ, Violato M, et al. Citizens from 13 countries share similar preferences for COVID-19 vaccine allocation priorities. *Proc Natl Acad Sci*. 2021;118(38). doi:10.1073/pnas.2026382118

36. Song Z, Zhang X, Patterson LJ, Barnes CL, Haas DA. Racial and Ethnic Disparities in Hospitalization Outcomes Among Medicare Beneficiaries During the COVID-19 Pandemic. *JAMA Health Forum*. 2021;2(12):e214223. doi:10.1001/jamahealthforum.2021.4223

37. Hodge JG, Hanfling D, Hick JL, Piatt JL. Diminishing disparities in U.S. crisis standards of care: Medical and legal challenges. *eClinicalMedicine*. 2021;34. doi:10.1016/j.eclinm.2021.100838

38. Crisp by R. Equality, Priority, and Compassion. *Ethics*. 2003;113(4):745-763. doi:10.1086/373954

For peer review only - http://bmjopen.bmj.com/site/about/guidelines.xhtml

39. Kamm FM. Equal Treatment and Equal Chances. *Philos Public Aff.* 1985;14(2):177-194.

40. Ashana DC, Anesi GL, Liu VX, et al. Equitably Allocating Resources during Crises: Racial Differences in Mortality Prediction Models. *Am J Respir Crit Care Med.* 2021;204(2):178-186. doi:10.1164/rccm.202012-4383OC

41. Sjoding MW, Dickson RP, Iwashyna TJ, Gay SE, Valley TS. Racial Bias in Pulse Oximetry Measurement. *N Engl J Med.* 2020;383(25):2477-2478. doi:10.1056/NEJMc2029240

42. Inc G. The Harvard Affirmative Action Case and Public Opinion. Gallup.com. Published October 22, 2018. Accessed January 31, 2022. https://news.gallup.com/opinion/polling-matters/243965/harvard-affirmative-action-case-public-opinion.aspx

43. Savulescu J, Gyngell C, Kahane G. Collective Reflective Equilibrium in Practice (CREP) and controversial novel technologies. *Bioethics*. Published online 2021. doi:10.1111/bioe.12869

Figure legends

Figure 1. Survey Design. Participants from the US (N = 1296) or the UK (N = 1284) answered questions about their preferences for allocating scarce medical resources (online sample, aiming to be nationally representative for sex, age, race, household income, and employment – see Supplemental Methods). Fourteen percent of the UK sample, and 19% of the US sample described their racial background as non-White. A: Half the participants (N = 1262) saw scenarios relating to ventilators. They had to decide which of two potential recipients should get the ventilator. Potential recipients had different chances of surviving COVID-19 with ventilation (40%, 50%, or 70%) and one of them had an additional risk factor. Participants could give it to either one of the potential recipients or choose to toss a coin to decide. B: The other half of participants (N = 1318) saw scenarios relating to vaccines. Here, they had to decide which of two potential recipients should get the vaccine. Potential recipients had different chances of developing severe COVID-19 (10%, 15%, or 20%) and one of them had an additional risk factor. Again, participants could give it to either one of the two potential recipients or toss a coin. C: Potential recipients were either individual recipients or groups of recipients. D) Additional risk factors used in our study were racial minority status (Black in the US, BAME in the UK), being male, or being obese. The opposite additional risk factors were white, female, and healthy weight, respectively.

Figure 2. The role of chances of developing severe COVID-19 for vaccine allocation and chances of survival for ventilator allocations in the absence of additional risk factors. Participants saw either scenarios that involved individual patients (A,C) or groups of patients (B,D). For both individual patients and groups of patients, the greater the difference in chances of survival, the more participants were willing to allocate the ventilator to the potential recipient more likely to survive (A,B). Similarly, the larger the difference in chances of developing severe COVID-19 if infected, the more participants were willing to allocate the vaccine to the potential recipient at higher risk (C,D). (Error bars = 95% Confidence Interval) Note that, in these scenarios (involving only medical risk factors), we gave participants a wider range of outcome variation between recipients than in later scenarios involving additional risk factors. (For symbols, see Fig 1)

Figure 3. (A-C) Preferences for and attitudes towards prioritization of ventilators. A: Percentages of respondents willing to give the ventilator to the racial minority/male/obese patient when both patients had either a similar, or a 10% or 20% higher or lower survival chance. For comparison, we added the responses for scenarios without additional risk factors (white bars, labelled "survival chances"). B: For illustrative purposes, we subtracted the percentage of answers on the scenarios without additional risk factors from those with them. Race and sex did not elicit robust preferences, while obesity elicited negative prioritization preferences. C: Our composite attitude score mirrored the outlined findings. (**D-F) Preferences for and attitudes towards**

For peer review only - http://bmjopen.bmj.com/site/about/guidelines.xhtml

prioritization of vaccines. D: Percentages of respondents willing to give the vaccine to the recipient with an additional risk factor when their chances of developing severe COVID-19 were either the same or were 5% or 10% higher or lower than the recipient without an additional risk factor. For comparison, we added the answers for scenarios without additional risk factors (White bars: labelled "Covid risk"). E: For illustrative purposes, we subtracted the percentage of answers on the scenarios without additional risk factors from those that contain them. A clear positive preference for each factor emerged, with obesity eliciting the strongest positive responses. F: Our composite attitude score mirrored the outlined finding. For vaccines, each factor elicited a positive preference, obesity being strongest. (Error bars = 95% Confidence Interval)

For peer review only

For peer review only - http://bmjopen.bmj.com/site/about/guidelines.xhtml

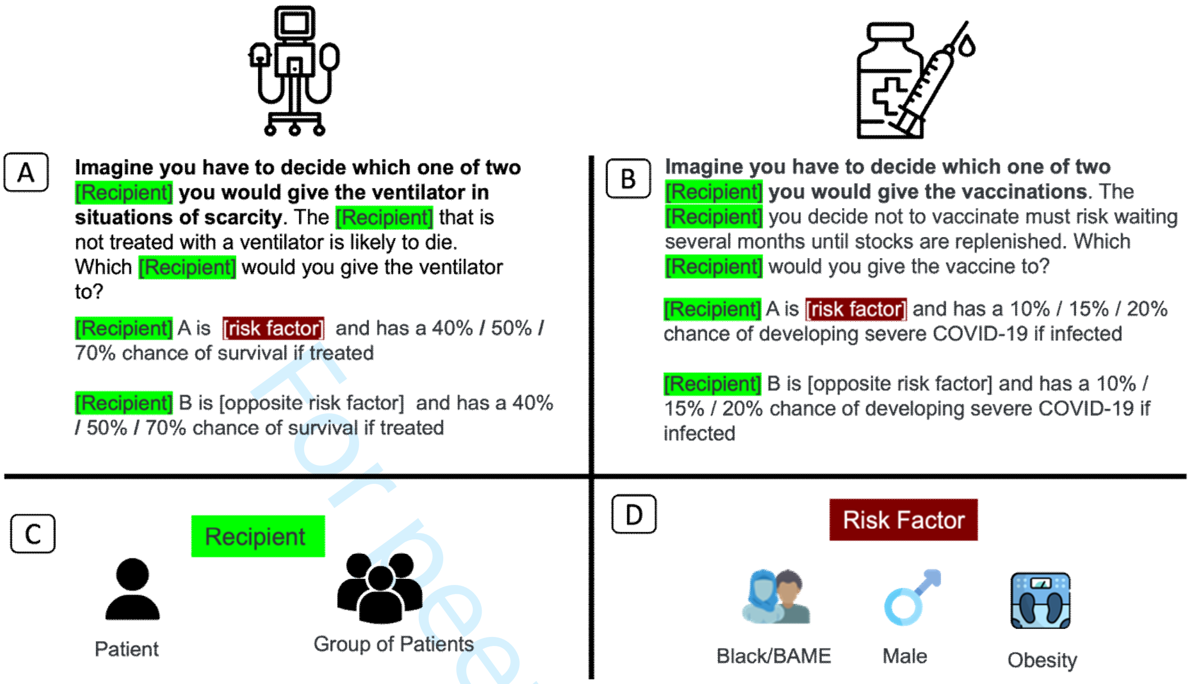

For peer review only

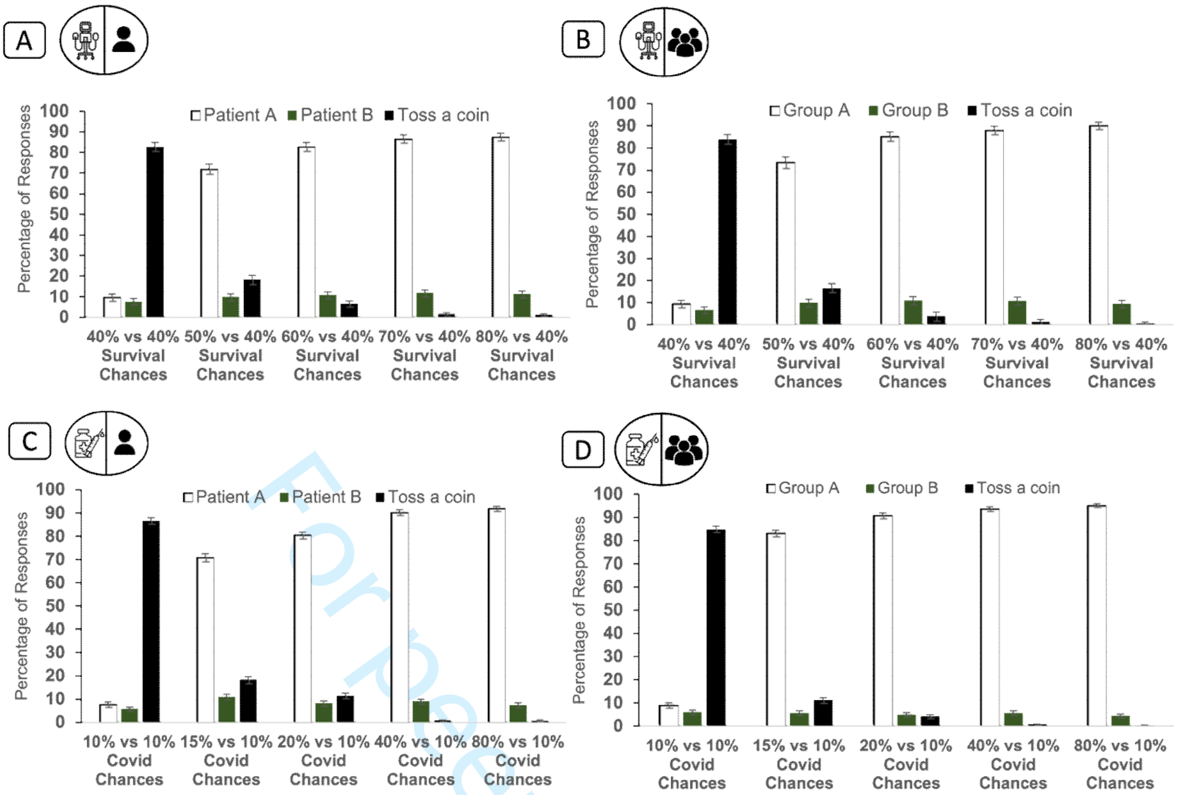

For peer review only

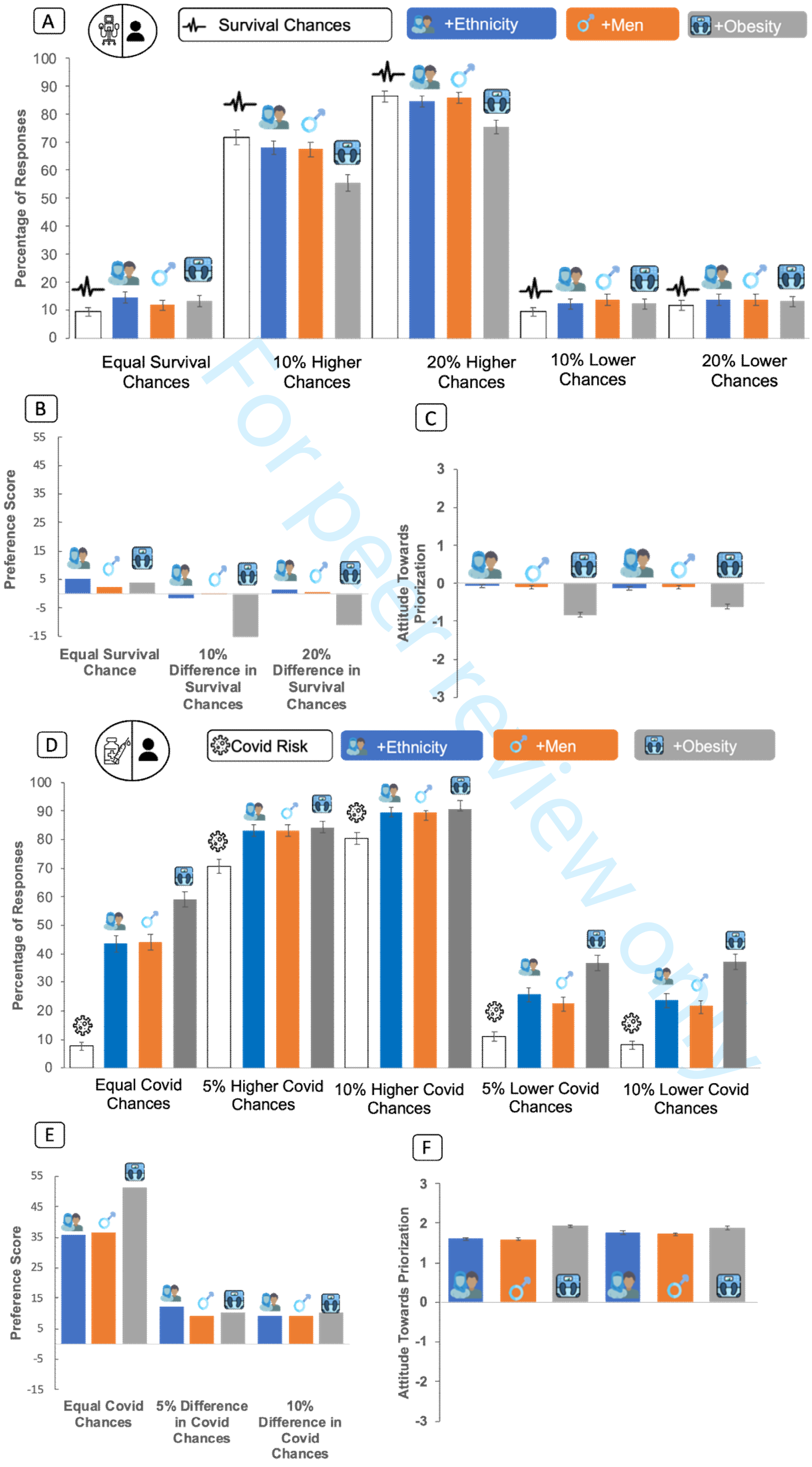

For peer review only

For peer review only

Supplemental Materials: Public attitudes towards race and resource allocation in a pandemic

Andreas Kappes[1], Hazem Zohny[2], Julian Savulescu[2,3], Ilina Singh[4 5], Walter Sinnott-Armstrong[6], Dominic Wilkinson[2,3,7]

Affiliations:
[1]City, University of London
[2] Oxford Uehiro Centre for Practical Ethics, University of Oxford
[3] Murdoch Children's Research Institute, Melbourne, Australia.
[4] Wellcome Trust Centre for Ethics and the Humanities, University of Oxford
[5] Department of Psychiatry, University of Oxford
[6] Kenan Institute for Ethics and Philosophy Department, Duke University
[7] John Radcliffe Hospital, Oxford, UK

Corresponding author Email: hazem.zohny@philosophy.ox.ac.uk

For peer review only

For peer review only - http://bmjopen.bmj.com/site/about/guidelines.xhtml

Supplemental Methods

Procedure

The survey was designed to assess participants' preferences for using medical factors as well as race, sex, obesity for allocating vaccines to people in need. Two surveys were conducted, one in the US and one in the UK. The surveys were adapted from a previous survey (4) on triage guidance involving ventilator allocations. Full survey materials can be found here: osf.io/w7bpq.

Vaccine Allocations

All participants were first introduced to the context of the allocation decisions. We wanted to make sure that all participants knew what would happen to the recipient that would not get vaccine, that all recipients wanted to get the vaccine, and that the vaccine was effective and safe. Participants read:

> *For the following scenarios, imagine you are working in a clinic. You are in charge of deciding which patients should get vaccinated first against COVID-19. You have one remaining dose of the vaccine left, and you must choose between two patients. The unvaccinated patient must wait several months before stocks are replenished. For the purpose of this survey, please think of the vaccine as if it had a 100% success rate in preventing COVID-19 and no side effects whatsoever. And each person under consideration wants to get vaccinated.*

Participant then learned that the estimate for each recipient's chance of getting severe COVID-19 was high and was based on their age and medical history. Furthermore, they learned that their task was to decide whom to give the vaccine.

Risk of developing severe COVID-19. To determine how much weight participants would give the risk of developing severe COVID-19 for vaccine allocations, participants answered five scenarios in which the potential recipients only differed in their chance of developing severe COVID-19. In one scenario, one patient had an 80% chance and the other one a 10% chance of developing severe COVID-19 (other scenarios: 40% versus 10%, 20% versus 10%, 15% versus 10% and 10% versus 10%). Thereafter, participants were randomly introduced to one of the three additional factors: race, sex, and obesity.

Racial minority scenarios. For a racial minority in the US, we chose Black recipients and, in the UK, BAME recipients. BAME is an umbrella term, common in the United Kingdom, used to describe non-white racial groups. While a contested term, it was most consistently used in media reports on the pandemic in the UK (e.g., 5). Before participants had to decide if they wanted to give the vaccine to the member of the racial minority, we ensured that all participants were aware of the inequality in COVID-related deaths in the UK and in the US. UK participants read: "*According to the Office for National Statistics, individuals belonging to Black, Asian, and minority ethnic (BAME) groups have in general been shown to die from COVID-19 at a higher rate than that of White individuals.*" US participant read: "*According to the Centre for Disease Control and Prevention, Black Americans have been shown to die from COVID-19 at a higher*

For peer review only - http://bmjopen.bmj.com/site/about/guidelines.xhtml

rate than of White Americans." Thereafter, participants worked on five scenarios. All of these scenarios involved one Black (BAME) and one White patient. In one scenario, both patients had the same risk of illness (10% versus 10%), in two scenarios the Black (BAME) patient had a higher risk of severe COVID-19 (5% and 10% higher), and in two scenarios the White patient a higher risk (5% and 10% higher). Participants indicated for each whether they wanted to give the vaccine to the racial minority patient, the white patient, or toss a coin.

Sex and obesity scenarios. Participants saw a similar set of scenarios for sex-based and obesity-based allocation decisions. Before working on the scenarios that involved men and women, participants read: "*According to scientific studies, men have been shown to die from COVID-19 at a higher rate than that of women."* The rationale was to inform participants that being male may be predictive of a worse outcome or survival chance if infected with COVID-19 (6). And before working on the scenarios involving obesity, participants read: "*According to scientific studies, people who are obese (severely overweight) have been shown in general to die from COVID-19 at a higher rate than that of people who have a healthy weight."* The rationale for presenting participants with this was to inform them about obesity being predictive of a worse outcome or survival chance if infected with COVID-19 (7). The five obesity-related and five sex-related scenarios followed the exact same structure as the race-related scenarios.

Group scenarios. To test if it would make a difference if participants considered only one patient, or a group of patients, we repeated the same set of scenarios for race, sex, and obesity for groups of patients. The reason for including group scenarios was twofold. First, there is a large body of evidence that suggest that people have different moral intuitions when they think about one persons versus a group of people in need (8). Second, one might argue that people might feel that the single recipient scenario for vaccine distributions is unrealistic since such decisions most often are made for large groups, rather than individuals. To ensure that such perceptions did not impact allocation preferences, we additionally included group scenarios. The scenarios followed the exact same structure as the individual ones but instead of deciding between two patients, participants had to decide between two groups of patients. As an introduction to this section of the survey, participants were told:

> *Imagine you are a policymaker for the government. At the moment, the number of vaccines available is limited and you do not have enough to protect everyone. Sometimes, policy makers face difficult decisions about which subsets of the population should be given a vaccine and which shouldn't. You will make decisions about whether or not to provide vaccines to one of two groups of people. If the people in this survey are not vaccinated, they are likely to get COVID-19 and suffer serious health consequences as a result. The group you decide not to vaccinate must risk waiting several months until stocks are replenished.*

Race, sex, and obesity scenarios. The structure of each of these scenario groups followed the structure for the respective vaccine scenario. Participants were informed about the effect race, sex, and obesity had on COVID-related deaths before working on the corresponding set of scenarios (see above). In the five race-related scenarios, participants both patients had the same chances of survival (40% versus 40%), in two scenarios the Black (BAME) patient had a higher chance of survival (10% and 20% higher), and in two scenarios the White patient a higher risk

chance of survival (10% and 20% higher). Participants indicated for each whether they wanted to give the vaccine to the racial minority patient, the white patient, or toss a coin. The scenarios related to sex and obesity followed the exact structure as the race-related scenarios.

Prioritization Attitude Measure. For each of the sets of scenarios, we created an attitude score by counting the number of times participants decided to give the vaccine to the person with an additional risk factor on each of the five respective scenarios. For instance, a participant that would always give the vaccine to the recipient from the racial minority would have an attitude score of 5, a participant who would allocate solely by the risk of contracting severe COVID-19 would get a 0, and a person that would always give priority to the white recipient would get a score of -5. We did this for all the patient and all the group scenarios.

Perceptions of Injustice, Responsibility, Stereotypes, and Racism

After participants worked on the scenarios, we asked them a series of questions to capture their perceptions of racial minorities, men, and obese people.

Perceptions of injustice, responsibility. To measure as how unjust people perceived the higher COVID-related death rates, we asked participants to indicate their agreement with the following statement: "Injustice was the main reason [category] individuals died more often than individuals from other groups". To measure perceptions of responsibility of the group for the higher death rates, we asked participants to indicate their agreement with the following statement: "The main reason [category] die more often from COVID-19 is due to factors outside their control." For both questions, scales ranged from 1 (strongly disagree) to 5 (strongly agree).

Stereotypes. According to the stereotype content model, perceptions of groups can be described along two fundamental dimensions: warmth and competence (9, 10). In line with previous research, we measured warmth by how warmly participants felt towards black (US) or BAME (UK) people, men, and obese people. We measured competence by asking participants how competent they perceived black (US) or BAME (UK) people, men, and obese people to be. We used 100-point scales for participants to indicate their perceptions.

Covert Racism - Modern Racism Scale. The modern racism scale is intended to capture subtle or covert racial attitudes or the endorsement of the idea that racial minorities are getting undeserved attention (1). Although modern racist potentially do not perceive themselves as racist, attitudes guide their decisions in situations that provide nonracially motivated reasons. Hence, the modern racism scale provides seven statements that one could endorse or reject (e.g., "Discrimination against Blacks is no longer a problem in the United States" and "Over the past few years, Blacks have gotten more economically than they deserve"). Participants are asked to express their agreement of each statement via a 5-point scale, ranging from 1 (Strongly disagree) to 5 (Strongly agree). Extensive previous research has found that scores on the modern racism scale correlate with anti-Black affect, right-wing authoritarianism, social dominance orientation, and indices of old-fashioned racism (11).

Statistical Analyses (Fixed Linear Models)

For peer review only

We ran a series of 4 linear models to explore the role of various factors in predicting racial prioritization attitudes. For each model, we defined attitudes as dependent variable and subjects as the random factor. In the first two models, we entered age, gender, race, relative income, educational level, political ideology (general, social, and economical), and type of scenario (patient versus group-based) as fixed factors. In the second two models, we additionally added perceptions of injustice, responsibility, warmth, competence, and modern racism scores (see results above).

Supplemental Results

1. Group Preferences for Vaccine and Ventilator Preferences

Patterns of response were similar for scenarios involving groups as for individual patients (Supplementary Figure 2 for individual preferences, Supplementary Figure 1 for group preferences).

For peer review only

For peer review only - http://bmjopen.bmj.com/site/about/guidelines.xhtml

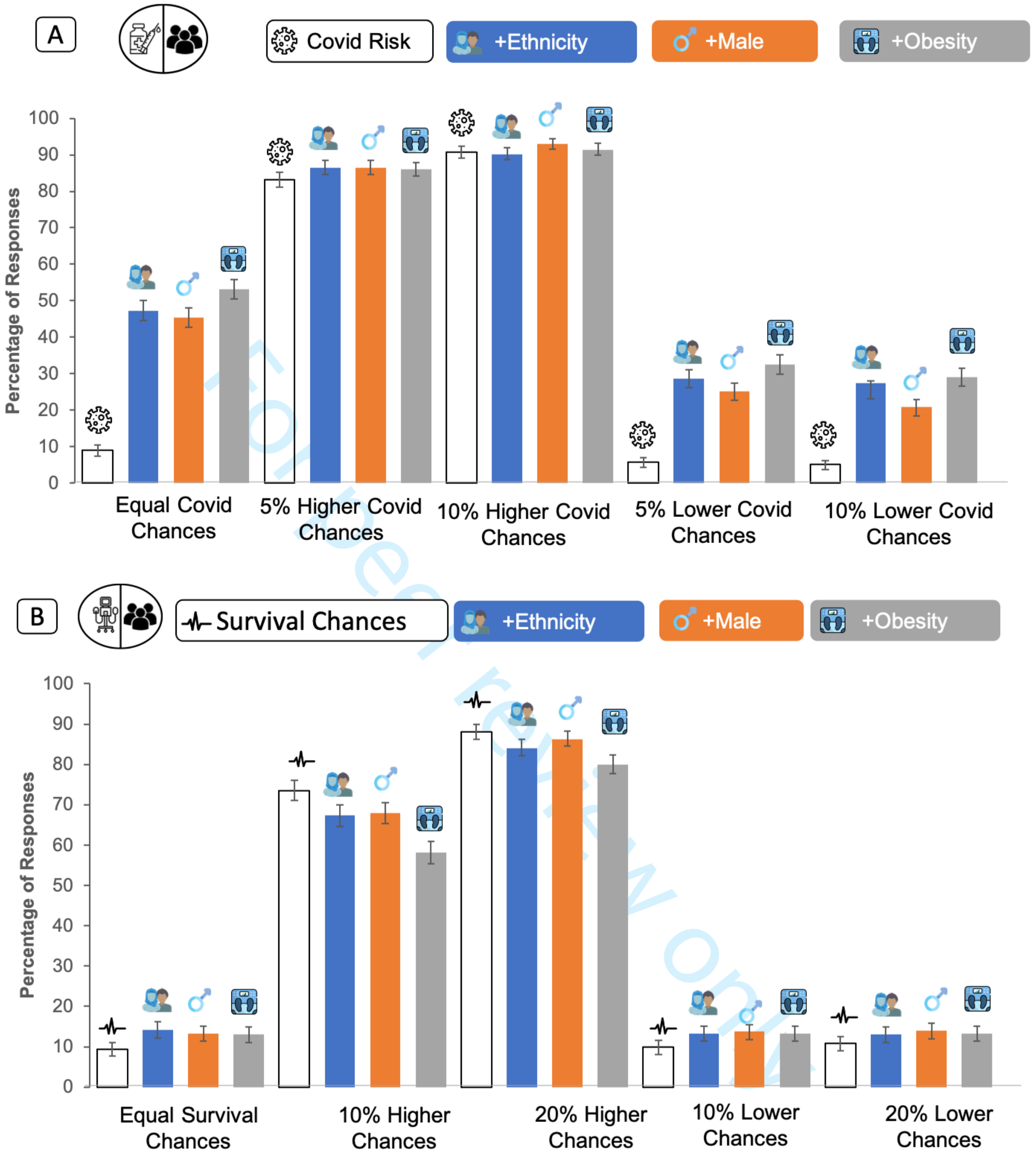

Supplementary Figure 1. A: Preferences for prioritization of vaccines. Response to scenarios where competing individual patients differed in medical risk alone or in medical risk plus an additional risk factor (race, sex, or obesity), where the patient with the additional risk factor had a 5% or 10% higher or lower chance of getting severe Covid based on medical risk alone. The bars indicate the proportion of respondents who would allocate the vaccine to a patient with a particular characteristic and risk profile. **B: Preferences for prioritization of ventilators.** A: Response to scenarios where competing individual patients differed in medical risk alone (Equal COVID chance), or in medical risk plus an additional risk factor (race, sex, or obesity), where the patient with the additional risk factor had a 10% or 20% higher or lower chance of dying based on medical risk alone. The bars indicate the proportion of respondents who would allocate the ventilator to a patient with a particular characteristic and risk profile.

For peer review only - http://bmjopen.bmj.com/site/about/guidelines.xhtml

2. General Linear Mixed Models

To test which variables impacted participants' attitudes towards risk factors, we ran a series of four linear models to explore the role of various factors in predicting racial prioritization attitudes. For each model, we defined attitudes as dependent variable and subjects as the random factor. In the first two models, we entered age, gender, race, relative income, educational level, political ideology (general, social, and economical), and type of scenario (patient versus group-based) as fixed factors. In the second two models, we additionally added perceptions of injustice, responsibility, warmth, competence, and modern racism scores. The full results of each model can be seen in Supplementary Table 1.

Supplementary Table 1. Linear Mixed Models

Information Criteria	US: Vaccination Simple Model	US: Vaccination Full Model	UK: Vaccination Simple Model	UK: Vaccination Full Model
-2 Log Likelihood	5253.189	4906	4846.48	4382.353
Akaike's Information Criterion (AIC)	5305.189	4968	4892.48	4438.353
Hurvich and Tsai's Criterion (AICC)	5306.273	4970	4893.34	4439.724
Bozdogan's Criterion (CAIC)	5466.048	5158	5034.64	4609.2
Schwarz's Bayesian Criterion (BIC)	5440.048	5127	5011.64	4581.2

Information Criteria	US: Ventilator Simple Model	US: Ventilator Full Model	UK: Ventilator Simple Model	UK: Ventilator Full Model
-2 Log Likelihood	4793.874	4733.97	3845.598	3758.101
Akaike's Information Criterion (AIC)	4847.874	4797.97	3893.598	3816.101
Hurvich and Tsai's Criterion (AICC)	4849.092	4799.68	3894.575	3817.529
Bozdogan's Criterion (CAIC)	5013.837	4994.616	4040.817	3993.85
Schwarz's Bayesian Criterion (BIC)	4986.837	4962.616	4016.817	3964.85

**American Sample: Vaccination Allocation**

For peer review only - http://bmjopen.bmj.com/site/about/guidelines.xhtml

	Numerator df	Denominator df	F	Sig.
Intercept	1	661	0.129	0.72
Scenario Type (Group or Patient)	1	661	63.748	< .0001
Age	1	661	0.408	0.523
Race (US)	5	661	1.706	0.131
Gender	3	661	7.238	< .0001
Highest Schooling	8	661	1.157	0.323
Social Status	1	661	0.089	0.765
Household Income	1	661	0.726	0.394
Political Orientation (General)	1	661	3.32	0.069
Political Orientation (Economical)	1	661	0.526	0.469
Political Orientation (Social)	1	661	11.382	0.001

UK Sample: Vaccination Allocation

	Numerator df	Denominator df	F	Sig.
Intercept	1	657	1.544	0.214
Scenario Type (Group or Patient)	1	657	91.289	< .0001
Age	1	657	0.233	0.63
Race (UK)	5	657	1.212	0.302
Gender	1	657	0.272	0.602
Highest Schooling	7	657	0.762	0.62
Social Status	1	657	1.985	0.159
Household Income	1	657	0.013	0.909
Political Orientation (General)	1	657	0	0.988
Political Orientation (Economical)	1	657	0.138	0.71
Political Orientation (Social)	1	657	0.244	0.621

American Sample: Vaccination – Full Model

For peer review only - http://bmjopen.bmj.com/site/about/guidelines.xhtml

	Numerator df	Denominator df	F	Sig.
Intercept	1	621	0.371	0.543
Scenario Type (Group or Patient)	1	621	61.082	< .0001
Age	1	621	1.171	0.28
Race (US)	5	621	1.328	0.25
Gender	3	621	4.702	0.003
Highest Schooling	8	621	0.678	0.711
Social Status	1	621	0.16	0.689
Household Income	1	621	0.096	0.756
Political Orientation (General)	1	621	5.125	0.024
Political Orientation (Economical)	1	621	0.008	0.927
Political Orientation (Social)	1	621	6.729	0.01
Injustice Perception	1	621	0.641	0.424
Responsibility Perception	1	621	2.589	0.108
Warmth Perception	1	621	0.012	0.912
Competence Perception	1	621	1.488	0.223
Modern Racism	1	621	23.16	< .0001

UK Sample: Vaccination – Full Model

	Numerator df	Denominator df	F	Sig.
Intercept	1	607	0.809	0.369
Scenario Type (Group or Patient)	1	607	89.533	< .0001
Age	1	607	3.054	0.081
Race (UK)	1	607	0.023	0.879
Gender	7	607	1.176	0.315
Highest Schooling	1	607	2.438	0.119
Social Status	1	607	0.091	0.763
Household Income	1	607	0.304	0.582
Political Orientation (General)	1	607	0.171	0.68
Political Orientation (Economical)	1	607	1	0.318

For peer review only - http://bmjopen.bmj.com/site/about/guidelines.xhtml

Political Orientation (Social)	5	607	0.96	0.442
Injustice Perception	1	607	0.027	0.87
Responsibility Perception	1	607	14.238	< .0001
Warmth Perception	1	607	0.048	0.826
Competence Perception	1	607	7.638	0.006
Modern Racism	1	607	16.986	< .0001

American Sample: Ventilator

	**Numerator df**	**Denominator df**	**F**	**Sig.**
Intercept	1	635	4.907	0.027
Scenario Type (Group or Patient)	1	635	2.418	0.12
Age	1	635	1.195	0.275
Race (US)	6	635	1.386	0.218
Gender	3	635	3.6	0.013
Highest Schooling	8	635	1.795	0.075
Social Status	1	635	4.437	0.036
Household Income	1	635	12.582	0
Political Orientation (General)	1	635	0.043	0.836
Political Orientation (Economical)	1	635	0.011	0.917
Political Orientation (Social)	1	635	0.976	0.324

UK Sample: Ventilator

	Numerator df	Denominator df	F	Sig.
Intercept	1	627	12.266	0
Scenario Type (Group or Patient)	1	627	1.713	0.191
Age	1	627	5.755	0.017
Race (UK)	4	627	7.181	0
Gender	3	627	0.342	0.795
Highest Schooling	7	627	0.521	0.819

For peer review only - http://bmjopen.bmj.com/site/about/guidelines.xhtml

Social Status	1	627	0.514	0.474
Household Income	1	627	0.816	0.367
Political Orientation (General)	1	627	0.793	0.373
Political Orientation (Economical)	1	627	0.002	0.962
Political Orientation (Social)	1	627	0.26	0.611

US Sample: Ventilator

	Numerator df	Denominator df	F	Sig.
Intercept	1	634	2.806	0.094
Scenario Type (Group or Patient)	1	634	2.418	0.12
Age	1	634	0.757	0.385
Race (UK)	6	634	0.897	0.497
Gender	3	634	3.411	0.017
Highest Schooling	8	634	1.624	0.115
Social Status	1	634	3.699	0.055
Household Income	1	634	11.868	0.001
Political Orientation (General)	1	634	0.039	0.843
Political Orientation (Economical)	1	634	0.785	0.376
Political Orientation (Social)	1	634	0.047	0.829
Injustice Perception	1	634	0.778	0.378
Responsibility Perception	1	634	0.382	0.537
Warmth Perception	1	634	1.014	0.314
Competence Perception	1	634	9.688	0.002
Modern Racism	1	634	10.737	0.001

UK Sample: Ventilator

	Numerator df	Denominator df	F	Sig.
Intercept	1	624	1.419	0.234
Scenario Type (Group or Patient)	1	624	1.926	0.166

For peer review only

Age	1	624	1.316	0.252
Race (UK)	3	624	0.864	0.46
Gender	7	624	1.241	0.278
Highest Schooling	1	624	0.066	0.797
Social Status	1	624	0.913	0.34
Household Income	1	624	0.096	0.757
Political Orientation (General)	1	624	0.038	0.846
Political Orientation (Economical)	1	624	0.59	0.443
Political Orientation (Social)	4	624	4.889	0.001
Injustice Perception	1	624	2.732	0.099
Responsibility Perception	1	624	0.744	0.389
Warmth Perception	1	624	3.129	0.077
Competence Perception	1	624	4.65	0.031
Modern Racism	1	624	13.881	< .0001

3. Perceptions of Injustice, Responsibility, and Stereotypes

In the US and the UK, participants were more likely to believe that adverse COVID-19 related health outcomes were the result of injustice and outside the individual's control for race than for obesity/male sex [Supplementary Figure 2]. In particular, injustice was perceived differently for the three additional risk factors (F(2, 2577)=217.388, P <0.0001, partial eta^2 =0.144). Bonferroni-corrected posthoc comparisons indicated that participants most strongly attributed the increased numbers of deaths of racial minorities due to injustice, stronger than the deaths of men (P < .0001, 95% CI: 0.372-0.469), and obese people (P < 0.000, 95% CI: 0.300-0.399). For the latter two, participants indicate more injustice to obese people death's than to men (P < .0001, 95% CI: 0.035-0.108). Similarly, there were differences in perceived personal control for adverse outcomes from COVID (F(2, 2577)=284.123, P <.0001, partial eta^2 = .181). Racial minorities were perceived to have the least control over the higher death rate compared to men (P =0.009, CI 95: 0.011-0.103) and obese people (P < .0001, 95% CI:0.529-0.655). Obese people were perceived to have most control over the higher death rates attributable to COVID, higher control than men (P < .0001, 95% CI: 0.595-0.476) and people from racial minorities (P =0.009, 95% CI: 0.011-0.103).

For peer review only

For peer review only - http://bmjopen.bmj.com/site/about/guidelines.xhtml

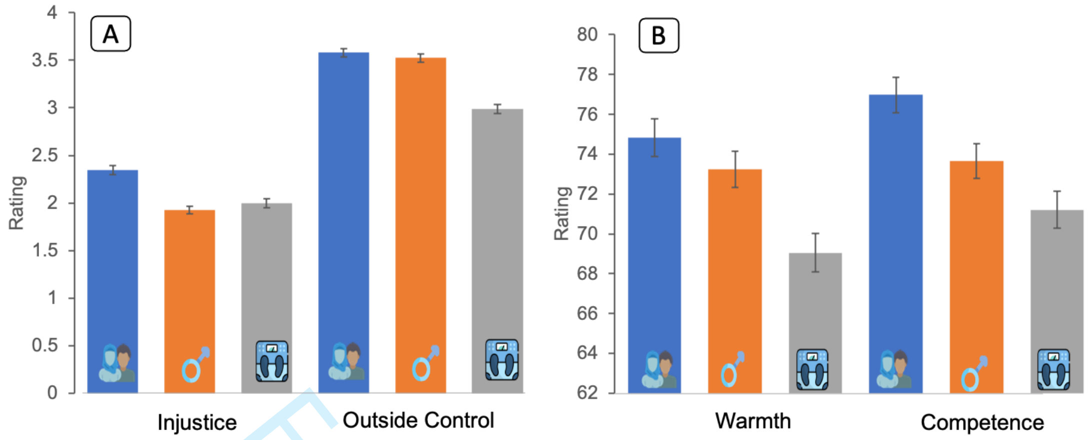

Supplementary Figure 2: Perceptions of Injustice, Control, Warmth, and Competence. A: Bonferroni-corrected comparisons indicated that participants most strongly attributed the increased numbers of deaths of racial minorities to injustice, stronger than the deaths of males (P < .0001, 95% CI: 0.372-0.469), and obese people (P < 0.000, 95% CI: 0.300-0.399). Participants attributed slightly more injustice to obese people's deaths than to males' deaths (P < .0001, 95% CI: 0.035-0.108). Similarly, racial minorities were perceived to have less control over the higher death rate compared to males (P =0.009, CI 95: 0.011-0.103) and obese people (P < .0001, 95% CI:0.529-0.655). Obese people were perceived to have the most control over higher death rates attributable to COVID-19, more than males (P < .0001, 95% CI: 0.595-0.476) and racial minorities (P =0.009, 95% CI: 0.011-0.103). B: Survey participants had the strongest stereotype content feelings of warmth towards ethical minorities, compared to attitudes towards males (p =0.005, Bonferroni corrected, small difference) and obese people (P < 0.0001, Bonferroni corrected). Participants also expressed higher feelings of competence toward individuals from a racial minority than males (P < 0.0001, Bonferroni corrected), or obese people (P < 0.0001, Bonferroni corrected). (Error bars = 95% Confidence Interval).

How did these perceptions of injustice and responsibility differ between the US and the UK? For injustice, we found no main effect of country (F(1,2576) = 2.534, P =.112, partial eta^2 =.001) and no interaction effect with categories of disadvantage (F(1,2576) = 1.622, P =0.203, partial eta^2 =.001). Participants in both countries perceived the relative injustice that affected each group similarly. When examining responsibility, we found no main effect of country (F(1,2576) = 0.366, P =0.545, partial eta^2 = 0.00001), but an interaction between country and category (F(1,2576) =31.651, P < .0001, partial eta^2 = 0.012). Participants in the US gave men less responsibility (t(2578) = 4.091, P < 0.0001), but obese people more responsibility (t(2578) = 4.176 .000, P < 0.0001) than participants in the UK.

Perceptions of injustice and responsibility might be related to the general attitudes towards a particular category. Attitudes can be characterized via two different dimensions, warmth and competence[56,57]**.** We found that again, respondents had different feelings of warmth towards different categories (F(1, 2577) = 72.446, P <0.0001, partial eta^2 = .029) and competence (F(1, 2577) = 76.369, P < 0.0001, partial eta^2 =0.030). In particular, survey participants had strongest feelings of warmth towards ethical minorities, compared to feelings towards men (p =0.005, Bonferroni corrected, small difference) and obese people (P < 0.0001, Bonferroni corrected), and rated the warmth of obese people lower than the warmth of men (P < 0.0001, Bonferroni corrected). When examining competence, we found a similar effect of group membership (F(2,2575) = 77.00, P < 0.0001, partial eta^2 = .03). Participants expressed higher feelings of competence in individuals from a racial minority than men (P < 0.0001, Bonferroni corrected), or obese people (P < 0.0001, Bonferroni corrected), while men were perceived as more competent than obese people (P < 0.0001, Bonferroni corrected).

For peer review only - http://bmjopen.bmj.com/site/about/guidelines.xhtml

Again, one might wonder whether participants from the US and the UK had different stereotypes about racial minorities, men, and obese people. When examining the effect of country on warmth, we find a main effect of country, F(1,2497) = 19.408, P < 0.0001, partial eta^2 = .008, American participants gave higher warmth ratings overall, though this varied between categories, F(2,2497) = 15.083, P < .0001, partial eta^2 = 0.006. The biggest difference was in warmth perception of obese participants, with American participants rating obese people more warmly than British participants (t(2495) = 5.73, P < .001), and only very small differences for the other two categories between both countries. When examining the effect of country and competence ratings, a similar picture emerged. We found a main effect for country (F(2,2497) = 25.16, P <.0001 , partial eta^2 = .01, which was qualified by an interaction effect with disadvantage categories, F(2,2497) = 17.49, P <001 , partial eta^2 = .007). Again, the main difference was for perceptions of obese people, Americans perceived obese people as more competent than British people, t(2497) = 6.672, P < .0001).

Why do people perceive the effect of a specific additional risk factor such as race as unjust? Part of the reason might be because of how favorably one thinks about the additional risk factor in general. To test this idea, we regressed feelings of responsibility, warmth, and competence on perceptions of injustice for each of the three additional risk factors. For race, we found that perceptions of responsibility (beta = .130, t(2482) = 6.551, P <.0001) and feelings of warmth, (beta = .209, t(2482) = 6.89, P < .0001) predicted the perception of injustice, but not perceptions of competence (beta = -.039, t(2482) = 1.309, P =0.191). For men, neither responsibility (beta = .032, t(2482) = 1.589,P = 0.112), competence (beta-.054, t-1.930 P = 0.054), or warmth (beta = .035, t1.262, P = 0.207) predicted perceptions of injustice. In contrast, for obesity, responsibility (beta = .222, t(2482) = 11.047, P < 0.0001), warmth (beta = .103, t(2482) = 3.587, P < 0.0001), and competence (beta = -.074, t(2482) =2.624, P = 0.009) predicted perceptions of injustice.

4. Allocation attitudes for participants with low and high modern racism scores

In the US and the UK, responses on the Modern Racism Scale were associated with attitudes towards ventilator and vaccine allocation based on race (Ventilator: US: F(1,634) = 10.73, p = 0.001, UK: F(1,624) = 13.88, P < .0001; Vaccine: US: F(1,621 =23.00, P <0.0001, UK: F(1,607)=16.98, P <0.0001). The more participants endorsed modern racism statements, the less positive their composite attitudes towards ventilator or vaccine allocation to racial minority patients. A minority (27.3%) of participants had a mean score of higher than three on the modern racism scale, indicating an endorsement of modern racism. These participants did not give weight to race in ventilator or vaccine allocations [Supplementary Figure 3]. Participants with low modern racism scores also did not give weight to race in ventilator allocation but did for vaccines. Modern racism did not affect prioritisation based on sex/obesity [Supplementary Figure 3]. Perceptions of injustice did not predict ventilator or vaccine allocation attitudes in the US (F(1,634) = 0.778, P = 0.378) or the UK (F(1,624) = 2.732, P = 0.099).

For peer review only - http://bmjopen.bmj.com/site/about/guidelines.xhtml

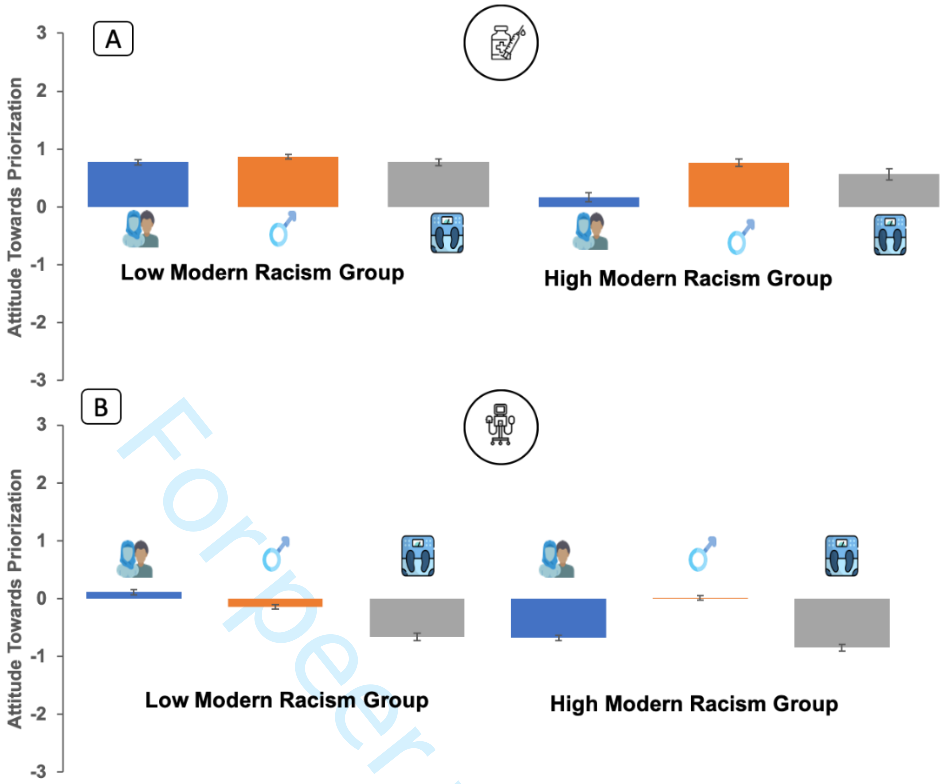

Supplementary Figure 3: Allocation attitudes for participants with low modern racism scores (Low Modern Racism Group) and high modern racism scores (High Modern Racism Group). A: For vaccines, participants in the Low Modern Racism Group (mean score lower than scale midpoint, N = 1011) had a more positive attitude towards race-based vaccine allocation compared to participants in the High Modern Racism Group (mean score higher than scale midpoint, N = 307). Allocation attitudes for sex and obesity were similar. B: For ventilators, we find that participants in the Low Modern Racism Group (N = 919) had a neutral attitude towards race-based vaccine allocation, while participants in the High Modern Racism Group (N = 343) had a negative one. Allocation attitudes for sex and obesity were similar.

References (Supplemental Materials)

1. J. B. McConahay, “Modern racism, ambivalence, and the Modern Racism Scale” in *Prejudice, Discrimination, and Racism*, J. F. Dovidio, S. L. Gaertner, Eds. (Academic Press, 1986), pp. 91–125.

2. T. G. Morrison, M. Kiss, Modern racism scale. *Encyclopedia of personality and individual differences*, 1–3 (2017).

3. , U.S. Census Bureau QuickFacts: United States (July 22, 2021).

4. D. Wilkinson, H. Zohny, A. Kappes, W. Sinnott-Armstrong, J. Savulescu, Which factors should be included in triage? An online survey of the attitudes of the UK general public to pandemic triage dilemmas. *BMJ Open* **10**, e045593 (2020).

For peer review only - http://bmjopen.bmj.com/site/about/guidelines.xhtml

5. , Why are more people from BAME backgrounds dying from coronavirus? *BBC News* (2020) (June 28, 2021).

6. T. Takahashi, *et al.*, Sex differences in immune responses that underlie COVID-19 disease outcomes. *Nature* **588**, 315–320 (2020).

7. J. Yang, J. Hu, C. Zhu, Obesity aggravates COVID-19: a systematic review and meta-analysis. *Journal of medical virology* **93**, 257–261 (2021).

8. P. Slovic, "If i look at the mass i will never act: Psychic numbingpsychic numbing and genocidegenocide" in *Emotions and Risky Technologies*, (Springer, 2010), pp. 37–59.

9. S. T. Fiske, A. J. Cuddy, P. Glick, J. Xu, "A model of (often mixed) stereotype content: Competence and warmth respectively follow from perceived status and competition (2002)" in *Social Cognition*, (Routledge, 2018), pp. 171–222.

10. S. T. Fiske, A. J. Cuddy, P. Glick, Universal dimensions of social cognition: Warmth and competence. *Trends in cognitive sciences* **11**, 77–83 (2007).

11. C. W. Blatz, M. Ross, Principled ideology or racism: Why do modern racists oppose race-based social justice programs? *Journal of Experimental Social Psychology* **45**, 258–261 (2009).

For peer review only

STROBE Statement—Checklist of items that should be included in reports of ***cross-sectional studies***

	Item No	Recommendation	Page number
**Title and abstract**	1	(*a*) Indicate the study's design with a commonly used term in the title or the abstract	1
		(*b*) Provide in the abstract an informative and balanced summary of what was done and what was found	2
**Introduction**			
Background/rationale	2	Explain the scientific background and rationale for the investigation being reported	3
Objectives	3	State specific objectives, including any prespecified hypotheses	3
**Methods**			
Study design	4	Present key elements of study design early in the paper	4
Setting	5	Describe the setting, locations, and relevant dates, including periods of recruitment, exposure, follow-up, and data collection	4
Participants	6	(*a*) Give the eligibility criteria, and the sources and methods of selection of participants	5
Variables	7	Clearly define all outcomes, exposures, predictors, potential confounders, and effect modifiers. Give diagnostic criteria, if applicable	5
Data sources/ measurement	8*	For each variable of interest, give sources of data and details of methods of assessment (measurement). Describe comparability of assessment methods if there is more than one group	5
Bias	9	Describe any efforts to address potential sources of bias	4
Study size	10	Explain how the study size was arrived at	4
Quantitative variables	11	Explain how quantitative variables were handled in the analyses. If applicable, describe which groupings were chosen and why	5 to 6
Statistical methods	12	(*a*) Describe all statistical methods, including those used to control for confounding	6
		(*b*) Describe any methods used to examine subgroups and interactions	BAME: 7, Racism: 7 (Supplementary Figure 3)
		(*c*) Explain how missing data were addressed	NA
		(*d*) If applicable, describe analytical methods taking account of sampling strategy	NA
		(*e*) Describe any sensitivity analyses	NA
**Results**			
Participants	13*	(a) Report numbers of individuals at each stage of study—eg numbers potentially eligible, examined for eligibility, confirmed eligible, included in the study, completing follow-up, and analysed	5
		(b) Give reasons for non-participation at each stage	NA
		(c) Consider use of a flow diagram	NA

For peer review only - http://bmjopen.bmj.com/site/about/guidelines.xhtml

Descriptive data	14*	(a) Give characteristics of study participants (eg demographic, clinical, social) and information on exposures and potential confounders	4, Table 1
		(b) Indicate number of participants with missing data for each variable of interest	NA
Outcome data	15*	Report numbers of outcome events or summary measures	6 to 7
Main results	16	(*a*) Give unadjusted estimates and, if applicable, confounder-adjusted estimates and their precision (eg, 95% confidence interval). Make clear which confounders were adjusted for and why they were included	Unadjusted estimates with CIS: 7 to 8
		(*b*) Report category boundaries when continuous variables were categorized	Modern Racism Scale: 8
		(*c*) If relevant, consider translating estimates of relative risk into absolute risk for a meaningful time period	NA
Other analyses	17	Report other analyses done—eg analyses of subgroups and interactions, and sensitivity analyses	6 to 8
**Discussion**			
Key results	18	Summarise key results with reference to study objectives	8
Limitations	19	Discuss limitations of the study, taking into account sources of potential bias or imprecision. Discuss both direction and magnitude of any potential bias	10
Interpretation	20	Give a cautious overall interpretation of results considering objectives, limitations, multiplicity of analyses, results from similar studies, and other relevant evidence	9-11
Generalisability	21	Discuss the generalisability (external validity) of the study results	9 to 11
**Other information**			
Funding	22	Give the source of funding and the role of the funders for the present study and, if applicable, for the original study on which the present article is based	11

*Give information separately for exposed and unexposed groups.

Note: An Explanation and Elaboration article discusses each checklist item and gives methodological background and published examples of transparent reporting. The STROBE checklist is best used in conjunction with this article (freely available on the Web sites of PLoS Medicine at http://www.plosmedicine.org/, Annals of Internal Medicine at http://www.annals.org/, and Epidemiology at http://www.epidem.com/). Information on the STROBE Initiative is available at www.strobe-statement.org.

For peer review only - http://bmjopen.bmj.com/site/about/guidelines.xhtml

BMJ Open

Race and resource allocation: An online survey of US and UK adults' attitudes towards COVID-19 ventilator and vaccine distribution

Journal:	*BMJ Open*
Manuscript ID	bmjopen-2022-062561.R2
Article Type:	Original research
Date Submitted by the Author:	09-Sep-2022
Complete List of Authors:	Kappes, Andreas; City University of London<br>Zohny, Hazem; University of Oxford,<br>Savulescu, Julian; University of Oxford, MEDICAL ASSESSMENT UNIT<br>Singh, Ilina; University of Oxford, Psychiatry<br>Sinnott-Armstrong, Walter; Duke University<br>Wilkinson, Dominic; University of Oxford, Oxford Uehiro Centre for Practical Ethics; John Radcliffe Hospital,
**Primary Subject Heading**:	Ethics
Secondary Subject Heading:	Public health
Keywords:	Health policy < HEALTH SERVICES ADMINISTRATION & MANAGEMENT, MEDICAL ETHICS, COVID-19

SCHOLARONE™
Manuscripts

I, the Submitting Author has the right to grant and does grant on behalf of all authors of the Work (as defined in the below author licence), an exclusive licence and/or a non-exclusive licence for contributions from authors who are: i) UK Crown employees; ii) where BMJ has agreed a CC-BY licence shall apply, and/or iii) in accordance with the terms applicable for US Federal Government officers or employees acting as part of their official duties; on a worldwide, perpetual, irrevocable, royalty-free basis to BMJ Publishing Group Ltd ("BMJ") its licensees and where the relevant Journal is co-owned by BMJ to the co-owners of the Journal, to publish the Work in this journal and any other BMJ products and to exploit all rights, as set out in our licence.

The Submitting Author accepts and understands that any supply made under these terms is made by BMJ to the Submitting Author unless you are acting as an employee on behalf of your employer or a postgraduate student of an affiliated institution which is paying any applicable article publishing charge ("APC") for Open Access articles. Where the Submitting Author wishes to make the Work available on an Open Access basis (and intends to pay the relevant APC), the terms of reuse of such Open Access shall be governed by a Creative Commons licence – details of these licences and which Creative Commons licence will apply to this Work are set out in our licence referred to above.

Other than as permitted in any relevant BMJ Author's Self Archiving Policies, I confirm this Work has not been accepted for publication elsewhere, is not being considered for publication elsewhere and does not duplicate material already published. I confirm all authors consent to publication of this Work and authorise the granting of this licence.

For peer review only

Race and resource allocation: An online survey of US and UK adults' attitudes towards COVID-19 ventilator and vaccine distribution

Andreas Kappes PhD[1], Hazem Zohny PhD[2], Julian Savulescu PhD[2,3], Ilina Singh PhD[4,5], Walter Sinnott-Armstrong PhD[6], Dominic Wilkinson DPhil[2,3,7]

Affiliations:
[1]City, University of London
[2] Oxford Uehiro Centre for Practical Ethics, University of Oxford
[3] Murdoch Children's Research Institute, Melbourne, Australia.
[4] Wellcome Trust Centre for Ethics and the Humanities, University of Oxford
[5] Department of Psychiatry, University of Oxford
[6] Kenan Institute for Ethics and Philosophy Department, Duke University
[7] John Radcliffe Hospital, Oxford, UK

Correspondence: Dr Hazem Zohny, Oxford Uehiro Centre for Practical Ethics, Suite 8, Littlegate House, St Ebbes St, Oxford, OX1 1PT, UK. Tel: +44 1865 286888, Email: hazem.zohny@philosophy.ox.ac.uk

For peer review only

Abstract

Objective: This study aimed to assess US/UK adults' attitudes towards COVID-19 ventilator and vaccine allocation.

Design: Online survey including US and UK adults, sampled to be representative for sex, age, race, household income, and employment. A total of 2580 participated [female = 1289, Age Range = 18 to 85, Black American = 114, BAME = 138].

Interventions: Participants were asked to allocate ventilators or vaccines in scenarios involving individuals or groups with different medical risk and additional risk factors.

Results: Participant race did not impact vaccine or ventilator allocation decisions in the US, but did impact ventilator allocation attitudes in the UK ($F(4,602) = 6.95$, $P < 0.001$). When a racial minority or White patient had identical chances of survival, 14.8% allocated a ventilator to the minority patient (UK BAME participants: 24.4%), and 68.9% chose to toss a coin. When the racial minority patient had a 10% lower chance of survival, 12.4% participants allocated them the ventilator (UK BAME participants: 22.1%). For patients with identical risk of severe COVID-19, 43.6% allocated a vaccine to a minority patient, 7.2% chose a White patient, and 49.2% chose a coin toss. When the racial minority patient had a 10% lower risk of severe COVID-19, 23.7% participants allocated the vaccine to the minority patient. Similar results were seen for obesity or male sex as additional risk factors. In both countries, responses on the Modern Racism Scale were strongly associated with attitudes towards race-based ventilator and vaccine allocations ($P < 0.0001$).

Conclusions: Although living in countries with high racial inequality during a pandemic, most US and UK adults in our survey allocated ventilators and vaccines preferentially to those with the highest chance of survival or highest chance of severe illness. Race of recipient led to vaccine prioritisation in cases where risk of illness was similar.

Strengths and limitations of this study:

- Our scenarios enable comparison of approaches to prioritisation of ventilators and vaccines in the face of scarcity and high demand
- The survey provides insights into the relative weights given to equity and race compared to medical factors in two different countries.
- To control variables, hypothetical scenarios were not realistic and may not align with lived experience.
- Survey responses permit quantification of attitudes to prioritisation, but not the reasons behind answers.
- We sought large nationally representative samples, but Hispanic and Black participants were under-represented among US respondents, and members of non-academic minoritised groups were not included in the study design or analysis.

Introduction

For peer review only - http://bmjopen.bmj.com/site/about/guidelines.xhtml

The coronavirus pandemic has raised contentious ethical questions about the distribution of scarce healthcare resources during a crisis. For example, early concern about the supply of intensive care beds and mechanical ventilators led healthcare systems to develop triage criteria in the event of a shortage.[1,2] Later, states had to decide whom to prioritise for access to the limited supply of vaccines.[3,4] Although different countries have followed different strategies, a prevailing response to these questions has focused on saving the most lives.[3,5–8] For ventilator triage, this entailed giving priority to those with the highest chance of survival.[9] For vaccines, those with the highest risk of severe illness were prioritised.[10]

However, this has been criticised[11] for ignoring evidence that individuals from racial minorities have been disproportionately affected by the pandemic.[12–14] In the US, Black, Hispanic and Indigenous people were more likely to be infected, hospitalised and die of COVID-19 than White individuals.[15] Similarly, high morbidity and mortality rates were seen in ethnic minority groups in the UK.[16] This raises an important ethical question: should individuals from disproportionately affected racial minorities be prioritised when allocating scarce resources such as ventilators and vaccines? If so, how should this be weighed against other ethical values in resource allocation, including the desire to save the most lives and the need to treat patients equally?

Some legal scholars, policy advocates, and ethicists have suggested that race should be factored into ventilator and vaccine allocation.[17–19] For example, some have proposed positive discrimination in the form of equity weights.[12] However, such an approach might raise political[20] or legal concerns.[21] Alternatively, it could lead to ethical questions. Treating members of racial groups differently would conflict with principles of equality. Furthermore, in some circumstances, preferential allocation to members of a disadvantaged group may possibly increase overall mortality from COVID-19.[22,23] On the other hand, not considering it could widen the race-based difference in COVID-19 deaths and conflict with the principle of equity.

Policy discussion during the pandemic has largely lacked information on public opinion: it is not known what people in countries like the US and UK, with high racial inequality in COVID-19 deaths, think about these questions. There is some evidence that the public support including race in allocation of vaccines,[24] but none about the *relative* importance of medical versus racial factors.

We sought to examine the relative weight given by the public to race in allocation and to compare this with participants' views on obesity and male sex. Race, obesity, and sex are independent risk factors for COVID-19 (in one UK study, hazard ratios for COVID-19 death were 1.48, 1.40, and 1.59, respectively[25]), but they differ ethically (for example, in their association with historical injustice). Finally, we sought to explore whether prioritisation preferences differed between racial groups, socio-economic status, or political orientations or were related to stereotypes and covert racial attitudes. This would illuminate what drives prioritisation attitudes but also whether a broader consensus exists.

For peer review only - http://bmjopen.bmj.com/site/about/guidelines.xhtml

Methods

The study was approved by the University of Oxford Central University Research Ethics Committee [R73841/RE002]. It follows the American Association for Public Opinion Research (AAPOR) reporting guideline. All data, code, and materials used in the analysis is are available in a public, open access repository.[26]

Participants were recruited from large market research panels and paid $10.8/hour. Speed and attention checks were used to identify and exclude respondents not paying sufficient attention to question details.

Two surveys were conducted in December 2020/January 2021, (US n=1296, UK n=1284). (Table 1). Each sub-sample size gave the ability to estimate true preferences with a 4% margin of error (95% confidence interval) for the US and the UK population. The survey achieved a completion rate (completed surveys divided by number of respondents who entered the survey) of 86.7%. As the number of people that had the chance to participate in the opt-in panel is not known, we cannot report the AAPOR RR1[9].

Table 1: Demographic Details of Participants. Participants were sampled to be representatives of the US and UK general population for sex, age, race, household income, and employment. (Different income categories were used to reflect known information on population characteristics). Fourteen percent of the UK sample, and 19% of the US sample described their racial background as non-White. Note that, compared to the last census[27], the US sample had a lower proportion of Hispanic and Black participants than the general US population.

	UK / US	US Sample	UK Sample
		n (%)	n (%)
**Gender**	Male	634 (48.9%)	630 (49.1%)
	Female	656 (50.6%)	650 (50.6%
	Other	3 (0.2%)	3 (0.2%)
	Prefer not to say	3 (0.2%)	1 (0.1%)
**Race**	White	1034 (79.8%)	1103 (85.9%)
	Hispanic or Latino	70 (5.4%)	-
	Black or African American / Black or Black British	114 (8.8%)	41 (3.2%)
	Native American or American Indian	19 (1.5%)	-
	Mixed	-	29 (2.3%)
	Asian or Pacific Islander / Asian or Asian British	23 (1.8%)	98 (7.6%)
	Other	21 (1.6%)	11 (0.9%)
	Prefer not to say	11 (0.8%)	2 (0.2%)
**Age**	18 - 24	159 (15.5%)	145 (11.3%))
	25 - 34	248 (18.9%)	303 *23.6%)
	35 - 44	237 (18.9%)	291 (*22.7%)
	45 - 54	252 (18.3%)	238 (18.5%)
	55 - 64	224 (14.2%)	198 (15.4%)
	65 - 74	129 (5.9%)	89 (6.9%)
	75 - 84	38 (1.6%)	20 *1.6%)
	85 or older	4 (0.6%)	-
**Highest level of schooling**	Less than high school degree / Primary school	24 (1.9%)	3 (0.2%)
	High school graduate or equivalent / Secondary school up to 16 years	237 (18.3%)	236 (18.4%)
	Some college but no degree / Higher or secondary or further education	250 (19.3%)	441 (34.3%))
	Associate degree in college (2-year)	122 (9.4%)	-

For peer review only - http://bmjopen.bmj.com/site/about/guidelines.xhtml

	Bachelor's degree	160 (12.3%)	381 29.7%)
	Master's degree	290 (22.4%)	138 (10.7%)
	Doctoral degree	29 (2.2%)	29 (2.3%)
	Professional degree (JD, MD)	38 (2.9%)	47 (3.7%)
	Prefer not to say	6 (0.5%)	9 (0.7%)
	Other	140 (10.8%)	-
**Employment status**	Employed part time	136 (10.5%)	735 (57.2%)
	Employed full time	617 (47.6%)	232 (18.1%)
	Unemployed looking for work	96 (7.4%)	70 (5.5%)
	Unemployed not looking for work	113 (8.7%)	96 (7.5%)
	Retired	274 (21.1%)	114 (8.9%)
	Student	48 (3.7%)	37 (2.9%)
	Disabled	12 (0.9%)	-
**Estimated Household Income**	$0-$25k / £0-£20k	230 (17%)	263 (20.5%)
	$25k-$50k / £20k-£30k	274 (21.1%)	276 (21.5%)
	£30k-£40k	-	198 (15.4%)
	£40k-£50k	-	145 (11.3%)
	$50k-$75k / £50k-£60k	224 (17.3%)	106 (8.3%)
	$75k-$100k / £60k-£100k	168 (13%)	191 (14.9%)
	$100k-$150k / £100k+	199 (15.4%)	105 (8.2%)
	$150k+	103 (7.9%)	-
	Prefer not to say	11 (0.8%)	-

The surveys were adapted from a previous study.[28] Participants were asked to imagine that they needed to make decisions in the setting of a severe shortage of ventilators or vaccines. To examine how much weight participants would give to predicted chance (based on age and comorbidity) of survival for allocating ventilators, or of developing severe COVID-19 for vaccines, participants read scenarios where potential recipients differed in those variables to different degrees. For example, in one ventilator scenario, one patient had a 50% chance of survival, the other a 40% chance. In a corresponding vaccine scenario, participants had a 20% vs 10% chance of developing severe COVID-19. Participants decided whom to give the ventilator or vaccine to or to toss a coin (Figure 1). Thereafter, participants were randomly introduced to one of the three additional factors: race, sex, and obesity.

For a racial minority in the US, we chose 'Black recipients' and, in the UK, 'BAME recipients'. BAME is a common term in the United Kingdom, used to describe non-White ethnic groups.[29] However, while commonly used, the term is no longer in official use as it combines ethnic groups with distinct identities.[30] Before completing scenarios, we reminded participants of the racial inequality in COVID-19-related deaths in the UK and in the US. Thereafter, participants worked on five scenarios involving one Black (BAME) and one White patient. In one scenario, both patients had the same chance of surviving/severe COVID-19; in two scenarios the Black (BAME) patient had a higher chance of survival/severe COVID-19; in two scenarios the White patient had a higher chance. Participants indicated for each whether they wanted to give the ventilator/vaccine to the patient from a racial minority, the White patient, or toss a coin.

Participants saw a similar set of scenarios for sex and obesity as additional risk factors [SM]. To test whether it would make a difference, we repeated the scenarios for groups of patients.

We asked participants questions to capture their perceptions of racial minorities, men, and obese people, including their views about the degree to which worse outcomes from COVID-19 were a result of social injustice or under the control of the individual. We also

For peer review only - http://bmjopen.bmj.com/site/about/guidelines.xhtml

administered the Modern Racism Scale, intended to capture covert discriminatory attitudes.[31] The Modern Racism Scale is one of the most commonly used and best validated instruments to examine prejudice against black people in the US.[32]

Statistical analysis

To examine the differences between risk factors, and the variables that impacted participants' attitudes, we created a composite attitude score for each set of scenarios by counting the number of times participants decided to give the ventilator or vaccine to the person or group with an additional risk factor in successive scenarios. To test whether differences existed between risk factors, we used repeated measure analysis of variance with Bonferroni corrected post-hoc comparisons. To test which variables impacted participants' attitudes towards risk factors, we ran a series of four linear models to explore the role of various factors in predicting racial prioritisation attitudes. For each model, we defined attitudes as dependent variable and subjects as the random factor. In the first two models, we entered age, gender, race, relative income, educational level, political ideology (general, social, and economical), and type of scenario (patient versus group-based) as fixed factors. In the second two models, we additionally added perceptions of injustice, responsibility, warmth, competence, and modern racism scores. The full results of each model can be seen in the Supplemental Results (Supplementary Table 1). We used SPSS 28 for all analyses.

Patient and public involvement

No patient involvement.

Results

Prioritisation of medical risk

We first assessed the impact of medical risk factors on allocation. Asked to choose between patients 50% vs 40% chance of surviving if treated, 71.9% (95% CI: 69.3%-74.3%,) of participants gave the ventilator to the patient with higher chances. For vaccines (15% vs 10% chance of severe COVID-19), 70.8% (95% CI: 68.3%-73.2%) gave the vaccine to the higher risk recipient. This increased when the difference between patients increased (Figure 2). When there was no difference, 82.7 (86%) (95% CI: 80.5-84.8,84.6-88.4) tossed a coin. Patterns of response were very similar for scenarios involving groups as for individual patients (Figure 2).

Risk factors and resource allocation

In scenarios involving patients with identical chances of survival but different race, 14.8 of participants allocated the ventilator to Black or BAME patients, 68.9% chose to toss a coin, and 16.2% chose the White patient. For vaccine allocation to patients with identical chances of severe illness, 43.6% of participants allocated the vaccine to Black or BAME patients, 49.2% chose to toss a coin, and 7.2% chose the White patient. When the racial minority patient had a lower chance of survival or severe illness, <15% of respondents allocated the ventilator or vaccine to them (Figure 3).

For peer review only - http://bmjopen.bmj.com/site/about/guidelines.xhtml

When chances of survival were equal, but one patient was male and the other female, 11.9% (95% CI: 10.2%,13.8%) of participants gave the ventilator to the male, (22.7% female patient; 65.4% tossed a coin). When obesity was an additional risk factor, 11.9% (95% CI: 11.6% - 15.5%) chose the obese patient, (41.1% healthy weight, 45.4% tossed a coin).
For vaccines, in scenarios of equal medical risk, where one patient was male and the other female, 44.2% of participants gave the vaccine to the male patient. When obesity was an additional risk factor, 59.2% allocated to an obese patient (Figure 3).

Using the composite attitude score, race and sex did not affect allocation decisions for ventilators, but obesity did so negatively: ($P < .001$, 95% CI -.9485, -.7124). Participants had lower prioritisation preferences for obese patients, (compared with racial minority, $P < 0.001$, and male patients, $P = .030$). For vaccines, participants had significant prioritisation preference for race, sex, and obesity (attitude greater than zero: $p < .00001$; post-hoc analyses: obese patients > racial minorities, $P < 0.001$, and male patients, $P < 0.001$.) There was a very small main effect for scenario type (group preference – Supplementary Figure 1). Country of participants (US vs UK) did not alter allocation attitudes ($F(1,1260) = 0.619$, $P = 0.431$).

Determinants of prioritisation

Using mixed linear models [Supplementary Table 1, full results], we found that race of participant impacted ventilator allocation attitudes in the UK ($F(4,602) = 6.95$, $P < 0.001$) but not the US ($F(6, 635) = 1.38$, $P = 0.21$). A sensitivity analysis showed that the US sample afforded us a power of .99 to find a small difference between White and Black participants. In the UK, Asian and Black participants had more positive attitudes towards race in allocation of ventilators than White participants [SM]. Among BAME participants, 22.1 % of participants gave the ventilator to a BAME patient with a 10% lower chance of survival, while only 6.9% of the White participants did (see Table 2).

Table 2: Differences in responses between UK White and BAME participants for all scenarios involving ventilators and race

	Ethnic Minority Participants			White Participants		
	BAME Patient	Coin Toss	White Patient	BAME Patient	Coin Toss	White Patient
Equal Survival Chances	24.4	72.1	3.5	13	73.2	13.8
10% Higher Chance	77.9	11.6	10.5	73.2	15.3	11.5
30% Higher Chance	87.2	10.5	2.3	90.5	2	7.4
10% Lower Chance	22.1	15.1	62.8	6.9	15.5	77.7
30% Lower Chance	4.7	22.1	73.3	1.5	8	90.5

Age, gender, socio-economic status, education, and political ideology did not impact ventilator allocation attitudes. Those participants that strongly endorsed ventilator allocations based on chances of survival in the scenarios without any additional risk factors were not more or less likely to reject ventilator allocation based on race ($r = -0.047$).

Race of participants did not affect vaccine allocation. In the US, (but not the UK) gender ($F3,661) = 7.238$, $P < .0001$) and social political orientation $F(1,661) = 11.388$, $P = 0.001$)

For peer review only - http://bmjopen.bmj.com/site/about/guidelines.xhtml

For peer review only

impacted vaccine allocation based on race. Male participants had a less positive attitude (P = 0.003) and more socially conservative participants gave less weight to race in vaccine allocation (parameter estimate = - 0.305, 95% CI = -.480, -.130).

Influence of attitudes towards race on prioritisation

In both countries, participants were more likely to believe that adverse COVID-19 related health outcomes were the result of injustice and outside the individual's control for race than for obesity/male sex [Supplementary Figure 2]. Obesity was associated with the strongest sense of individuals' health outcomes being within their control. US and UK respondents had similar attitudes towards the respective racial minorities [SM].

In the US and the UK, responses on the Modern Racism Scale were associated with attitudes towards ventilator and vaccine allocation based on race (Ventilator: US: $F(1,634) = 10.73$, $p = 0.001$, UK: $F(1,624) = 13.88$, $P < .0001$; Vaccine: US: F(1,621 =23.00, $P <0.0001$, UK: $F(1,607)=16.98$, $P <0.0001$). The more participants endorsed modern racism statements, the less positive their composite attitudes towards ventilator or vaccine allocation to racial minority patients. A minority (27.3%) of participants had a mean score of higher than three on the Modern Racism Scale, indicating an endorsement of modern racism. These participants did not give weight to race in ventilator or vaccine allocations [Supplementary Figure 3]. Participants with low modern racism scores also did not give weight to race in ventilator allocation but did for vaccines. Modern racism did not affect prioritisation based on sex/obesity [Supplementary Figure 3]. Perceptions of injustice did not predict ventilator or vaccine allocation attitudes in the US ($F(1,634) = 0.778$, $P = 0.378$) or the UK ($F(1,624) = 2.732$, $P = 0.099$).

Discussion

Previous research has identified some of the public's prioritisation preferences in relation to race and vaccines.[24] To our knowledge, this is the first study to examine the views of the public on race in ventilator allocation and in comparison with other factors. This international study was conducted during the second wave of the COVID-19 pandemic in two countries with documented and publicised adverse outcomes for racial minorities. Survey participants largely allocated ventilators and vaccines on the basis of medical factors and independent of the race of recipients. They were highly sensitive to small differences in predicted outcome. For example, for a 10% difference in predicted survival, 71.9% of participants gave the ventilator to the patient with higher chances while 82.7% chose to toss a coin toss where there was no difference in predicted chance. For vaccine allocation we found similar results for even smaller differences in risk of severe Covid (5% difference). (It is important to note that such small differences in prognosis are clinically impossible to achieve but demonstrate participants' sensitivity to medical factors.)

Relatively few respondents in our survey, even those from racial minorities themselves, endorsed priority for ventilators for those from racial minorities. However, BAME respondents in the UK (but not Black respondents in the US) were more inclined than White respondents to preferentially allocate a ventilator to a patient from a racial minority. For vaccines, where other factors were equal, 40-60% of respondents allocated preferentially to patients/groups with additional risk based on race, sex, or obesity. In the survey, willingness

For peer review only - http://bmjopen.bmj.com/site/about/guidelines.xhtml

to prioritise based on additional risk did not correlate with participants' perceptions of whether risk factors were caused by social injustice. However, covert racial attitudes (endorsed by approximately a quarter of respondents) negatively impacted attitudes to allocation in both countries.

The different weight given to race in the allocation of vaccines versus ventilators in our study may reflect people's desire to prevent the most deaths.[7,33] Previous research has shown strong public endorsement for prioritising chances of survival in ventilator allocation and probability of severe illness for vaccine allocation.[24,28,34–36] However, whereas vaccines are provided to prevent illness, ventilators are provided to those already critically ill. Participants might have felt that allocating ventilators preferentially to those with additional risk factors (race, sex, obesity) could lead to more deaths from COVID-19 if such patients (even with otherwise equal medical risk) have higher mortality rates. Another possible explanation, at least for US participants, is that some were aware of more recent evidence of similar hospitalised critical care outcomes for Black patients.[37] This may have led some to conclude that since hospital admissions were in proportion to the occurrence of severe disease in the community, allocation of critical care resources preferentially would not be justified.[38] (Since the survey was conducted prior to much of this evidence emerging, this was potentially less likely to influence responses). Respondents might have regarded preferential allocation of a life-saving intervention like ventilators as more legally or ethically questionable than prioritisation of vaccines. Finally, participants might have reasoned that ventilators do not have wider community benefits, but vaccines do. Hence, preferential allocation of vaccines represents a more substantial (and possibly less controversial) means to reduce inequality.

However, in our study participants who expressed a strong attitude for ventilator allocation based on chances of survival were not more or less likely to reject preference in ventilator allocation based on race. Other ethical principles or considerations may be guiding allocation decisions, for example concern for equality and avoiding discrimination.[39,40] Respondents did not believe that racial minorities should be at a disadvantage for access to ventilators – reinforcing the importance of ensuring that medical criteria used for allocation are not racially biased.[41,42]

One reason to potentially give priority in allocation of scarce treatment to racial minorities is because of concerns over past or present injustice.[12] In our survey, UK and US respondents perceived higher death rates among minorities to be more unjust and less under individual control than the mortality rates of male and obese recipients. However, these perceptions did not affect allocation decisions. Similarly, political orientation had little impact on allocation decisions in our survey. More conservative respondents were less inclined to indicate that injustice is responsible for higher death rates. However, this perception did not affect allocation decisions.

Allocation decisions in our survey were associated with responses on the Modern Racism Scale. This instrument is intended to capture covert racial attitudes.[31] While people who score high on the Modern Racism Scale potentially do not perceive themselves as racist, discriminatory attitudes can nevertheless guide their decisions. Modern racism might measure negative attitude towards affirmative action policies in general. However, in our survey, modern racism did not predict attitudes to sex or obesity in allocation. Furthermore, in our models, political ideology was not associated with allocation based on race. Taken together, these findings suggest that some opposition to giving weight to race in prioritisation arises from covert racial attitudes. However, even those respondents with low scores on the Modern

For peer review only - http://bmjopen.bmj.com/site/about/guidelines.xhtml

Racism Scale (three quarters of those surveyed) did not on average give weight to race in allocation of ventilators. Covert racial attitudes do not appear to explain our overall finding of the lack of weight given to race in public attitudes to ventilator allocation.

Importantly our results help contextualise previous findings that looked at race in allocation in isolation. For example, a US Gallup/COVID Collaborative survey found that 74-85% respondents supported giving priority for vaccine access to Black, Hispanic and Native Americans.[24] In our study, slightly less than half of respondents endorsed this, but only in cases where other risk factors were equal. This highlights the importance of clarifying the weight given to different ethical values when surveying the public's views on resource allocation, but also points to the importance of alternative approaches to lessening the impact of Covid-19 on racial minorities. One promising future direction would be to use computational models of decision-making to quantify the weight people give in their allocation decision to different medical and social factors when presented simultaneously, as is the case in clinical practice.

Strengths and limitations

The hypothetical scenarios used in our study enabled us to assess and compare the attitude of the public towards allocation of two key resources in a pandemic. The use of parallel examples enabled appraisal of the relative ethical weight given to some important risk factors compared with the risk of serious illness or death. Scenarios were highly specific, somewhat artificial, and clinically unrealistic (particularly in relation to small predicted differences between patients or groups). This could have differed from participants' lived experience and may have contributed to unease in prioritisation decisions. The survey design did not follow an explicit social justice framework.[43,44]

Survey responses allowed us to quantify some elements of the public's values, but we were not able to assess the reasons why particular answers were given. Future research should explore this.

The survey was conducted during the COVID-19 pandemic at a fraught and emotional time. This would have increased the salience of the survey for participants, and could provide a relevant snapshot of community values at that point in time. However, responses might have been different if the survey had been conducted at a different time. For example, respondents might have been sensitive to the controversial nature of the topic. They may have been more likely to advocate for correcting injustices if the survey were conducted outside a pandemic.

One limitation is that despite our attempt to obtain a nationally representative sample, in our US sample, Hispanic and Black participants were underrepresented. Thus, our study might underestimate how different racial groups feel about allocation of scarce resources. Furthermore, although we aimed to create and word questions in a neutral and inclusive way, the involvement of Black and BAME community members would have been beneficial in the survey design.

Another limitation is the lack of involvement of non-academic members of the Black and BAME community. Recent frameworks of survey development encourage active involvement of racial minorities in the development and analysis of research as best practice

For peer review only - http://bmjopen.bmj.com/site/about/guidelines.xhtml

to ensure, for instance, that the lived experiences of community members are represented when designing the survey and that different perspectives of how the same data could be interpreted are considered.[45] Hence, it is important to acknowledge that our questions, data collection, and interpretation only represent one perspective and that involvement of non-academic Black and BAME community members might have produced different results.

Conclusion

Obviously, the results of this survey do not settle the ethical question of whether governments should prioritise based on race or other forms of social disadvantage. Ethical argument and analysis may lead to prioritisation policies that differ from those endorsed by the majority. However, understanding the general approach of the public towards prioritisation in settings of severe scarcity may be important in debate and deliberation: it may challenge or confirm assumptions about what the public would or would not support, as well as highlight areas of ethical consensus or point to important areas of disagreement.[46] Our survey identified that in specific allocation scenarios involving high demand and severe resource shortage, a majority of US and UK respondents chose to allocate ventilators to patients with predicted higher chance of survival and vaccines to those at higher risk of severe illness, regardless of patient race. A very small proportion of respondents (<15%) prioritised ventilator allocation to patients from a racial minority, though this was higher among UK respondents from ethnic minority backgrounds. We also found support from a large minority for inclusion of race as a tie-break consideration in vaccine allocation. Although we may hope that it is never needed, these findings may be relevant to planning for the next pandemic.

Contribution statement: AK, HZ, and DW designed the survey, JS, IS, and WS-A provided crucial feedback on methodology and design. HZ organised the collection of the data. AK analyzed the data. AK, HZ, and DW wrote the manuscript, JS, IS, and WS-A provided crucial feedback.

Competing interests: The authors declare that they have no conflicts of interest.

Ethics approval: The study was approved by the University of Oxford Central University Research Ethics Committee [R73841/RE002]. All participants involved provided informed consent.

Data sharing statement: All data, code, and materials used in the analysis can be found at: osf.io/mhyqz

Funding:
This study (survey costs) was commissioned and paid for by the World Health Organization [2020/1077166]. Copyright in the original work on which this article is based belongs to WHO. The authors have been given permission to publish this article.

Researchers were funded in part, by the Wellcome Trust [203132/Z/16/Z] (DW, HZ) and by the Arts and Humanities Research Council (AHRC) (DW) as part of the UK Research and Innovation rapid response to COVID-19 [AH/V013947/1], as well as the NIHR Oxford Health Biomedical Research Centre (IS-BRC-1215-20005) (IS). The funders had no role in

For peer review only - http://bmjopen.bmj.com/site/about/guidelines.xhtml

the preparation of this manuscript or the decision to submit for publication. For the purpose of open access, the authors have applied a CC BY public copyright license to any Author Accepted Manuscript version arising from this submission.

References

1. Wilkinson D. ICU triage in an impending crisis: uncertainty, pre-emption and preparation. *J Med Ethics*. 2020;46(5):287-288. doi:10.1136/medethics-2020-106226

2. Verelst F, Kuylen E, Beutels P. Indications for healthcare surge capacity in European countries facing an exponential increase in coronavirus disease (COVID-19) cases, March 2020. *Eurosurveillance*. 2020;25(13):2000323. doi:10.2807/1560-7917.ES.2020.25.13.2000323

3. Cylus J, Panteli D, van Ginneken E. Who should be vaccinated first? Comparing vaccine prioritization strategies in Israel and European countries using the Covid-19 Health System Response Monitor. *Isr J Health Policy Res*. 2021;10(1):16. doi:10.1186/s13584-021-00453-1

4. Emanuel EJ, Persad G, Kern A, et al. An ethical framework for global vaccine allocation. *Science*. 2020;369(6509):1309-1312. doi:10.1126/science.abe2803

5. WHO SAGE Roadmap For Prioritizing Uses Of COVID-19 Vaccines In The Context Of Limited Supply. Accessed July 15, 2021. https://www.who.int/publications-detail-redirect/who-sage-roadmap-for-prioritizing-uses-of-covid-19-vaccines-in-the-context-of-limited-supply

6. Bubar KM, Reinholt K, Kissler SM, et al. Model-informed COVID-19 vaccine prioritization strategies by age and serostatus. *Science*. 2021;371(6532):916-921. doi:10.1126/science.abe6959

7. Ehni HJ, Wiesing U, Ranisch R. Saving the most lives—A comparison of European triage guidelines in the context of the COVID-19 pandemic. *Bioethics*. 2021;35(2):125-134. doi:10.1111/bioe.12836

8. Savulescu J, Vergano M, Craxì L, Wilkinson D. An ethical algorithm for rationing life-sustaining treatment during the COVID-19 pandemic. *BJA Br J Anaesth*. 2020;125(3):253-258. doi:10.1016/j.bja.2020.05.028

9. Emanuel EJ, Persad G, Upshur R, et al. Fair Allocation of Scarce Medical Resources in the Time of Covid-19. *N Engl J Med*. 2020;382(21):2049-2055. doi:10.1056/NEJMsb2005114

10. Giubilini A, Savulescu J, Wilkinson D. Queue questions: Ethics of COVID-19 vaccine prioritization. *Bioethics*. 2021;35(4):348-355. doi:10.1111/bioe.12858

11. Bibbins-Domingo K, Petersen M, Havlir D. Taking Vaccine to Where the Virus Is—Equity and Effectiveness in Coronavirus Vaccinations. *JAMA Health Forum*. 2021;2(2):e210213-e210213. doi:10.1001/jamahealthforum.2021.0213

For peer review only - http://bmjopen.bmj.com/site/about/guidelines.xhtml

12. Schmidt H, Roberts DE, Eneanya ND. Rationing, racism and justice: advancing the debate around 'colourblind' COVID-19 ventilator allocation. *J Med Ethics*. Published online January 6, 2021:medethics-2020-106856. doi:10.1136/medethics-2020-106856

13. Mathur R, Bear L, Khunti K, Eggo RM. Urgent actions and policies needed to address COVID-19 among UK ethnic minorities. *The Lancet*. 2020;396(10266):1866-1868. doi:10.1016/S0140-6736(20)32465-X

14. White DB, Lo B. Mitigating Inequities and Saving Lives with ICU Triage during the COVID-19 Pandemic. *Am J Respir Crit Care Med*. 2021;203(3):287-295. doi:10.1164/rccm.202010-3809CP

15. Tai DBG, Shah A, Doubeni CA, Sia IG, Wieland ML. The Disproportionate Impact of COVID-19 on Racial and Ethnic Minorities in the United States. *Clin Infect Dis*. 2021;72(4):703-706. doi:10.1093/cid/ciaa815

16. Morales DR, Ali SN. COVID-19 and disparities affecting ethnic minorities. *The Lancet*. 2021;397(10286):1684-1685. doi:10.1016/S0140-6736(21)00949-1

17. Who Gets a Vaccine First? U.S. Considers Race in Coronavirus Plans - The New York Times. Accessed June 24, 2021. https://www.nytimes.com/2020/07/09/us/coronavirus-vaccine.html

18. Jagannathan M. Should Black and Latino people get priority access to a COVID-19 vaccine? MarketWatch. Accessed June 24, 2021. https://www.marketwatch.com/story/should-black-and-latino-people-get-priority-access-to-a-covid-19-vaccine-2020-07-16

19. Sederstrom N. The "Give Back": Is There Room For It? Bioethics.net. Published February 23, 2021. http://www.bioethics.net/2020/07/the-give-back-is-there-room-for-it/

20. Kaposy C, Bandrauk N. Prioritizing Vaccine Access for Vulnerable but Stigmatized Groups. *Public Health Ethics*. 2012;5(3):283-295. doi:10.1093/phe/phs010

21. Schmidt H, Gostin LO, Williams MA. Is It Lawful and Ethical to Prioritize Racial Minorities for COVID-19 Vaccines? *JAMA*. Published online October 14, 2020. doi:10.1001/jama.2020.20571

22. Opinion | Four Ways to Fix the Vaccine Rollout. *The New York Times*. https://www.nytimes.com/2021/01/07/opinion/coronavirus-vaccine-distribution.html. Published January 7, 2021. Accessed July 15, 2021.

23. Essay: Distributive justice during a pandemic – WHYY. Accessed June 24, 2021. https://whyy.org/articles/a-disturbing-medical-consensus-is-growing-heres-what-it-could-mean-for-black-patients-with-coronavirus/

24. Persad G, Emanuel EJ, Sangenito S, Glickman A, Phillips S, Largent EA. Public Perspectives on COVID-19 Vaccine Prioritization. *JAMA Netw Open*. 2021;4(4):e217943. doi:10.1001/jamanetworkopen.2021.7943

For peer review only

25. Williamson EJ, Walker AJ, Bhaskaran K, et al. Factors associated with COVID-19-related death using OpenSAFELY. *Nature*. 2020;584(7821):430-436. doi:10.1038/s41586-020-2521-4

26. [Dataset] Kappes A, Zohny H, Savulescu J, Singh I, Sinnot-Armstrong W, Wilkinson D. Data from: Race and resource allocation: An online survey of US and UK adults' attitudes towards COVID-19. Published online June 1, 2022. osf.io/mhyqz

27. U.S. Census Bureau QuickFacts: United States. Accessed July 22, 2021. https://www.census.gov/quickfacts/fact/table/US/PST045219

28. Wilkinson D, Zohny H, Kappes A, Sinnott-Armstrong W, Savulescu J. Which factors should be included in triage? An online survey of the attitudes of the UK general public to pandemic triage dilemmas. *BMJ Open*. 2020;10(12):e045593. doi:10.1136/bmjopen-2020-045593

29. Butcher B, Massey J. Why are more people from BAME backgrounds dying from coronavirus? *BBC News*. https://www.bbc.com/news/uk-52219070. Published June 19, 2020. Accessed January 31, 2022.

30. Race Disparity Unit. Why we no longer use the term 'BAME' in government. Published online 2022. Accessed September 8, 2022. https://equalities.blog.gov.uk/2022/04/07/why-we-no-longer-use-the-term-bame-in-government/

31. McConahay JB. Modern racism, ambivalence, and the Modern Racism Scale. In: J. F. Dovidio, S. L. Gaertner, eds. *Prejudice, Discrimination, and Racism*. Academic Press; 1986:91-125.

32. Morrison T, Kiss M. Modern Racism Scale. In: Zeigler-Hill V, Shackelford E, eds. *Encyclopedia of Personality and Individual Differences*. Springer International Publishing; 2017.

33. Savulescu J, Persson I, Wilkinson D. Utilitarianism and the pandemic. *Bioethics*. 2020;34(6):620-632. doi:10.1111/bioe.12771

34. Buckwalter W, Peterson A. Public attitudes toward allocating scarce resources in the COVID-19 pandemic. *PLOS ONE*. 2020;15(11):e0240651. doi:10.1371/journal.pone.0240651

35. Grover S, McClelland A, Furnham A. Preferences for scarce medical resource allocation: Differences between experts and the general public and implications for the COVID-19 pandemic. *Br J Health Psychol*. Published online June 20, 2020:e12439. doi:10.1111/bjhp.12439

36. Duch R, Roope LSJ, Violato M, et al. Citizens from 13 countries share similar preferences for COVID-19 vaccine allocation priorities. *Proc Natl Acad Sci*. 2021;118(38). doi:10.1073/pnas.2026382118

37. Song Z, Zhang X, Patterson LJ, Barnes CL, Haas DA. Racial and Ethnic Disparities in Hospitalization Outcomes Among Medicare Beneficiaries During the COVID-19

For peer review only - http://bmjopen.bmj.com/site/about/guidelines.xhtml

Pandemic. *JAMA Health Forum*. 2021;2(12):e214223. doi:10.1001/jamahealthforum.2021.4223

38. Hodge JG, Hanfling D, Hick JL, Piatt JL. Diminishing disparities in U.S. crisis standards of care: Medical and legal challenges. *eClinicalMedicine*. 2021;34. doi:10.1016/j.eclinm.2021.100838

39. Crisp by R. Equality, Priority, and Compassion. *Ethics*. 2003;113(4):745-763. doi:10.1086/373954

40. Kamm FM. Equal Treatment and Equal Chances. *Philos Public Aff*. 1985;14(2):177-194.

41. Ashana DC, Anesi GL, Liu VX, et al. Equitably Allocating Resources during Crises: Racial Differences in Mortality Prediction Models. *Am J Respir Crit Care Med*. 2021;204(2):178-186. doi:10.1164/rccm.202012-4383OC

42. Sjoding MW, Dickson RP, Iwashyna TJ, Gay SE, Valley TS. Racial Bias in Pulse Oximetry Measurement. *N Engl J Med*. 2020;383(25):2477-2478. doi:10.1056/NEJMc2029240

43. Cornelius LJ, Harrington D. *A Social Justice Approach to Survey Design and Analysis*. Oxford University Press; 2014.

44. AISP [Actionable Intelligence for Social Policy]. A Toolkit for Centering Racial Equity Throughout Data Integration. Published online 2019. https://aisp.upenn.edu/centering-equity/

45. Nelson ALH, Zanti S. A Framework for Centering Racial Equity Throughout the Administrative Data Life Cycle. *Int J Popul Data Sci*. 2020;5(3). doi:10.23889/ijpds.v5i3.1367

46. Savulescu J, Gyngell C, Kahane G. Collective Reflective Equilibrium in Practice (CREP) and controversial novel technologies. *Bioethics*. Published online 2021. doi:10.1111/bioe.12869

For peer review only

Figure legends

Figure 1. Survey Design. Participants from the US (N = 1296) or the UK (N = 1284) answered questions about their preferences for allocating scarce medical resources (online sample, aiming to be nationally representative for sex, age, race, household income, and employment – see Supplemental Methods). Fourteen percent of the UK sample, and 19% of the US sample described their racial background as non-White. A: Half the participants (N = 1262) saw scenarios relating to ventilators. They had to decide which of two potential recipients should get the ventilator. Potential recipients had different chances of surviving COVID-19 with ventilation (40%, 50%, or 70%) and one of them had an additional risk factor. Participants could give it to either one of the potential recipients or choose to toss a coin to decide. B: The other half of participants (N = 1318) saw scenarios relating to vaccines. Here, they had to decide which of two potential recipients should get the vaccine. Potential recipients had different chances of developing severe COVID-19 (10%, 15%, or 20%) and one of them had an additional risk factor. Again, participants could give it to either one of the two potential recipients or toss a coin. C: Potential recipients were either individual recipients or groups of recipients. D) Additional risk factors used in our study were racial minority status (Black in the US, BAME in the UK), being male, or being obese. The opposite additional risk factors were White, female, and healthy weight, respectively.

For peer review only - http://bmjopen.bmj.com/site/about/guidelines.xhtml

Figure 2. The role of chances of developing severe COVID-19 for vaccine allocation and chances of survival for ventilator allocations in the absence of additional risk factors. Participants saw either scenarios that involved individual patients (A,C) or groups of patients (B,D). For both individual patients and groups of patients, the greater the difference in chances of survival, the more participants were willing to allocate the ventilator to the potential recipient more likely to survive (A,B). Similarly, the larger the difference in chances of developing severe COVID-19 if infected, the more participants were willing to allocate the vaccine to the potential recipient at higher risk (C,D). (Error bars = 95% Confidence Interval) Note that, in these scenarios (involving only medical risk factors), we gave participants a wider range of outcome variation between recipients than in later scenarios involving additional risk factors. (For symbols, see Fig 1)

Figure 3. (A-C) Preferences for and attitudes towards prioritization of ventilators. A: Percentages of respondents willing to give the ventilator to the racial minority/male/obese patient when both patients had either a similar, or a 10% or 20% higher or lower survival chance. For comparison, we added the responses for scenarios without additional risk factors (white bars, labelled "survival chances"). B: For illustrative purposes, we subtracted the percentage of answers on the scenarios without additional risk factors from those with them. Race and sex did not elicit robust preferences, while obesity elicited negative prioritization preferences. C: Our composite attitude score mirrored the outlined findings. (**D-F) Preferences for and attitudes towards prioritization of vaccines.** D: Percentages of respondents willing to give the vaccine to the recipient with an additional risk factor when their chances of developing severe COVID-19 were either the same or were 5% or 10% higher or lower than the recipient without an additional risk factor. For comparison, we added the answers for scenarios without additional risk factors (White bars: labelled "Covid risk"). E: For illustrative purposes, we subtracted the percentage of answers on the scenarios without additional risk factors from those that contain them. A clear positive preference for each factor emerged, with obesity eliciting the strongest positive responses. F: Our composite attitude score mirrored the outlined finding. For vaccines, each factor elicited a positive preference, obesity being strongest. (Error bars = 95% Confidence Interval)

For peer review only - http://bmjopen.bmj.com/site/about/guidelines.xhtml

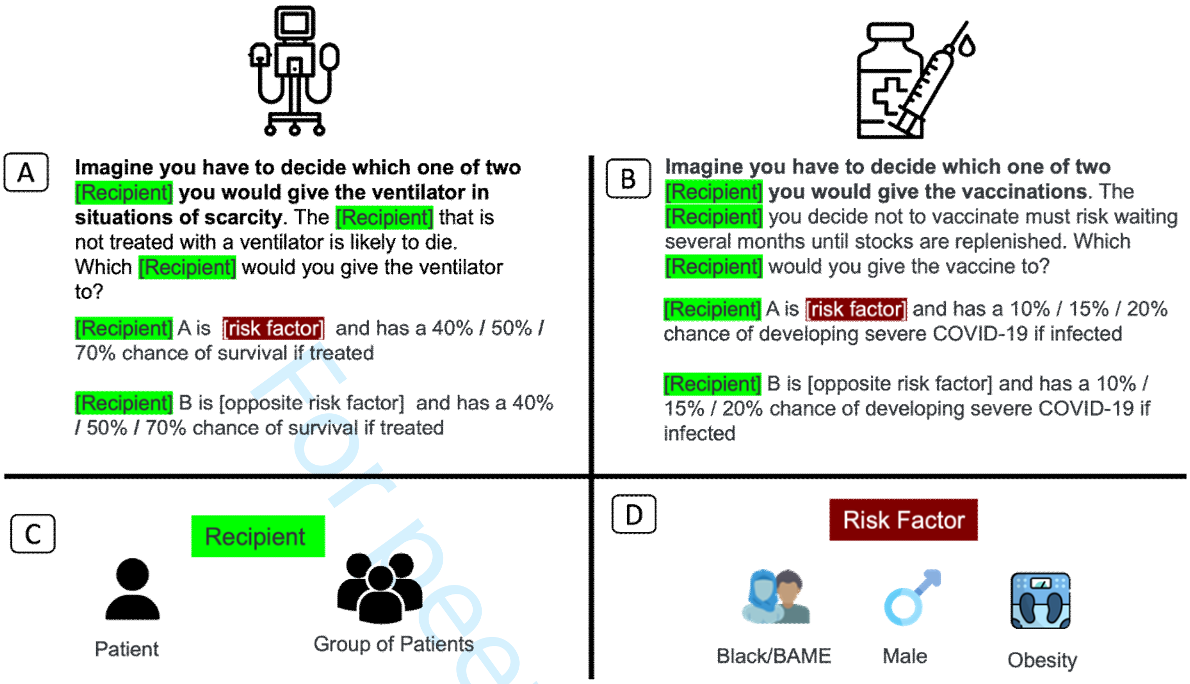

For peer review only

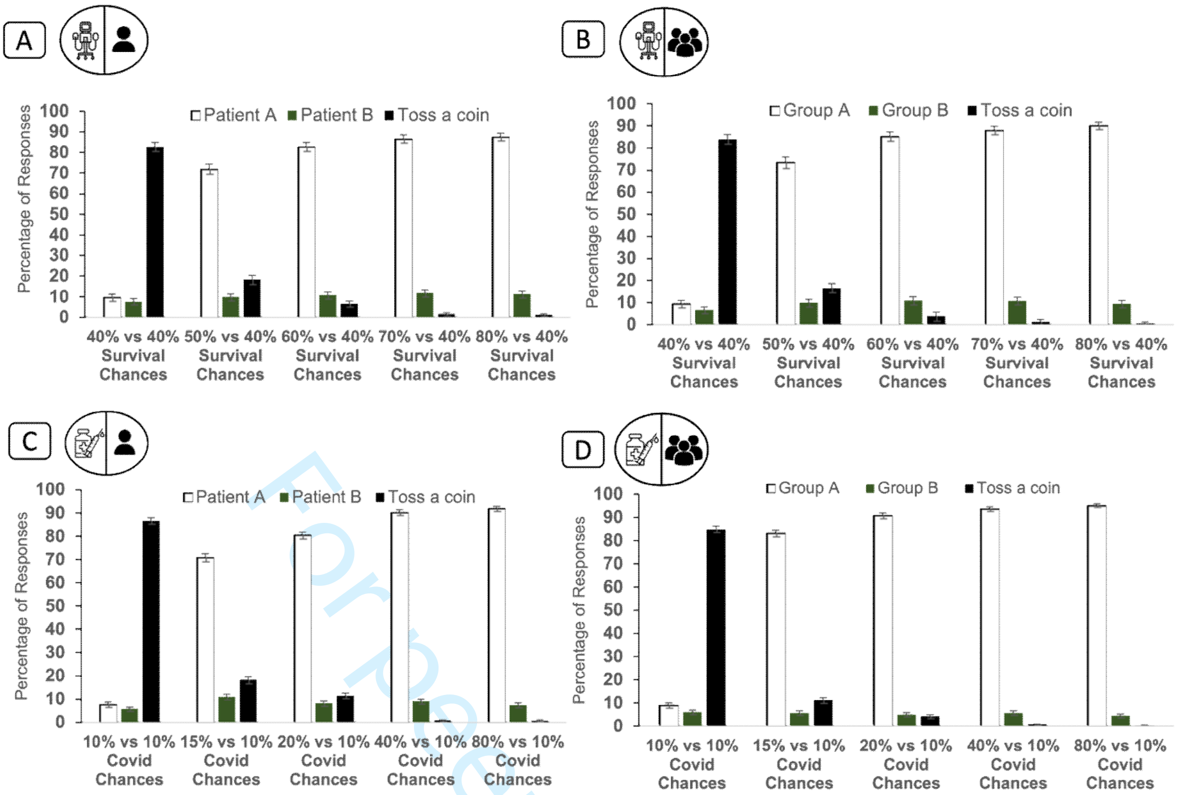

For peer review only

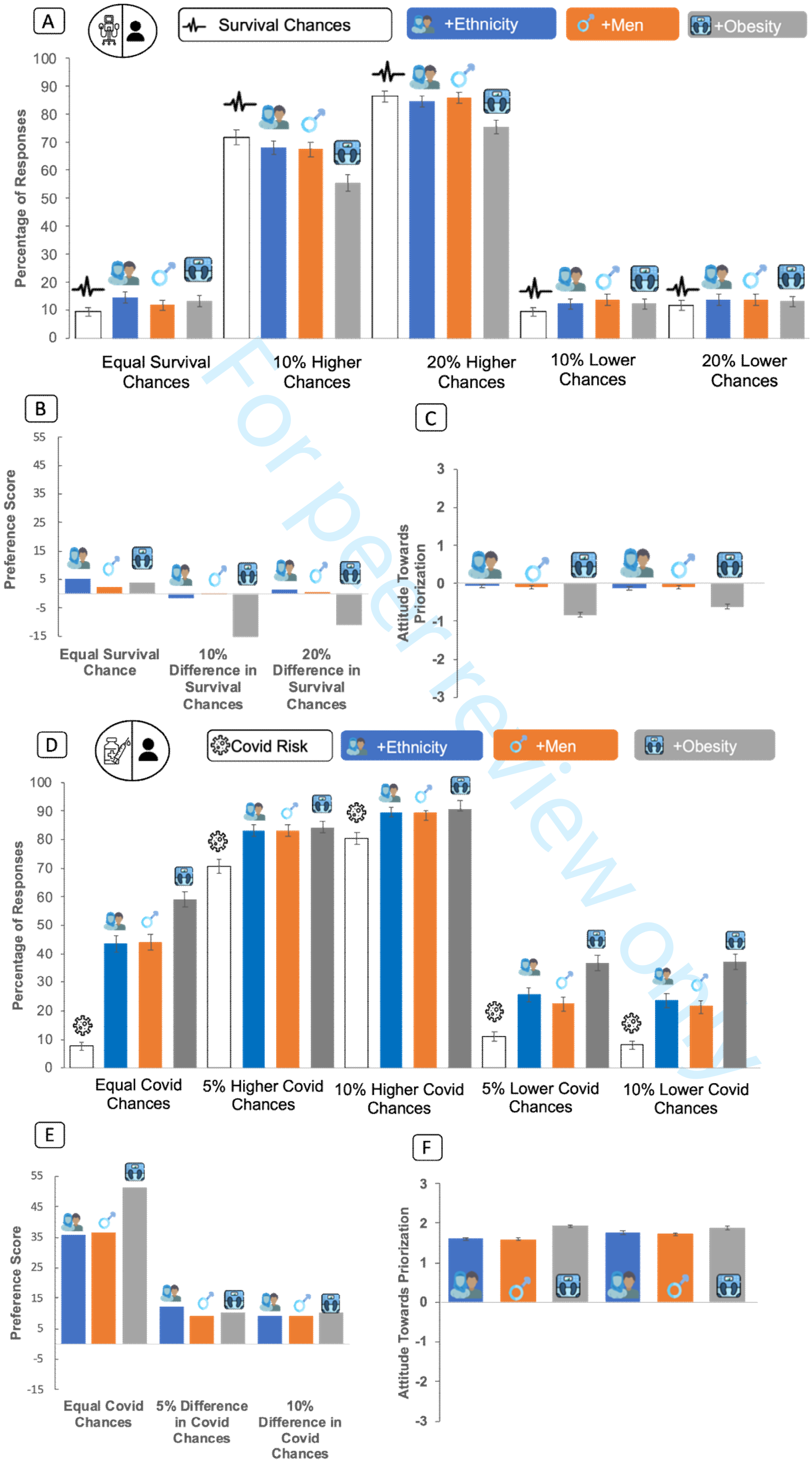

For peer review only

For peer review only

Supplemental Materials: Public attitudes towards race and resource allocation in a pandemic

Andreas Kappes[1], Hazem Zohny[2], Julian Savulescu[2,3], Ilina Singh[4 5], Walter Sinnott-Armstrong[6], Dominic Wilkinson[2,3,7]

Affiliations:
[1]City, University of London
[2] Oxford Uehiro Centre for Practical Ethics, University of Oxford
[3] Murdoch Children's Research Institute, Melbourne, Australia.
[4] Wellcome Trust Centre for Ethics and the Humanities, University of Oxford
[5] Department of Psychiatry, University of Oxford
[6] Kenan Institute for Ethics and Philosophy Department, Duke University
[7] John Radcliffe Hospital, Oxford, UK

Corresponding author Email: hazem.zohny@philosophy.ox.ac.uk

For peer review only

Supplemental Methods

Procedure

The survey was designed to assess participants' preferences for using medical factors as well as race, sex, obesity for allocating vaccines to people in need. Two surveys were conducted, one in the US and one in the UK. The surveys were adapted from a previous survey (4) on triage guidance involving ventilator allocations. Full survey materials can be found here: osf.io/w7bpq.

Vaccine Allocations

All participants were first introduced to the context of the allocation decisions. We wanted to make sure that all participants knew what would happen to the recipient that would not get vaccine, that all recipients wanted to get the vaccine, and that the vaccine was effective and safe. Participants read:

> *For the following scenarios, imagine you are working in a clinic. You are in charge of deciding which patients should get vaccinated first against COVID-19. You have one remaining dose of the vaccine left, and you must choose between two patients. The unvaccinated patient must wait several months before stocks are replenished. For the purpose of this survey, please think of the vaccine as if it had a 100% success rate in preventing COVID-19 and no side effects whatsoever. And each person under consideration wants to get vaccinated.*

Participant then learned that the estimate for each recipient's chance of getting severe COVID-19 was high and was based on their age and medical history. Furthermore, they learned that their task was to decide whom to give the vaccine.

Risk of developing severe COVID-19. To determine how much weight participants would give the risk of developing severe COVID-19 for vaccine allocations, participants answered five scenarios in which the potential recipients only differed in their chance of developing severe COVID-19. In one scenario, one patient had an 80% chance and the other one a 10% chance of developing severe COVID-19 (other scenarios: 40% versus 10%, 20% versus 10%, 15% versus 10% and 10% versus 10%). Thereafter, participants were randomly introduced to one of the three additional factors: race, sex, and obesity.

Racial minority scenarios. For a racial minority in the US, we chose Black recipients and, in the UK, BAME recipients. BAME is an umbrella term, common in the United Kingdom, used to describe non-white racial groups. While a contested term, it was most consistently used in media reports on the pandemic in the UK (e.g., 5). Before participants had to decide if they wanted to give the vaccine to the member of the racial minority, we ensured that all participants were aware of the inequality in COVID-related deaths in the UK and in the US. UK participants read: "*According to the Office for National Statistics, individuals belonging to Black, Asian, and minority ethnic (BAME) groups have in general been shown to die from COVID-19 at a higher rate than that of White individuals.*" US participant read: "*According to the Centre for Disease Control and Prevention, Black Americans have been shown to die from COVID-19 at a higher*

For peer review only - http://bmjopen.bmj.com/site/about/guidelines.xhtml

rate than of White Americans." Thereafter, participants worked on five scenarios. All of these scenarios involved one Black (BAME) and one White patient. In one scenario, both patients had the same risk of illness (10% versus 10%), in two scenarios the Black (BAME) patient had a higher risk of severe COVID-19 (5% and 10% higher), and in two scenarios the White patient a higher risk (5% and 10% higher). Participants indicated for each whether they wanted to give the vaccine to the racial minority patient, the white patient, or toss a coin.

Sex and obesity scenarios. Participants saw a similar set of scenarios for sex-based and obesity-based allocation decisions. Before working on the scenarios that involved men and women, participants read: "*According to scientific studies, men have been shown to die from COVID-19 at a higher rate than that of women."* The rationale was to inform participants that being male may be predictive of a worse outcome or survival chance if infected with COVID-19 (6). And before working on the scenarios involving obesity, participants read: "*According to scientific studies, people who are obese (severely overweight) have been shown in general to die from COVID-19 at a higher rate than that of people who have a healthy weight."* The rationale for presenting participants with this was to inform them about obesity being predictive of a worse outcome or survival chance if infected with COVID-19 (7). The five obesity-related and five sex-related scenarios followed the exact same structure as the race-related scenarios.

Group scenarios. To test if it would make a difference if participants considered only one patient, or a group of patients, we repeated the same set of scenarios for race, sex, and obesity for groups of patients. The reason for including group scenarios was twofold. First, there is a large body of evidence that suggest that people have different moral intuitions when they think about one persons versus a group of people in need (8). Second, one might argue that people might feel that the single recipient scenario for vaccine distributions is unrealistic since such decisions most often are made for large groups, rather than individuals. To ensure that such perceptions did not impact allocation preferences, we additionally included group scenarios. The scenarios followed the exact same structure as the individual ones but instead of deciding between two patients, participants had to decide between two groups of patients. As an introduction to this section of the survey, participants were told:

> *Imagine you are a policymaker for the government. At the moment, the number of vaccines available is limited and you do not have enough to protect everyone. Sometimes, policy makers face difficult decisions about which subsets of the population should be given a vaccine and which shouldn't. You will make decisions about whether or not to provide vaccines to one of two groups of people. If the people in this survey are not vaccinated, they are likely to get COVID-19 and suffer serious health consequences as a result. The group you decide not to vaccinate must risk waiting several months until stocks are replenished.*

Race, sex, and obesity scenarios. The structure of each of these scenario groups followed the structure for the respective vaccine scenario. Participants were informed about the effect race, sex, and obesity had on COVID-related deaths before working on the corresponding set of scenarios (see above). In the five race-related scenarios, participants both patients had the same chances of survival (40% versus 40%), in two scenarios the Black (BAME) patient had a higher chance of survival (10% and 20% higher), and in two scenarios the White patient a higher risk

For peer review only - http://bmjopen.bmj.com/site/about/guidelines.xhtml

chance of survival (10% and 20% higher). Participants indicated for each whether they wanted to give the vaccine to the racial minority patient, the white patient, or toss a coin. The scenarios related to sex and obesity followed the exact structure as the race-related scenarios.

Prioritization Attitude Measure. For each of the sets of scenarios, we created an attitude score by counting the number of times participants decided to give the vaccine to the person with an additional risk factor on each of the five respective scenarios. For instance, a participant that would always give the vaccine to the recipient from the racial minority would have an attitude score of 5, a participant who would allocate solely by the risk of contracting severe COVID-19 would get a 0, and a person that would always give priority to the white recipient would get a score of -5. We did this for all the patient and all the group scenarios.

Perceptions of Injustice, Responsibility, Stereotypes, and Racism

After participants worked on the scenarios, we asked them a series of questions to capture their perceptions of racial minorities, men, and obese people.

Perceptions of injustice, responsibility. To measure as how unjust people perceived the higher COVID-related death rates, we asked participants to indicate their agreement with the following statement: "Injustice was the main reason [category] individuals died more often than individuals from other groups". To measure perceptions of responsibility of the group for the higher death rates, we asked participants to indicate their agreement with the following statement: "The main reason [category] die more often from COVID-19 is due to factors outside their control." For both questions, scales ranged from 1 (strongly disagree) to 5 (strongly agree).

Stereotypes. According to the stereotype content model, perceptions of groups can be described along two fundamental dimensions: warmth and competence (9, 10). In line with previous research, we measured warmth by how warmly participants felt towards black (US) or BAME (UK) people, men, and obese people. We measured competence by asking participants how competent they perceived black (US) or BAME (UK) people, men, and obese people to be. We used 100-point scales for participants to indicate their perceptions.

Covert Racism - Modern Racism Scale. The modern racism scale is intended to capture subtle or covert racial attitudes or the endorsement of the idea that racial minorities are getting undeserved attention (1). Although modern racist potentially do not perceive themselves as racist, attitudes guide their decisions in situations that provide nonracially motivated reasons. Hence, the modern racism scale provides seven statements that one could endorse or reject (e.g., "Discrimination against Blacks is no longer a problem in the United States" and "Over the past few years, Blacks have gotten more economically than they deserve"). Participants are asked to express their agreement of each statement via a 5-point scale, ranging from 1 (Strongly disagree) to 5 (Strongly agree). Extensive previous research has found that scores on the modern racism scale correlate with anti-Black affect, right-wing authoritarianism, social dominance orientation, and indices of old-fashioned racism (11).

Statistical Analyses (Fixed Linear Models)

For peer review only - http://bmjopen.bmj.com/site/about/guidelines.xhtml

We ran a series of 4 linear models to explore the role of various factors in predicting racial prioritization attitudes. For each model, we defined attitudes as dependent variable and subjects as the random factor. In the first two models, we entered age, gender, race, relative income, educational level, political ideology (general, social, and economical), and type of scenario (patient versus group-based) as fixed factors. In the second two models, we additionally added perceptions of injustice, responsibility, warmth, competence, and modern racism scores (see results above).

Supplemental Results

1. Group Preferences for Vaccine and Ventilator Preferences

Patterns of response were similar for scenarios involving groups as for individual patients (Figure 2 for individual preferences, Figure S1 for group preferences).

For peer review only

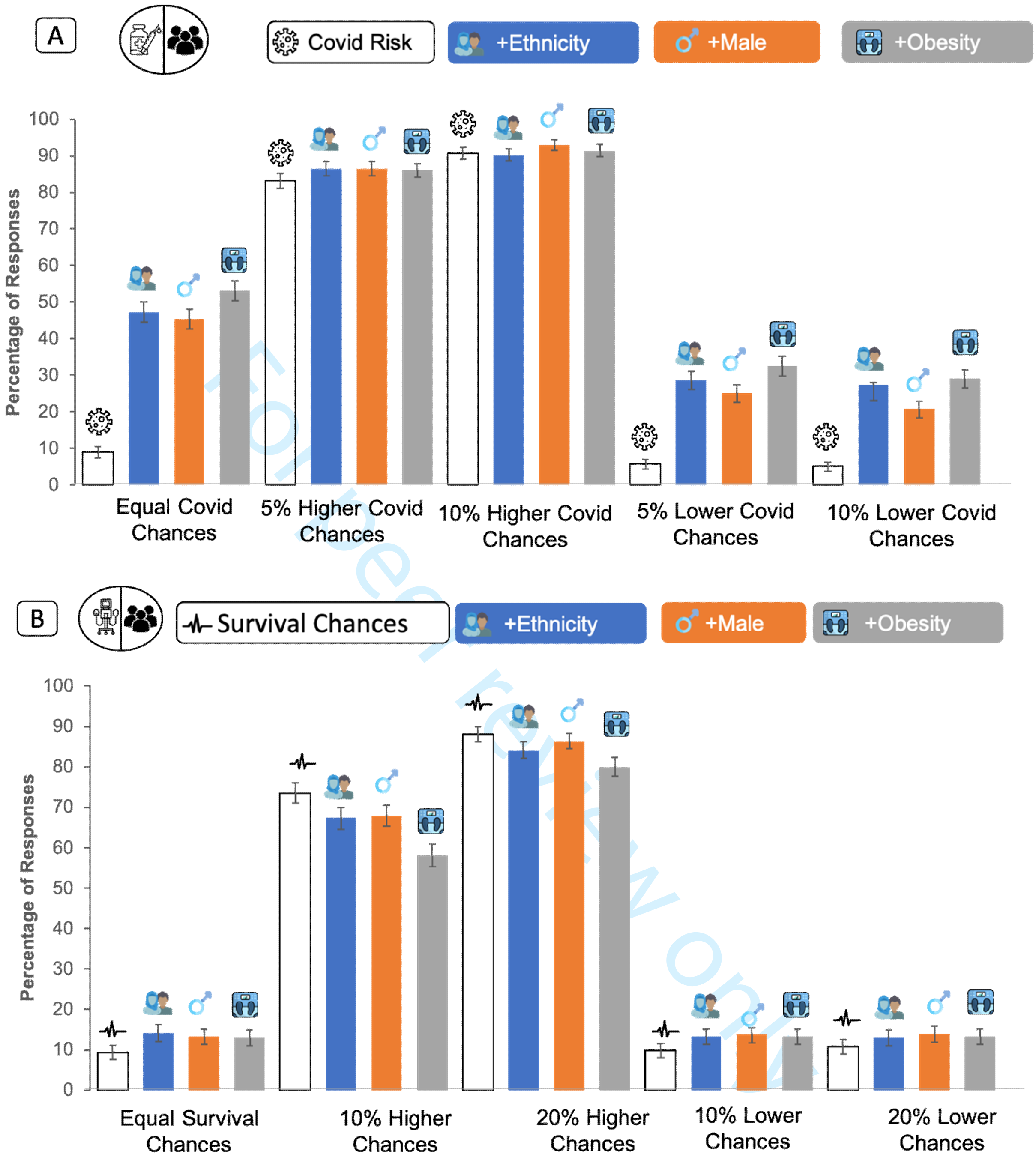

Supplementary Figure S1. A: Preferences for prioritization of vaccines. Response to scenarios where competing individual patients differed in medical risk alone or in medical risk plus an additional risk factor (race, sex, or obesity), where the patient with the additional risk factor had a 5% or 10% higher or lower chance of getting severe Covid based on medical risk alone. The bars indicate the proportion of respondents who would allocate the vaccine to a patient with a particular characteristic and risk profile. **B: Preferences for prioritization of ventilators.** A: Response to scenarios where competing individual patients differed in medical risk alone (Equal COVID chance), or in medical risk plus an additional risk factor (race, sex, or obesity), where the patient with the additional risk factor had a 10% or 20% higher or lower chance of dying based on medical risk alone. The bars indicate the proportion of respondents who would allocate the ventilator to a patient with a particular characteristic and risk profile.

For peer review only

3. Responses of Black American participants for all scenarios involving ventilators and race in true percent

	Black American Participants		
	Black Patient	Coin Toss	White Patient
Equal Survival Chances	29.5	52.5	18.0
10% Higher Chance	55.7	18.0	26.2
30% Higher Chance	62.3	1.6	31.1
10% Lower Chance	32.8	13.1	54.1
30% Lower Chance	34.4	3.3	62.3

4. Responses of Black American participants for all scenarios involving vaccines and race true precent

	Black American Participants		
	Black Patient	Coin Toss	White Patient
Equal Covid Chances	49.1	41.5	9.4
5% Higher Chance	86.8	1.9	11.3
10% Higher Chance	86.8	1.9	11.3
5% Lower Chance	35.8	3.8	60.4
10% Lower Chance	34	1.9	64.2

5. General Linear Mixed Models

To test which variables impacted participants' attitudes towards risk factors, we ran a series of four linear models to explore the role of various factors in predicting racial prioritization attitudes. For each model, we defined attitudes as dependent variable and subjects as the random factor. In the first two models, we entered age, gender, race, relative income, educational level, political ideology (general, social, and economical), and type of scenario (patient versus group-based) as fixed factors. In the second two models, we additionally added perceptions of injustice, responsibility, warmth, competence, and modern racism scores. The full results of each model can be seen in Table S1.

Table S1. Linear Mixed Models

Information Criteria	US: Vaccination Simple Model	US: Vaccination Full Model	UK: Vaccination Simple Model	UK: Vaccination Full Model
-2 Log Likelihood	5253.189	4906	4846.48	4382.353
Akaike's Information Criterion (AIC)	5305.189	4968	4892.48	4438.353

For peer review only - http://bmjopen.bmj.com/site/about/guidelines.xhtml

Hurvich and Tsai's Criterion (AICC)	5306.273	4970	4893.34	4439.724
Bozdogan's Criterion (CAIC)	5466.048	5158	5034.64	4609.2
Schwarz's Bayesian Criterion (BIC)	5440.048	5127	5011.64	4581.2

**Information Criteria**	**US: Ventilator Simple Model**	**US: Ventilator Full Model**	**UK: Ventilator Simple Model**	**UK: Ventilator Full Model**
-2 Log Likelihood	4793.874	4733.97	3845.598	3758.101
Akaike's Information Criterion (AIC)	4847.874	4797.97	3893.598	3816.101
Hurvich and Tsai's Criterion (AICC)	4849.092	4799.68	3894.575	3817.529
Bozdogan's Criterion (CAIC)	5013.837	4994.616	4040.817	3993.85
Schwarz's Bayesian Criterion (BIC)	4986.837	4962.616	4016.817	3964.85

American Sample: Vaccination Allocation

	**Numerator df**	**Denominator df**	**F**	**Sig.**
Intercept	1	661	0.129	0.72
Scenario Type (Group or Patient)	1	661	63.748	< .0001
Age	1	661	0.408	0.523
Race (US)	5	661	1.706	0.131
Gender	3	661	7.238	< .0001
Highest Schooling	8	661	1.157	0.323
Social Status	1	661	0.089	0.765
Household Income	1	661	0.726	0.394
Political Orientation (General)	1	661	3.32	0.069
Political Orientation (Economical)	1	661	0.526	0.469
Political Orientation (Social)	1	661	11.382	0.001

UK Sample: Vaccination Allocation

For peer review only

	Numerator df	Denominator df	F	Sig.
Intercept	1	657	1.544	0.214
Scenario Type (Group or Patient)	1	657	91.289	< .0001
Age	1	657	0.233	0.63
Race (UK)	5	657	1.212	0.302
Gender	1	657	0.272	0.602
Highest Schooling	7	657	0.762	0.62
Social Status	1	657	1.985	0.159
Household Income	1	657	0.013	0.909
Political Orientation (General)	1	657	0	0.988
Political Orientation (Economical)	1	657	0.138	0.71
Political Orientation (Social)	1	657	0.244	0.621

American Sample: Vaccination – Full Model

	Numerator df	Denominator df	F	Sig.
Intercept	1	621	0.371	0.543
Scenario Type (Group or Patient)	1	621	61.082	< .0001
Age	1	621	1.171	0.28
Race (US)	5	621	1.328	0.25
Gender	3	621	4.702	0.003
Highest Schooling	8	621	0.678	0.711
Social Status	1	621	0.16	0.689
Household Income	1	621	0.096	0.756
Political Orientation (General)	1	621	5.125	0.024
Political Orientation (Economical)	1	621	0.008	0.927
Political Orientation (Social)	1	621	6.729	0.01
Injustice Perception	1	621	0.641	0.424
Responsibility Perception	1	621	2.589	0.108
Warmth Perception	1	621	0.012	0.912
Competence Perception	1	621	1.488	0.223

For peer review only - http://bmjopen.bmj.com/site/about/guidelines.xhtml

Modern Racism	1	621	23.16	< .0001

UK Sample: Vaccination – Full Model

	Numerator df	Denominator df	F	Sig.
Intercept	1	607	0.809	0.369
Scenario Type (Group or Patient)	1	607	89.533	< .0001
Age	1	607	3.054	0.081
Race (UK)	1	607	0.023	0.879
Gender	7	607	1.176	0.315
Highest Schooling	1	607	2.438	0.119
Social Status	1	607	0.091	0.763
Household Income	1	607	0.304	0.582
Political Orientation (General)	1	607	0.171	0.68
Political Orientation (Economical)	1	607	1	0.318
Political Orientation (Social)	5	607	0.96	0.442
Injustice Perception	1	607	0.027	0.87
Responsibility Perception	1	607	14.238	< .0001
Warmth Perception	1	607	0.048	0.826
Competence Perception	1	607	7.638	0.006
Modern Racism	1	607	16.986	< .0001

American Sample: Ventilator

	Numerator df	Denominator df	F	Sig.
Intercept	1	635	4.907	0.027
Scenario Type (Group or Patient)	1	635	2.418	0.12
Age	1	635	1.195	0.275
Race (US)	6	635	1.386	0.218
Gender	3	635	3.6	0.013
Highest Schooling	8	635	1.795	0.075
Social Status	1	635	4.437	0.036

For peer review only - http://bmjopen.bmj.com/site/about/guidelines.xhtml

Household Income	1	635	12.582	0
Political Orientation (General)	1	635	0.043	0.836
Political Orientation (Economical)	1	635	0.011	0.917
Political Orientation (Social)	1	635	0.976	0.324

UK Sample: Ventilator

	Numerator df	Denominator df	F	Sig.
Intercept	1	627	12.266	0
Scenario Type (Group or Patient)	1	627	1.713	0.191
Age	1	627	5.755	0.017
Race (UK)	4	627	7.181	0
Gender	3	627	0.342	0.795
Highest Schooling	7	627	0.521	0.819
Social Status	1	627	0.514	0.474
Household Income	1	627	0.816	0.367
Political Orientation (General)	1	627	0.793	0.373
Political Orientation (Economical)	1	627	0.002	0.962
Political Orientation (Social)	1	627	0.26	0.611

US Sample: Ventilator

	**Numerator df**	**Denominator df**	**F**	**Sig.**
Intercept	1	634	2.806	0.094
Scenario Type (Group or Patient)	1	634	2.418	0.12
Age	1	634	0.757	0.385
Race (UK)	6	634	0.897	0.497
Gender	3	634	3.411	0.017
Highest Schooling	8	634	1.624	0.115
Social Status	1	634	3.699	0.055

For peer review only - http://bmjopen.bmj.com/site/about/guidelines.xhtml

Household Income	1	634	11.868	0.001
Political Orientation (General)	1	634	0.039	0.843
Political Orientation (Economical)	1	634	0.785	0.376
Political Orientation (Social)	1	634	0.047	0.829
Injustice Perception	1	634	0.778	0.378
Responsibility Perception	1	634	0.382	0.537
Warmth Perception	1	634	1.014	0.314
Competence Perception	1	634	9.688	0.002
Modern Racism	1	634	10.737	0.001

**UK Sample: Ventilator**	**Numerator df**	**Denominator df**	**F**	**Sig.**
Intercept	1	624	1.419	0.234
Scenario Type (Group or Patient)	1	624	1.926	0.166
Age	1	624	1.316	0.252
Race (UK)	3	624	0.864	0.46
Gender	7	624	1.241	0.278
Highest Schooling	1	624	0.066	0.797
Social Status	1	624	0.913	0.34
Household Income	1	624	0.096	0.757
Political Orientation (General)	1	624	0.038	0.846
Political Orientation (Economical)	1	624	0.59	0.443
Political Orientation (Social)	4	624	4.889	0.001
Injustice Perception	1	624	2.732	0.099
Responsibility Perception	1	624	0.744	0.389
Warmth Perception	1	624	3.129	0.077
Competence Perception	1	624	4.65	0.031
Modern Racism	1	624	13.881	< .0001

6. Perceptions of Injustice, Responsibility, and Stereotypes

In the US and the UK, participants were more likely to believe that adverse COVID-19 related health outcomes were the result of injustice and outside the individual's control for race than for obesity/male sex [Figure S2]. In particular, injustice was perceived differently for the three additional risk

For peer review only - http://bmjopen.bmj.com/site/about/guidelines.xhtml

factors (F(2, 2577)=217.388, $P < 0.0001$, partial eta^2 =0.144). Bonferroni-corrected posthoc comparisons indicated that participants most strongly attributed the increased numbers of deaths of racial minorities due to injustice, stronger than the deaths of men ($P < .0001$, 95% CI: 0.372-0.469), and obese people ($P < 0.000$, 95% CI: 0.300-0.399). For the latter two, participants indicate more injustice to obese people death's than to men ($P < .0001$, 95% CI: 0.035-0.108). Similarly, there were differences in perceived personal control for adverse outcomes from COVID (F(2, 2577)=284.123, $P < .0001$, partial eta^2 = .181). Racial minorities were perceived to have the least control over the higher death rate compared to men (P =0.009, CI 95: 0.011-0.103) and obese people ($P < .0001$, 95% CI:0.529-0.655). Obese people were perceived to have most control over the higher death rates attributable to COVID, higher control than men ($P < .0001$, 95% CI: 0.595-0.476) and people from racial minorities (P =0.009, 95% CI: 0.011-0.103).

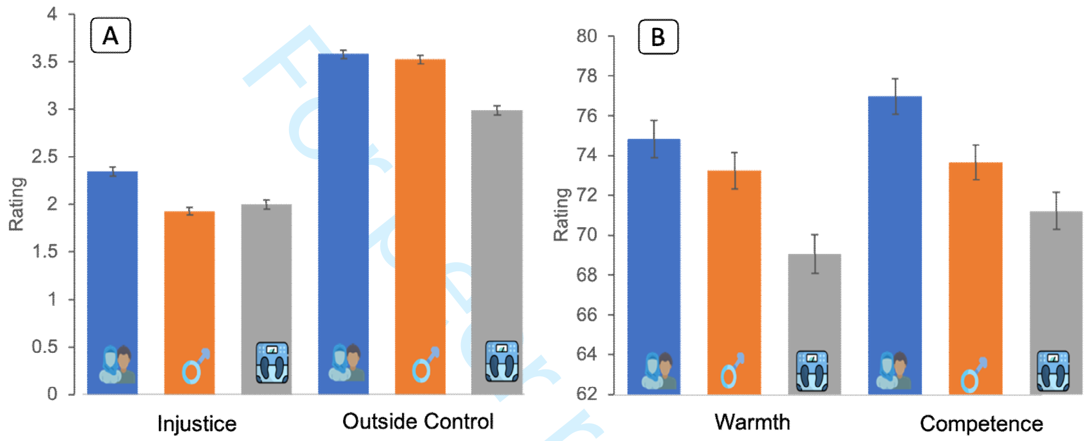

Supplementary Figure 2: Perceptions of Injustice, Control, Warmth, and Competence. A: Bonferroni-corrected comparisons indicated that participants most strongly attributed the increased numbers of deaths of racial minorities to injustice, stronger than the deaths of males ($P < .0001$, 95% CI: 0.372-0.469), and obese people ($P < 0.000$, 95% CI: 0.300-0.399). Participants attributed slightly more injustice to obese people's deaths than to males' deaths ($P < .0001$, 95% CI: 0.035-0.108). Similarly, racial minorities were perceived to have less control over the higher death rate compared to males (P =0.009, CI 95: 0.011-0.103) and obese people ($P < .0001$, 95% CI:0.529-0.655). Obese people were perceived to have the most control over higher death rates attributable to COVID-19, more than males ($P < .0001$, 95% CI: 0.595-0.476) and racial minorities (P =0.009, 95% CI: 0.011-0.103). B: Survey participants had the strongest stereotype content feelings of warmth towards ethical minorities, compared to attitudes towards males (p =0.005, Bonferroni corrected, small difference) and obese people ($P < 0.0001$, Bonferroni corrected). Participants also expressed higher feelings of competence toward individuals from a racial minority than males ($P < 0.0001$, Bonferroni corrected), or obese people ($P < 0.0001$, Bonferroni corrected). (Error bars = 95% Confidence Interval).

How did these perceptions of injustice and responsibility differ between the US and the UK? For injustice, we found no main effect of country (F(1,2576) = 2.534, P =.112, partial eta^2 =.001) and no interaction effect with categories of disadvantage (F(1,2576) = 1.622, P =0.203, partial eta^2 =.001). Participants in both countries perceived the relative injustice that affected each group similarly. When examining responsibility, we found no main effect of country (F(1,2576) = 0.366, P =0.545, partial eta^2 = 0.00001), but an interaction between country and category (F(1,2576) =31.651, $P < .0001$, partial eta^2 = 0.012). Participants in the US gave men less responsibility (t(2578) = 4.091, $P < 0.0001$), but obese people more responsibility (t(2578) = 4.176 .000, $P < 0.0001$) than participants in the UK.

Perceptions of injustice and responsibility might be related to the general attitudes towards a particular category. Attitudes can be characterized via two different dimensions, warmth and competence[56,57]**.** We found that again, respondents had different feelings of warmth towards different

For peer review only - http://bmjopen.bmj.com/site/about/guidelines.xhtml

categories (F(1, 2577) = 72.446, P <0.0001, partial eta^2 = .029) and competence (F(1, 2577) = 76.369, P < 0.0001, partial eta^2 =0.030). In particular, survey participants had strongest feelings of warmth towards ethical minorities, compared to feelings towards men (p =0.005, Bonferroni corrected, small difference) and obese people (P < 0.0001, Bonferroni corrected), and rated the warmth of obese people lower than the warmth of men (P < 0.0001, Bonferroni corrected). When examining competence, we found a similar effect of group membership (F(2,2575) = 77.00, P < 0.0001, partial eta^2 = .03). Participants expressed higher feelings of competence in individuals from a racial minority than men (P < 0.0001, Bonferroni corrected), or obese people (P < 0.0001, Bonferroni corrected), while men were perceived as more competent than obese people (P < 0.0001, Bonferroni corrected).

Again, one might wonder whether participants from the US and the UK had different stereotypes about racial minorities, men, and obese people. When examining the effect of country on warmth, we find a main effect of country, F(1,2497) = 19.408, P < 0.0001, partial eta^2 = .008, American participants gave higher warmth ratings overall, though this varied between categories, F(2,2497) = 15.083, P < .0001, partial eta^2 = 0.006. The biggest difference was in warmth perception of obese participants, with American participants rating obese people more warmly than British participants (t(2495) = 5.73, P < .001), and only very small differences for the other two categories between both countries. When examining the effect of country and competence ratings, a similar picture emerged. We found a main effect for country (F(2,2497) = 25.16, P <.0001 , partial eta^2 = .01, which was qualified by an interaction effect with disadvantage categories, F(2,2497) = 17.49, P <001 , partial eta^2 = .007). Again, the main difference was for perceptions of obese people, Americans perceived obese people as more competent than British people, t(2497) = 6.672, P < .0001).

Why do people perceive the effect of a specific additional risk factor such as race as unjust? Part of the reason might be because of how favorably one thinks about the additional risk factor in general. To test this idea, we regressed feelings of responsibility, warmth, and competence on perceptions of injustice for each of the three additional risk factors. For race, we found that perceptions of responsibility (beta = .130, t(2482) = 6.551, P <.0001) and feelings of warmth, (beta = .209, t(2482) = 6.89, P < .0001) predicted the perception of injustice, but not perceptions of competence (beta = -.039, t(2482) = 1.309, P =0.191). For men, neither responsibility (beta = .032, t(2482) = 1.589,P = 0.112), competence (beta-.054, t-1.930 P = 0.054), or warmth (beta = .035, t1.262, P = 0.207) predicted perceptions of injustice. In contrast, for obesity, responsibility (beta = .222, t(2482) = 11.047, P < 0.0001), warmth (beta = .103, t(2482) = 3.587, P < 0.0001), and competence (beta = -.074, t(2482) =2.624, P = 0.009) predicted perceptions of injustice.

4. Allocation attitudes for participants with low and high modern racism scores

In the US and the UK, responses on the Modern Racism Scale were associated with attitudes towards ventilator and vaccine allocation based on race (Ventilator: US: F(1,634) = 10.73, p = 0.001, UK: F(1,624) = 13.88, P < .0001; Vaccine: US: F(1,621 =23.00, P <0.0001, UK: F(1,607)=16.98, P <0.0001). The more participants endorsed modern racism statements, the less positive their composite attitudes towards ventilator or vaccine allocation to racial minority patients. A minority (27.3%) of participants had a mean score of higher than three on the modern racism scale, indicating an endorsement of modern racism. These participants did not give weight to race in ventilator or vaccine allocations [SM, Figure 3]. Participants with low modern racism scores also did not give weight to race in ventilator allocation but did for vaccines. Modern racism did not affect prioritisation based on sex/obesity [SM Figure 3]. Perceptions of injustice did not predict ventilator or vaccine allocation attitudes in the US (F(1,634) = 0.778, P = 0.378) or the UK (F(1,624) = 2.732, P = 0.099).

For peer review only - http://bmjopen.bmj.com/site/about/guidelines.xhtml

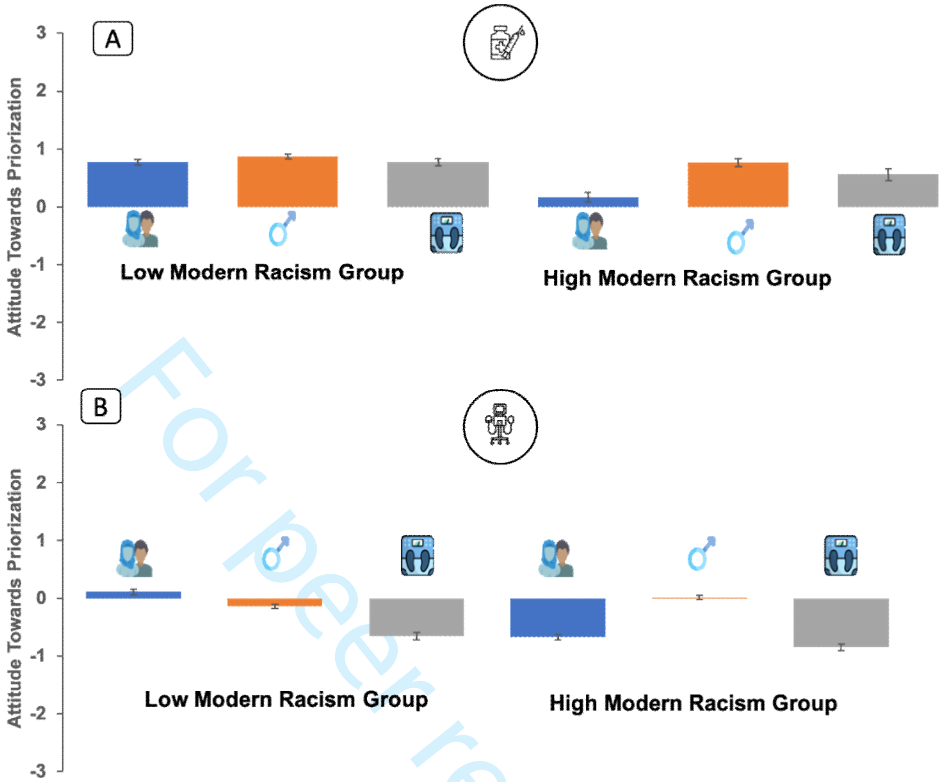

Supplementary Figure 3: Allocation attitudes for participants with low modern racism scores (Low Modern Racism Group) and high modern racism scores (High Modern Racism Group). A: For vaccines, participants in the Low Modern Racism Group (mean score lower than scale midpoint, N = 1011) had a more positive attitude towards race-based vaccine allocation compared to participants in the High Modern Racism Group (mean score higher than scale midpoint, N = 307). Allocation attitudes for sex and obesity were similar. B: For ventilators, we find that participants in the Low Modern Racism Group (N = 919) had a neutral attitude towards race-based vaccine allocation, while participants in the High Modern Racism Group (N = 343) had a negative one. Allocation attitudes for sex and obesity were similar.

References (Supplemental Materials)

1. J. B. McConahay, "Modern racism, ambivalence, and the Modern Racism Scale" in *Prejudice, Discrimination, and Racism*, J. F. Dovidio, S. L. Gaertner, Eds. (Academic Press, 1986), pp. 91–125.

2. T. G. Morrison, M. Kiss, Modern racism scale. *Encyclopedia of personality and individual differences*, 1–3 (2017).

3. , U.S. Census Bureau QuickFacts: United States (July 22, 2021).

4. D. Wilkinson, H. Zohny, A. Kappes, W. Sinnott-Armstrong, J. Savulescu, Which factors should be included in triage? An online survey of the attitudes of the UK general public to pandemic triage dilemmas. *BMJ Open* **10**, e045593 (2020).

For peer review only

5. , Why are more people from BAME backgrounds dying from coronavirus? *BBC News* (2020) (June 28, 2021).

6. T. Takahashi, *et al.*, Sex differences in immune responses that underlie COVID-19 disease outcomes. *Nature* **588**, 315–320 (2020).

7. J. Yang, J. Hu, C. Zhu, Obesity aggravates COVID-19: a systematic review and meta-analysis. *Journal of medical virology* **93**, 257–261 (2021).

8. P. Slovic, "If i look at the mass i will never act: Psychic numbingpsychic numbing and genocidegenocide" in *Emotions and Risky Technologies*, (Springer, 2010), pp. 37–59.

9. S. T. Fiske, A. J. Cuddy, P. Glick, J. Xu, "A model of (often mixed) stereotype content: Competence and warmth respectively follow from perceived status and competition (2002)" in *Social Cognition*, (Routledge, 2018), pp. 171–222.

10. S. T. Fiske, A. J. Cuddy, P. Glick, Universal dimensions of social cognition: Warmth and competence. *Trends in cognitive sciences* **11**, 77–83 (2007).

11. C. W. Blatz, M. Ross, Principled ideology or racism: Why do modern racists oppose race-based social justice programs? *Journal of Experimental Social Psychology* **45**, 258–261 (2009).

For peer review only

STROBE Statement—Checklist of items that should be included in reports of ***cross-sectional studies***

	Item No	Recommendation	Page number
**Title and abstract**	1	(*a*) Indicate the study's design with a commonly used term in the title or the abstract	1
		(*b*) Provide in the abstract an informative and balanced summary of what was done and what was found	2
**Introduction**			
Background/rationale	2	Explain the scientific background and rationale for the investigation being reported	3
Objectives	3	State specific objectives, including any prespecified hypotheses	3
**Methods**			
Study design	4	Present key elements of study design early in the paper	4
Setting	5	Describe the setting, locations, and relevant dates, including periods of recruitment, exposure, follow-up, and data collection	4
Participants	6	(*a*) Give the eligibility criteria, and the sources and methods of selection of participants	5
Variables	7	Clearly define all outcomes, exposures, predictors, potential confounders, and effect modifiers. Give diagnostic criteria, if applicable	5
Data sources/ measurement	8*	For each variable of interest, give sources of data and details of methods of assessment (measurement). Describe comparability of assessment methods if there is more than one group	5
Bias	9	Describe any efforts to address potential sources of bias	4
Study size	10	Explain how the study size was arrived at	4
Quantitative variables	11	Explain how quantitative variables were handled in the analyses. If applicable, describe which groupings were chosen and why	5 to 6
Statistical methods	12	(*a*) Describe all statistical methods, including those used to control for confounding	6
		(*b*) Describe any methods used to examine subgroups and interactions	BAME: 7, Racism: 7 (Supplementary Figure 3)
		(*c*) Explain how missing data were addressed	NA
		(*d*) If applicable, describe analytical methods taking account of sampling strategy	NA
		(*e*) Describe any sensitivity analyses	NA
**Results**			
Participants	13*	(a) Report numbers of individuals at each stage of study—eg numbers potentially eligible, examined for eligibility, confirmed eligible, included in the study, completing follow-up, and analysed	5
		(b) Give reasons for non-participation at each stage	NA
		(c) Consider use of a flow diagram	NA

For peer review only - http://bmjopen.bmj.com/site/about/guidelines.xhtml

Descriptive data	14*	(a) Give characteristics of study participants (eg demographic, clinical, social) and information on exposures and potential confounders	4, Table 1
		(b) Indicate number of participants with missing data for each variable of interest	NA
Outcome data	15*	Report numbers of outcome events or summary measures	6 to 7
Main results	16	(*a*) Give unadjusted estimates and, if applicable, confounder-adjusted estimates and their precision (eg, 95% confidence interval). Make clear which confounders were adjusted for and why they were included	Unadjusted estimates with CIS: 7 to 8
		(*b*) Report category boundaries when continuous variables were categorized	Modern Racism Scale: 8
		(*c*) If relevant, consider translating estimates of relative risk into absolute risk for a meaningful time period	NA
Other analyses	17	Report other analyses done—eg analyses of subgroups and interactions, and sensitivity analyses	6 to 8
**Discussion**			
Key results	18	Summarise key results with reference to study objectives	8
Limitations	19	Discuss limitations of the study, taking into account sources of potential bias or imprecision. Discuss both direction and magnitude of any potential bias	10
Interpretation	20	Give a cautious overall interpretation of results considering objectives, limitations, multiplicity of analyses, results from similar studies, and other relevant evidence	9-11
Generalisability	21	Discuss the generalisability (external validity) of the study results	9 to 11
**Other information**			
Funding	22	Give the source of funding and the role of the funders for the present study and, if applicable, for the original study on which the present article is based	11

*Give information separately for exposed and unexposed groups.

Note: An Explanation and Elaboration article discusses each checklist item and gives methodological background and published examples of transparent reporting. The STROBE checklist is best used in conjunction with this article (freely available on the Web sites of PLoS Medicine at http://www.plosmedicine.org/, Annals of Internal Medicine at http://www.annals.org/, and Epidemiology at http://www.epidem.com/). Information on the STROBE Initiative is available at www.strobe-statement.org.

For peer review only
